# Synthesis of Bis-Glyoxylamide Peptidomimetics Derived from Bis-*N*-acetylisatins Linked at C5 by a Methylene or Oxygen Bridge

**DOI:** 10.3390/molecules24234343

**Published:** 2019-11-27

**Authors:** Venty Suryanti, Ruonan Zhang, Vina Aldilla, Mohan Bhadbhade, Naresh Kumar, David StC Black

**Affiliations:** 1 Department of Chemistry, Faculty of Mathematics and Natural Sciences, Sebelas Maret University, Jl. Ir. Sutami 36A Surakarta, Jawa Tengah 57126, Indonesia; venty@mipa.uns.ac.id; 2 School of Chemistry, The University of New South Wales, Sydney, NSW 2052, Australia; zhrn_516@hotmail.com (R.Z.); vina.r.aldilla@student.unsw.edu.au (V.A.); n.kumar@unsw.edu.au (N.K.); 3 Solid State and Elemental Analysis Unit, Mark Wainwright Analytical Centre, Division of Research, The University of New South Wales, Sydney, NSW 2052, Australia; m.bhadbhade@unsw.edu.au

**Keywords:** antimicrobial peptides, *N*-acetylisatins, glyoxylamides, peptidomimetics, quorum sensing

## Abstract

The bis-glyoxylamide peptidomimetics have been synthesized from bis-*N*-acetylisatins linked at C5 by ring-opening with alcohols, amines, and amino acid methyl ester hydrochlorides. X-ray images of single crystals of bis-glyoxylamide peptidomimetics have been obtained.

## 1. Introduction

Many peptides show strong biological activities and are considered as a potential source of therapeutic agents. Prior work in this field has been well reviewed and describes examples of bioactive compounds [1,2]. However, their use as therapeutics has some limitations, such as poor absorption, low bioavailability, inability to cross cell membranes, and in vivo instability [3,4]. To address these issues, peptidomimetics are designed by mimicking existing peptide structures and/or function but whose backbone is not only based on α-amino acids. The modification of the peptide backbone by incorporation of a large range of non-proteinogenic amino acids enhances the proteolytic stability of the molecules, increasing their function as drugs against diseases, such as viral and bacterial infections, cancer and autoimmune diseases. Thus, synthesis of such peptidomimetics is a critical and promising approach in the development of novel pharmaceutical compounds [5,6,7,8,9,10,11,12,13]. 

Our group has reported libraries of mono-glyoxylamides derived from *N*-acylisatins [14,15,16]. Acyclic and cyclic glyoxamide derivatives resulting from ring-opening reactions of *N*-chloroacetylisatins expressed quorum sensing (QS) inhibition activity against *P. aeruginosa* MH602 and *E. coli* MT102 [17]. The ring-opening reactions of *N*-naphthoylisatins with amines and amino acids have been examined to give guanidine-embedded amphipathic glyoxamide-based peptidomimetics and the example of methyl {2-[2-(2-naphthylamido)phenyl]-2-oxoacetyl}arginylargininate dihydrochloride demonstrated the greatest disruption of established biofilms by 60–65% in *S. aureus*, *P. aeruginosa*, and *S. marcescens* [18]. Related *N*-sulfonylphenylglyoxamide derivatives have been synthesized as small molecular antimicrobial peptide (AMP) mimics by ring-opening reactions of *N*-sulfonylisatins with *N*,*N*-dimethylethane-1,2-diamine or *N*,*N*-dimethylpropane-1,3-diamine and were converted into their hydrochloride or iodide salts [19].

In our previous research, we reported the synthesis of bis-glyoxylamide peptidomimetics from oxalyl-bis-isatins and bis-acylisatins linked through their carbonyl groups and some analogs showed potent QS inhibitory activity against Gram-positive bacteria [20,21]. In order to expand the library of bis-glyoxylamides, we have designed novel bis-glyoxylamide peptidomimetics from bis-*N*-acetylisatins linked at C5. In this study, we synthesized bis-*N*-acetylisatins linked by a C5 methylene or oxygen bridge and their nucleophilic ring-opening reactions were examined with alcohols, amines, and amino acid methyl ester hydrochlorides. 

## 2. Results and Discussion

### 2.1. Synthesis of bis-N-acetylisatins

**Scheme 1:**
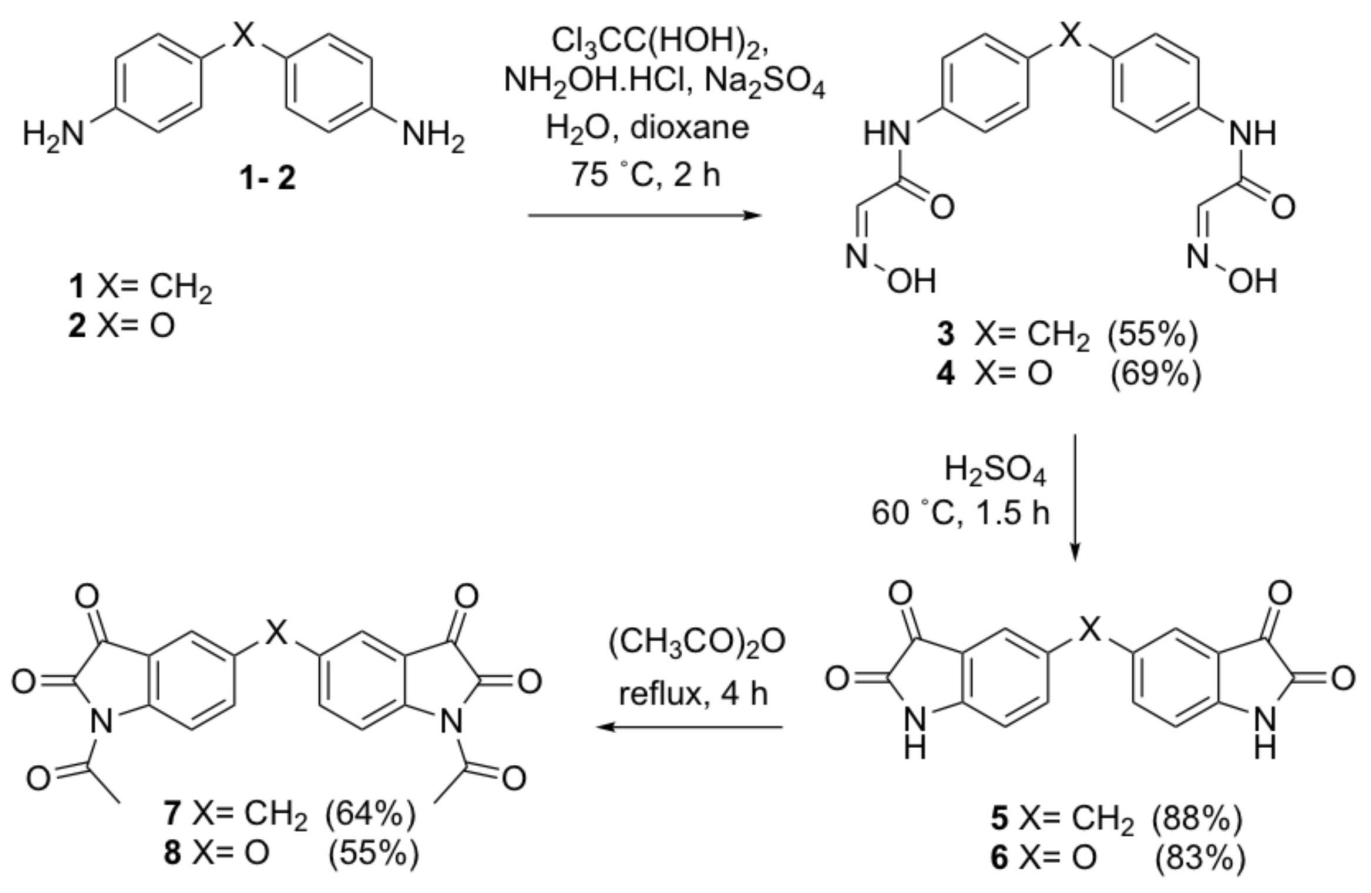
Synthesis of bis-N-acetylisatins 7 and 8.

The bis-isatyl methane **5** and the bis-isatyl ether **6** have been reported many years ago, but without any spectroscopic information [22,23,24]. Therefore, details of our modified syntheses are included here. As a starting point, the intermediate bis-isonitrosoacetanilide **3** was synthesized by applying the Sandmeyer isonitrosoacetanilide isatin synthesis [25]. The starting material of methylene bridged, 4,4’-methylenedianiline **1** was initially dissolved in aqueous hydrochloric acid and heated in the presence of chloral hydrate and hydroxylamine. The product crystallized out after being left overnight at room temperature and was recrystallized from ethyl acetate to give bis-isonitrosoacetanilide **3** in 10% yield as yellow crystals. A consideration of the mechanism shows the generation of a large amount of HCl in the reaction. Therefore, in an attempt to increase the yield, the concentrated HCl solvent was replaced by dioxane. The 4,4’-methylenedianiline **1** was dissolved in the minimum amount of 1,4-dioxane instead of hydrochloric acid and this led to the successful synthesis of the target bis-isonitrosoacetanilide ***3*** to 55% yield (Scheme 1).

The reaction was continued by dissolving bis-isonitrosoacetanilide **3** in concentrated sulfuric acid and stirring the solution to afford the bis-isatin **5** in high yield (88%). The bis-isatin **5** was then suspended in acetic anhydride and heated at reflux to generate the target bis-*N-*acetylisatin **7** in 64% yield. In the ^1^H NMR spectrum of compound **7**, the two CH_3_ groups resonated as a singlet that integrated for 6H at δ 2.59, the CH_2_ appeared as a singlet at δ 4.11 and the aromatic protons resonated as a singlet at δ 7.69, a doublet of doublet at δ 7.73, and a doublet at δ 8.85.

In the same manner, the structurally similar bis-*N-*acetylisatins **8** with oxygen as the linkage was generated from 4,4’-oxydianiline **2**. The ring closure of intermediate bis-isonitrosoacetanilide **4** gave bis-isatin **6** in 83% yield. The targeted bis-*N-*acetylisatin **8** was obtained in 55% yield by acetylating bis-isatin **6** with acetic anhydride (Scheme 1). Following the synthesis of bis-*N-*acetylisatins **7** and **8**, their nucleophilic ring-opening reactions were then carried out with alcohols, amines and amino acid methyl ester hydrochlorides, in order to generate the new class of peptide mimics. 

### 2.2. Ring-Opening Reactions of bis-N-acetylisatins with Alcohols

**Scheme 2:**
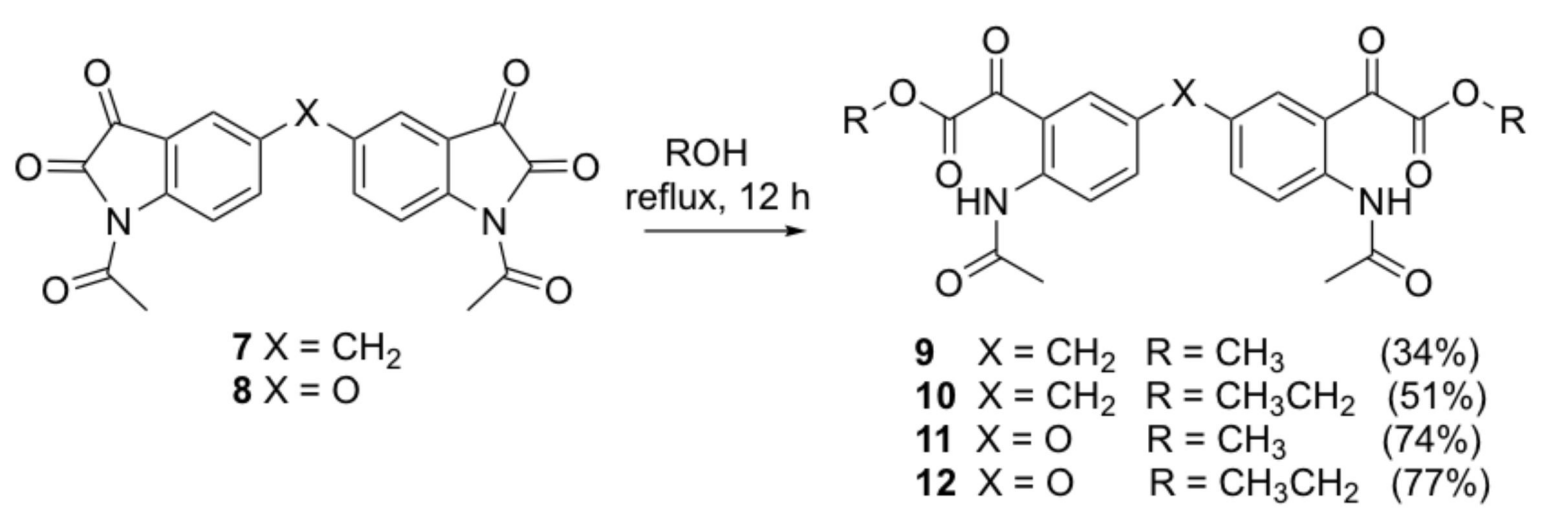
Ring-opening reactions of bis-N-acetylisatins 7 and 8 with alcohols.

The ring-opening reactions of bis-*N*-acetylisatins **7** and **8** were initially examined with the alcohols MeOH and EtOH. Bis-*N*-acetylisatin **7** was dissolved in anhydrous methanol and the solution was stirred for 24 h at room temperature. The resulting yellow crystals were recrystallized with some loss to give methyl bis-glyoxylacetate **9** in 34% yield (Scheme 2). ^1^H NMR spectrum of bis-glyoxylacetate **9** revealed three singlets at δ 3.89, 3.90, and 10.91 corresponding to CH_3_, CH_2,_ and two NH protons, respectively. The resonances of aromatic protons appeared as a singlet at δ 7.37 and two doublets at δ 7.35 and 8.66.

The analogous reaction with ethanol did not show the formation of any new products over 24 h, as confirmed by TLC analysis. Thus, the reaction mixture was heated at reflux for 3 h and during that time the solution turned dark yellow. A yellow solid precipitated upon cooling to room temperature and was recrystallized to give bis-glyoxylacetate **10** in 51% as yellow crystals. The analogous reactions of the bis-*N*-acetylisatin **8** afforded the bis-glyoxylacetates **11** and **12**.

### 2.3. Ring-Opening Reactions of bis-N-acetylisatins with Amines

**Scheme 3:**
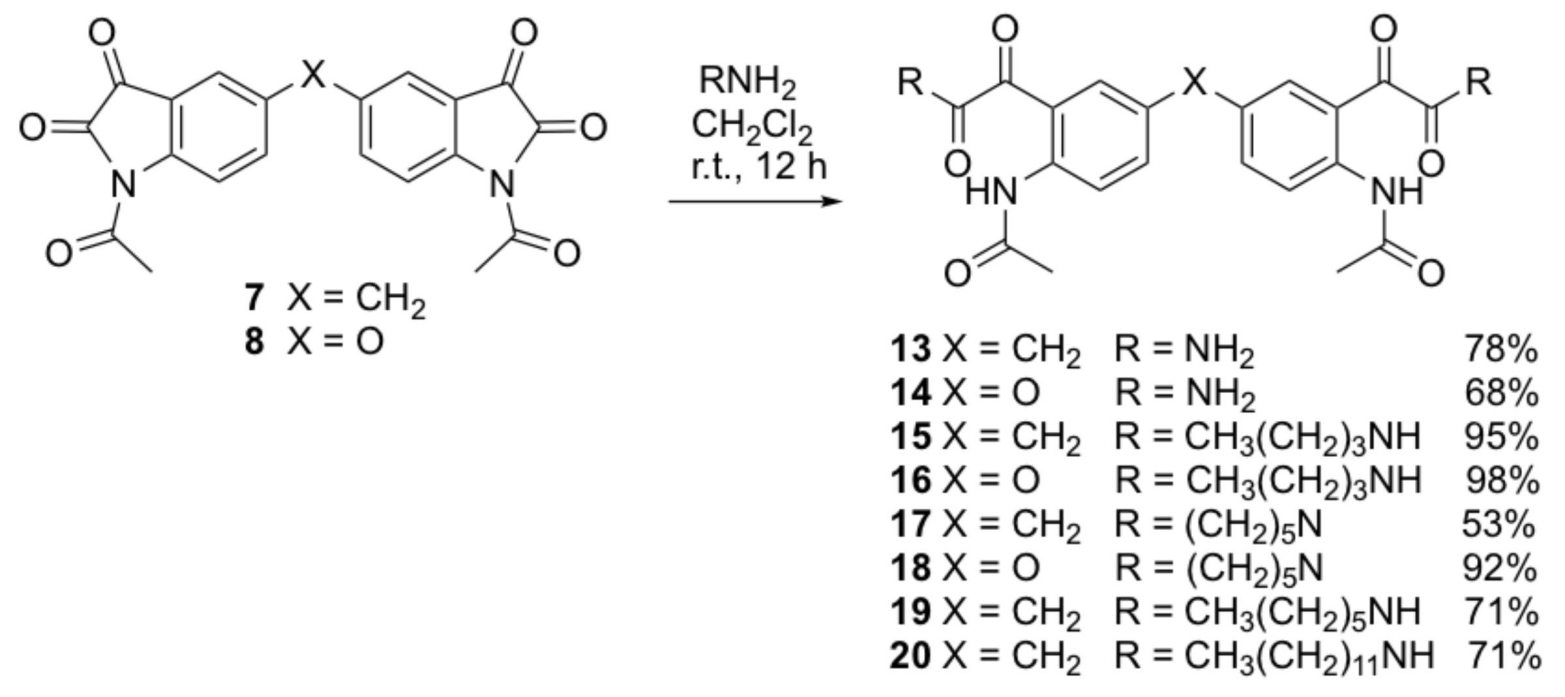
Ring-opening reactions of bis-N-acetylisatins 7 and 8 with amines.

The primary and secondary amines, butylamine and piperidine, were reacted with the bis-*N*-acetylisatins **7** and **8** by stirring the reaction mixtures for 5 h at room temperature. The bis-glyoxylamide products **15**–**18** were obtained in yields of 53–98% (Scheme 3). In the ^1^H NMR spectrum of bis-glyoxylamide **15**, the CH_2_ linkage was present as a singlet at δ 3.91, the butylamine carbon chain resonated as four multiplets at δ 0.86–0.88, 1.28–1.35, 1.47–1.52, and 3.28–3.32, the aromatic protons appeared as a doublet of doublets at δ 7.33 and two doublets at δ 8.07 and 8.50, and four NH protons resonated as two singlets at δ 6.81 and 10.79.

The ring-opening reactions of bis-*N*-acetylisatins **8** using primary amines with longer alkyl chains, such as hexylamine and dodecylamine were also investigated, but these reactions did not proceed at room temperature. However, by heating the reaction mixtures at reflux overnight the bis-glyoxylamide products **19** and **20** were obtained as yellow solids in moderate yields.

### 2.4. Ring-Opening Reactions of bis-N-acetylisatins with Amino Acid Methyl Esters

**Scheme 4:**
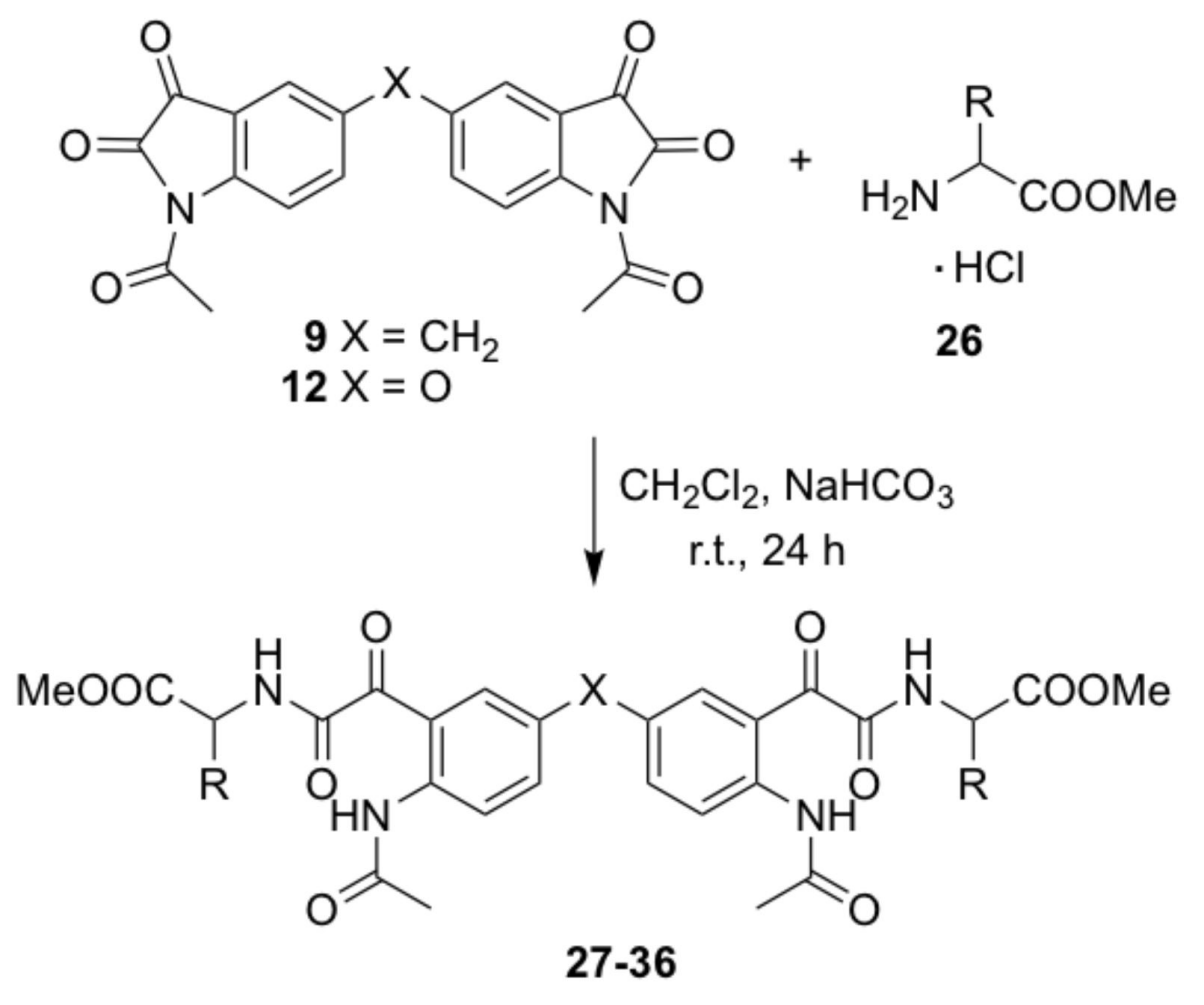
Ring-opening reactions of bis-N-acetylisatins 7 and 8 with amino acid methyl ester hydrochlorides.

The reaction mixtures of bis-*N*-acetylisatins **7** and **8** with amino acid methyl ester hydrochloride salts and sodium hydrogen carbonate in dichloromethane or acetonitrile at room temperature overnight gave the corresponding bis-glyoxylamide peptide mimics **21**–**30** in yields of 26–80% as yellow solids (Scheme 4, Table 1). The ^1^H NMR spectrum of compound **25** was typical for these peptide mimics and revealed two singlet peaks at δ 7.94 and 10.87, which integrated for 2H each and corresponded to the four NH groups. The valine subunit was identified by two multiplets and one doublet at δ 0.86–0.92, 2.16–2.21, and 4.54 which corresponded to the four methyl groups, the α-proton and the β-proton, respectively. The aromatic protons appeared as a multiplet at δ 7.31–7.41 and a doublet at δ 8.51. 

The bis-glyoxylamide peptidomimetics were recrystallized from a range of solvents via slow evaporation of the solvent at room temperature. Crystals suitable for X-ray crystallography were successfully obtained for compounds **22**–**24** from methanol. Single crystal X-ray structure determination was carried out on compounds **22**–**24**.

**Figure 1:**
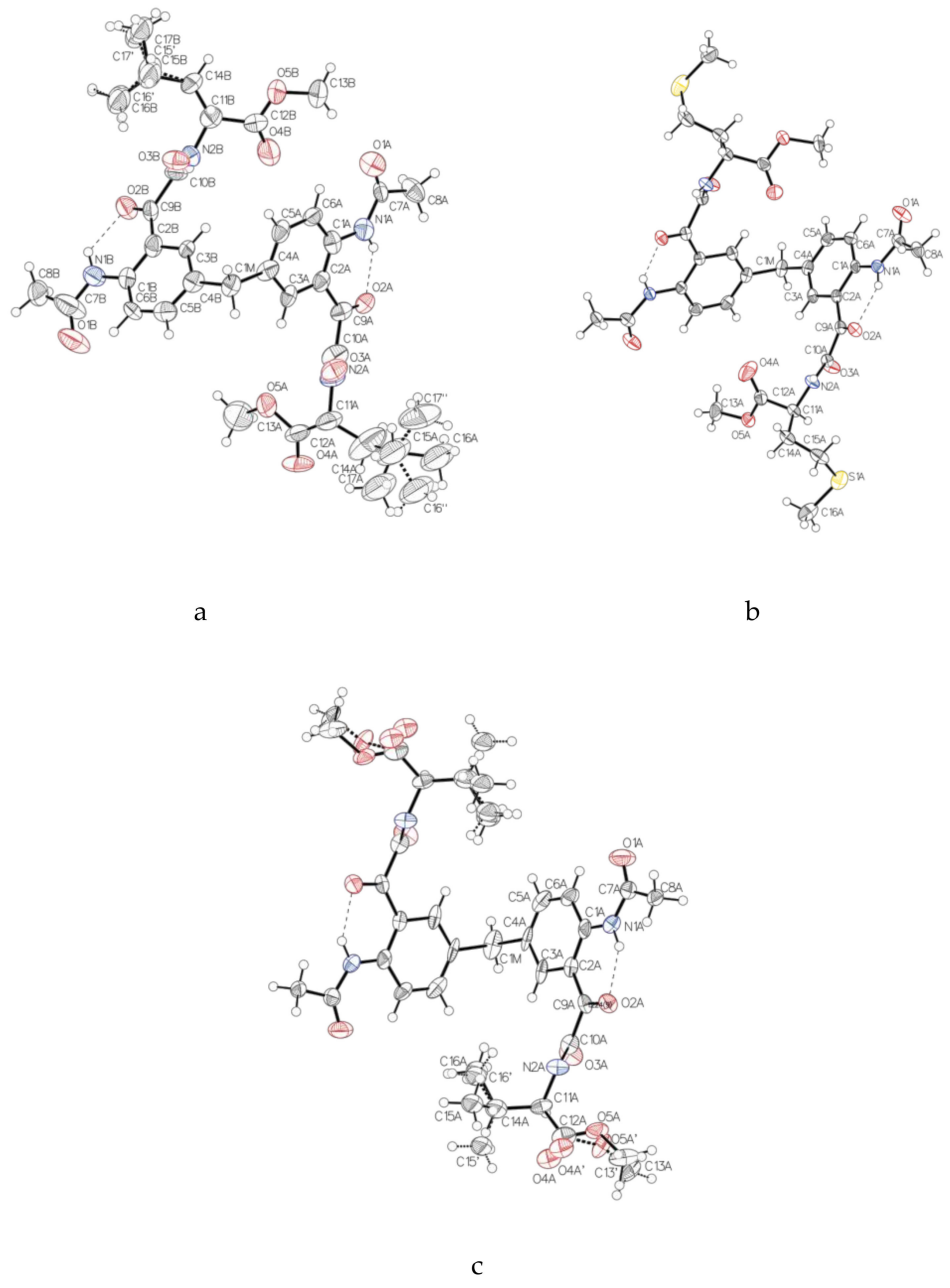
ORTEP diagrams of compound **22** (**a**), compound **23** (**b**), and compound **24** (**c**).

Figure 1 shows the Oak Ridge Thermal Ellipsoid Plot (ORTEP) diagrams for the molecules **22** and for **23** and **24** (only one of the diastereoisomers similar to **22** is shown). In all the compounds, the bulky substituents, probably to avoid a steric clash, adopt *trans* positions with respect to each other. The side chains are orientationally disordered in **22** and **24**, while this is not observed in the structure of **23** that contains heavy sulfur atom. The core of the structure is well ordered. Strong intramolecular N-H···O hydrogen bonds (shown as dotted lines in Figure 1) maintain the planarity of each half of the molecule. Weaker intramolecular C-H···O (C6-H···O1) contacts restrict the planarity of these moieties. However, the overall molecular framework is angled because of the central CH_2_ link.

**Figure 2:**
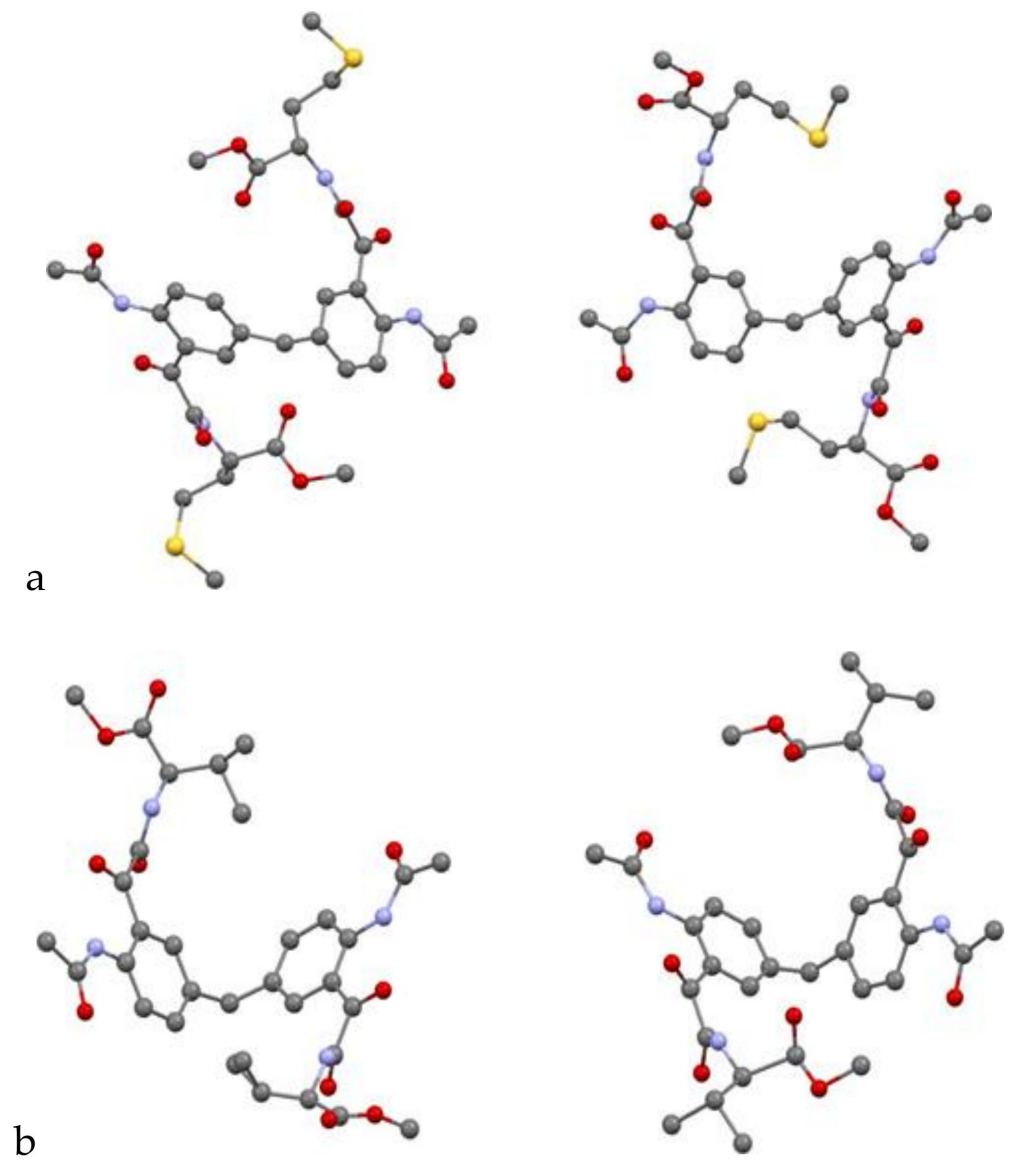
Two molecules in the asymmetric unit of **23** (**a**) and **24** (**b**).

There is only one molecule in the asymmetric unit of 22 in orthorhombic space group P21212, whereas there are two molecules (diastereoisomers) in the asymmetric unit of 23 as well as in 24 in monoclinic space group C2 (Figure 2). The ring-opening reaction takes place with retention of configuration, as shown by optical rotation measurements. One point that establishes this issue unambiguously is the space group in which these compounds have crystallized. They are all chiral space groups (P2(1)2(1)2 and two in C2), which by the absence of mirror (or glide) symmetry, allow only one of the enantiomers.

## 3. Materials and Methods

Melting points were measured using a Reichert microscope (Gallenkamp hot stage apparatus; Mettler-Toledo Ltd, Sydney, Australia) and are uncorrected. Infrared spectra were recorded with a Thermo Nicolet 370 FTIR spectrometer with the sample prepared as a KBr pellet. NMR data were recorded using a Bruker DPX300 instrument (^1^H 300 MHz, ^13^C 75.6 MHz) (Bruker Pty Ltd, Preston, Victoria, Australia) at 25 °C and reported as chemical shift (δ) relative to SiMe_4_. High resolution mass spectrometric analysis was carried out at the Biomedical Mass Spectrometry Facility, UNSW, and the spectra were recorded on Q-TOF Ultima API (Micromass; Waters, Rydalmere, NSW, Australia). Gravity column chromatography was carried out using Merck 230–400 mesh ASTM silica gel. Supplementary Materials contains NMR spectroscopic data.

*N,N’-[4,4’-bis(4,1-phenylene)] bis [2-(hydroxyimino)acetamide]methylene* (**3**). Concentrated sodium sulfate aqueous solution (175 mL) and a solution of 4,4′-methylenedianiline 1 (10 g, 50.4 mmol) in dioxane (15 mL) was added to a stirred solution of chloral hydrate (18.5 g, 126.1 mmol) in water (175 mL). The reaction mixture was heated slowly to 75 °C until a yellow precipitate was formed. A solution of hydroxylamine hydrochloride (8.8 g, 126.7 mmol) in water (55 mL) was then added into the resulting suspension. The temperature was increased to 75 °C and the reaction mixture was stirred for a further 2 h. The reaction mixture was cooled to room temperature and left to stand overnight. The crude product was collected by filtration and recrystallized from ethyl acetate to give the title compound as yellow crystals (9.44 g, 55%); mp 272–274 °C; IR (KBr): υ_max_ 3194, 2919, 2613, 1675, 1606, 1547, 1510, 1443, 1412, 1252, 1100, 1023, 999, 820, 765, 622, 510 cm^−1^; ^1^H NMR (DMSO-d_6_, 300 MHz): δ 12.13 (bs, 2H, 2 x OH) (disappears on D_2_O exchange), 10.14 (bs, 2H, 2 x NH) (disappears on D_2_O exchange), 7.65 (s, 2H, 2 x CHNOH), 7.59 (d, J = 8.5 Hz, 4H, ArH), 7.16 (d, J = 8.7 Hz, 4H, ArH), 3.85 (s, 2H, ArCH_2_Ar); ^13^C NMR (DMSO-d_6_, 75.6 MHz): δ 160.4 (CO), 144.4 (CHNOH), 137.2 (ArC), 136.8 (ArC), 129.2 (ArC), 120.3 (ArC), 40.2 (ArCH_2_Ar); HRMS (+ESI) *m*/*z* [M + Na]^+^ calcd for C_17_H_16_N_4_NaO_4_: 363.1069; found: 363.1055.

*N,N’-[4,4’-bis(4,1-phenylene)]bis [2-(hydroxyimino)acetamide]oxide* (**4**). This compound was prepared by the same method as compound **3** from 4,4′-oxydianiline 2 (10 g, 50.0 mmol) as orange needles (11.8 g, 69%); mp 221–223 °C; IR (KBr): υ_max_ 3185, 2931, 2622, 1669, 1596, 1521, 1510, 1433, 1410, 1232, 1096, 1021, 1000, 822, 764, 619 cm^−1^; ^1^H NMR (DMSO-*d_6_*, 300 MHz): δ 12.22 (bs, 2H, 2 x OH) (disappears on D_2_O exchange), 10.22 (bs, 2H, 2 x NH) (disappears on D_2_O exchange), 7.69 (d, *J* = 9.0 Hz, 4H, ArH), 7.67 (s, 2H, 2 x CHNOH), 6.99 (d, *J* = 9.0 Hz, 4H, ArH); ^13^C NMR (DMSO-*d_6_*, 75.6 MHz): δ 153.2 (ArC), 160.4 (CO), 144.4 (CHNOH), 137.2 (ArC), 121.9 (ArC), 119.1 (ArC); HRMS (+ESI) *m*/*z* [M + Na]^+^ calcd for C_16_H_14_N_4_NaO_5_: 365.0862; found: 365.0851.

*5,5’-Methylenediindoline-2,3-dione* (**5**). Compound **3** (5 g, 14.7 mmol) in small portions at 60 °C was added to concentrated sulfuric acid (20 mL). The deep red reaction mixture was stirred at 60 °C for a further 30 min. Chilled water (100 mL) was added to quench the reaction. The title compound was collected by filtration as a red solid (3.96 g, 88%); mp > 300 °C; IR (KBr): υ_max_ 3450, 3109, 1622, 1472, 1271, 1200, 1141, 906, 833, 745, 731, 711, 660, 622 cm**^−^**^1^; ^1^H NMR (DMSO-*d_6_*, 300 MHz): δ 10.99 (bs, 2H, 2 x NH) (disappears on D_2_O exchange), 7.51 (dd, *J* = 1.8, 8.1 Hz, 2H, ArH), 7.42 (s, 2H, ArH), 6.86 (d, *J* = 8.2 Hz, 2H, ArH), 3.89 (s, 2H, ArCH_2_Ar); ^13^C NMR (DMSO-*d_6_*, 75.6 MHz): δ 184.8 (COCONH), 159.8 (COCONH), 149.4 (ArC), 138.9 (ArC), 136.2 (ArC), 124.9 (ArC), 118.3 (ArC), 112.7 (ArC), 39.3 (ArCH_2_Ar); HRMS (+ESI) *m*/*z* [M + Na]^+^ calcd for C_17_H_10_N_2_NaO_4_: 329.0538; found: 329.0541.

*5,5’-Oxydiindoline-2,3-dione* (**6**). This compound was prepared by the same method as compound 8 from compound 4 (5.0 g, 14.6 mmol) as a red solid (4.02 g, 83%); mp > 300 °C; IR (KBr): υ_max_ 3457, 3111, 1743, 1621, 1472, 1199, 1145, 907, 839, 744, 712, 660, 623 cm**^−^**^1^; ^1^H NMR (DMSO-*d_6_*, 300 MHz): δ 11.02 (bs, 2H, 2 x NH) (disappears on D_2_O exchange), 7.30 (dd, *J* = 2.6, 2H, 8.5 Hz, ArH), 7.09 (s, 2H, ArH), 6.93 (d, *J* = 8.5 Hz, 2H, ArH); ^13^C NMR (DMSO-*d_6_*, 75.6 MHz): δ 184.4 (COCONH), 159.9 (COCONH), 149.4 (ArC), 147.1 (ArC), 129.0 (ArC), 118.9 (COCONH), 114.7 (ArC), 114.0 (ArC); HRMS (+ESI) *m*/*z* [M + Na]^+^ calcd for C_16_H_8_N_2_NaO_4_: 331.0331; found: 331.0318.

*Bis(1-acetylindoline-2,3-dione)methylene* (**7**). A suspension of compound 5 (2 g, 6.5 mmol) in acetic anhydride (30 mL) was heated to reflux for 4 h. The deep red solution was cooled to room temperature. The excess acetic anhydride was removed under vacuum. The resulting brown oily residue was redissolved in ethyl acetate (20 mL) and washed with water (2 × 20 mL) and brine (2 × 20 mL). The organic layer was dried over anhydrous sodium sulfate and concentrated under vacuum. The crude product was purified by flash column chromatography (dichloromethane) to yield the title compound as a bright yellow solid (1.63 g, 64%); mp 289–291 °C; IR (KBr): υ_max_ 1785, 1712, 1756, 1617, 1588, 1481, 1443, 1371, 1346, 1306, 1294, 1250, 1227, 1201, 1168, 1124, 1042, 996, 659, 598 cm**^−^**^1^; ^1^H NMR (CDCl_3_, 300 MHz): δ 8.22 (d, *J* = 8.5 Hz, 2H, ArH), 7.73 (dd, *J* = 1.9, 8.5 Hz, 2H, ArH), 7.69 (s, 2H, ArH), 4.11 (s, 2H, ArCH_2_Ar), 2.59 (s, 6H, 2 x COCH_3_); ^13^C NMR (CDCl_3_, 75.6 MHz): δ 180.5 (COCONH), 170.0 (COCH_3_), 158.7 (COCONH), 146.8 (ArC), 138.3 (ArC), 124.5 (ArC), 124.5 (ArC), 120.5 (ArC), 117.8 (ArC), 39.2 (ArCH_2_Ar), 26.3 (COCH_3_); HRMS (+ESI) *m*/*z* [M + Na]^+^ calcd for C_21_H_14_N_2_NaO_6_: 413.0750; found: 413.0713.

*5,5’-Bis(1-acetylindoline-2,3-dione)oxide* (**8**). This compound was prepared by the same method as compound **7** from compound **6** (2.0 g, 6.5 mmol) as a bright yellow solid (1.40 g, 55%); mp > 300 °C; IR (KBr): υ_max_ 3429, 1783, 1747, 1702, 1617, 1470, 1371, 1330, 1312, 1293, 1266, 1241, 1156, 850, 599, 468 cm**^−^**^1^; ^1^H NMR (CDCl_3_, 300 MHz): δ 8.31 (d, *J* = 9.2 Hz, 2H, ArH), 7.51 (dd, *J* = 2.9, 8.9 Hz, 2H, ArH), 7.31 (s, 2H, ArH), 2.57 (s, 6H, 2 x COCH_3_); ^13^C NMR (CDCl_3_, 75.6 MHz): δ 179.9 (COCONH), 170.0 (COCH_3_), 158.6 (COCONH), 154.1 (ArC), 144.4 (ArC), 128.3 (ArC), 121.8 (ArC), 119.7 (ArC), 113.9 (ArC), 26.2 (COCH_3_); HRMS (+ESI) *m*/*z* [M + Na]^+^ calcd for C_20_H_12_N_2_NaO_7_: 415.0542; found: 415.0526.

*Dimethyl 2,2’-[5,5’-bis(2-acetamido-5,1-phenylene)]bis(2-oxoacetate)methylene* (**9**). A solution of compound **7** (0.15 g, 0.38 mmol) in anhydrous methanol (20 mL) was stirred at room temperature for 24 h. A massive yellow precipitate formed. The crude compound was collected by filtration and recrystallized from *n*-hexane/DCM to afford the title compound as a yellow solid (0.059 g, 34%); mp 162–164 °C; IR (KBr): υ_max_ 3303, 1747, 1733, 1702, 1652, 1595, 1528, 1420, 1340, 1295, 1250, 1226, 1160, 1013 cm**^−^**^1^; ^1^H NMR (CDCl_3_, 300 MHz): δ 10.91 (bs, 2H, 2 x NH) (disappears on D_2_O exchange), 8.66 (d, *J* = 8.7 Hz, 2H, ArH), 7.37 (s, 2H, ArH), 7.35 (d, *J* = 2.1 Hz, 2H, ArH), 3.90 (s, 2H, ArCH_2_Ar), 3.89 (s, 6H, 2 x COOCH_3_), 2.18 (s, 6H, 2 x COCH_3_); ^13^C NMR (CDCl_3_, 75.6 MHz): δ 190.4 (COCH_3_), 169.8 (COCOO), 164.1 (COCOO), 141.7 (ArC), 138.0 (ArC), 134.5 (ArC), 133.6 (ArC), 121.6 (ArC), 117.6 (ArC), 53.4 (COOCH_3_), 40.2 (ArCH_2_Ar), 25.9 (COCH_3_); HRMS (+ESI) *m*/*z* [M + Na]^+^ calcd for C_23_H_22_N_2_NaO_8_: 477.1274; found: 477.1243.

*Diethyl 2,2’-[5,5’-bis(2-acetamido-5,1-phenylene)]bis(2-oxoacetate)methylene* (**10**). A solution of compound **7** (0.15 g, 0.38 mmol) in absolute ethanol (20 mL) was heated to reflux for 12 h. The reaction mixture was allowed to cool to room temperature and a massive yellow precipitate formed. The crude compound was collected by filtration and recrystallized from *n*-hexane/DCM to afford the title compound as a yellow solid (0.095 g, 51%); mp 162–164 °C; IR (KBr): υ_max_ 3317, 1749, 1738, 1705, 1648, 1617, 1590, 1493, 1399, 1367, 1295, 1243, 1211, 1089, 1007 cm**^−^**^1^; ^1^H NMR (CDCl_3_, 300 MHz): δ 11.02 (bs, 2H, 2 x NH) (disappears on D_2_O exchange), 8.73 (d, *J* = 9.0 Hz, 2H, ArH), 7.44 (s, 2H, ArH), 7.42 (dd, *J* = 2.2, 9.0 Hz, 2H, ArH), 4.41 (q, *J* = 7.2 Hz, 4H, 2 x CH_2_CH_3_), 3.97 (s, 2H, ArCH_2_Ar), 2.24 (s, 6H, 2 x COCH_3_), 1.35 (t, *J* = 7.1 Hz, 6H, 2 x CH_2_CH_3_); ^13^C NMR (CDCl_3_, 75.6 MHz): δ 190.2 (COCH_3_), 169.3 (COCOO), 163.3 (COCOO), 141.2 (ArC), 137.4 (ArC), 134.0 (ArC), 133.1 (ArC), 121.1 (ArC), 117.1 (ArC), 62.6 (CH_2_CH_3_), 39.7 (ArCH_2_Ar), 25.4 (OCH_3_), 13.9 (CH_2_CH_3_); HRMS (+ESI) *m*/*z* [M + Na]^+^ calcd for C_25_H_26_N_2_NaO_8_: 505.1587; found: 505.1572. 

*Dimethyl 2,2’-[5,5’-bis(2-acetamido-5,1-phenylene)]bis(2-oxoacetate)oxide* (**11**). Compound **11** was prepared by the same method as compound **9** from compound **8** (0.15 g, 0.38 mmol) as a white solid (0.129 g, 74%); mp 159–161 °C; IR (KBr): υ_max_ 3456, 1750, 1737, 1704, 1655, 1587, 1520, 1488, 1410, 1286, 1257, 1237, 1217, 1154, 1016, 970, 783 cm**^−^**^1^; ^1^H NMR (CDCl_3_, 300 MHz): δ 10.82 (bs, 2H, 2 x NH) (disappears on D_2_O exchange), 8.72 (d, *J* = 9.1 Hz, 2H, ArH), 7.32 (d, *J* = 2.7 Hz, 2H, ArH), 7.22 (dd, *J* = 2.9, 9.2 Hz, 2H, ArH), 3.89 (s, 6H, 2 x COOCH_3_), 2.19 (s, 6H, 2 x COCH_3_); ^13^C NMR (CDCl_3_, 75.6 MHz): δ 189.6 (COCH_3_), 169.7 (COCOO), 163.7 (COCOO), 151.3 (ArC), 139.2 (ArC), 127.7 (ArC), 123.2 (ArC), 122.9 (ArC), 118.6 (ArC), 53.6 (COOCH_3_), 25.9 (COCH_3_); HRMS (+ESI) *m*/*z* [M + H]^+^ calcd for C_22_H_21_N_2_O_9_: 457.1247; found: 457.1230.

*Diethyl 2,2’-[5,5’-bis(2-acetamido-5,1-phenylene)]bis(2-oxoacetate)oxide* (**12**). Compound **12** was prepared by the same method as compound **10** from compound **8** (0.15 g, 0.38 mmol) as a yellow solid (0.143 g, 77%); mp 104–106 °C; IR (KBr): υ_max_ 3307, 2965, 1745, 1655, 1589, 1520, 1437, 1411, 1372, 1267, 1217, 1282, 1160, 1011, 842 cm**^−^**^1^; ^1^H NMR (CDCl_3_, 300 MHz): δ 10.92 (bs, 2H, 2 x NH) (disappears on D_2_O exchange), 8.79 (d, *J* = 9.3 Hz, 2H, ArH), 7.37 (d, *J* = 2.7 Hz, 2H, ArH), 7.35 (dd, *J* = 2.9, 9.3 Hz, 2H, ArH), 4.41 (q, *J* = 7.2 Hz, 4H, 2 x CH_2_CH_3_), 2.25 (s, 6H, 2 x COCH_3_), 1.36 (t, *J* = 7.1 Hz, 6H, 2 x CH_2_CH_3_); ^13^C NMR (CDCl_3_, 75.6 MHz): δ 189.6 (COCH_3_), 169.3 (COCOO), 162.9 (COCOO), 151.0 (ArC), 138.8 (ArC), 127.3 (ArC), 122.8 (ArC), 122.4 (ArC), 118.2 (ArC), 62.9 (CH_2_CH_3_), 25.4 (OCH_3_), 14.0 (CH_2_CH_3_); HRMS (+ESI) *m*/*z* [M + Na]^+^ calcd for C_24_H_24_N_2_NaO_9_: 507.1380; found: 507.1360.

*N,N’-{4,4’-bis-[2-(2-acetamidophenyl)-2-oxoacetamide]}methylene* (**13**). To a solution of compound **7** (0.15 g, 0.38 mmol) in dichloromethane (20 mL) was added concentrated ammonia solution (10 mL). The reaction mixture was stirred at room temperature for 30 min. A massive white precipitate formed. The crude product was collected by filtration and purified by recrystallization from methanol to yield the title compound **13** as a white solid (0.127 g, 78%); mp 229–232 °C; IR (KBr): υ_max_ 3310, 2933, 1692, 1641, 1588, 1522, 1466, 1411, 1359, 1310, 1279, 1201, 1177, 1132, 987, 900, 826, 735, 591 cm**^−^**^1^; ^1^H NMR (DMSO-*d_6_*, 300 MHz): δ 10.50 (bs, 2H, 2 x NH) (disappears on D_2_O exchange), 7.79 (s, 4H, 2 x NH_2_) (disappears on D_2_O exchange), 8.01 (s, 2H, ArH), 7.50 (d, *J* = 1.9 Hz, 2H, ArH), 7.44 (dd, *J* = 2.1, 8.5 Hz, 2H, ArH), 3.99 (s, 2H, ArCH_2_Ar), 2.04 (s, 6H, 2 x COCH_3_); ^13^C NMR (DMSO-*d_6_*, 75.6 MHz): δ 169.2 (COCONH_2_), 192.6 (COCH_3_), 166.3 (COCONH_2_), 137.4 (ArC), 136.4 (ArC), 134.8 (ArC), 131.8 (ArC), 124.3 (ArC), 122.3 (ArC), 40.0 (ArCH_2_Ar), 24.5 (OCH_3_); HRMS (+ESI) *m*/*z* [M + Na]^+^ calcd for C_21_H_20_N_4_NaO_6_: 447.1281; found: 447.1252.

*N,N’-{4,4’-bis-[2-(2-acetamidophenyl)-2-oxoacetamide]}oxide* (**14**). Compound **14** was prepared by the same method as compound **13** from compound **8** (0.15 g, 0.38 mmol) as a white solid (0.108 g, 66%); mp 183–186 °C; IR (KBr): υ_max_ 3309, 2940, 2859, 1701, 1639, 1588, 1521, 1463, 1416, 1368, 1326, 1285, 1237, 1215, 1181, 1165, 1142, 991, 942, 842, 753, 637 cm**^−^**^1^; ^1^H NMR (DMSO-*d_6_*, 300 MHz): δ 10.36 (bs, 2H, 2 x NH) (disappears on D_2_O exchange), 8.00 (s, 2H, ArH), 7.74 (s, 4H, 2 x NH_2_) (disappears on D_2_O exchange), 7.50 (d, *J* = 1.7 Hz, 2H, ArH), 7.12 (dd, *J* = 1.7, 8.0 Hz, 2H, ArH), 2.14 (s, 6H, 2 x COCH_3_); ^13^C NMR (DMSO-*d_6_*, 75.6 MHz): δ 193.4 (COCH_3_), 169.5 (COCONH_2_), 166.2 (COCONH_2_), 137.1 (ArC), 136.3 (ArC), 130.9 (ArC), 128.9 (ArC), 120.5 (ArC), 113.2 (ArC), 26.1 (OCH_3_); HRMS (+ESI) *m*/*z* [M + Na]^+^ calcd for C_20_H_18_N_4_NaO_7_: 449.1073; found: 447.1059.

*N,N’-{4,4’-bis-[2-(2-acetamidophenyl)-N-butyl-2-oxoacetamide]}methylene* (**15**). A solution of n-butylamine (0.20 mL, 2.0 mmol) in anhydrous dichloromethane (20 mL)was added to a stirred solution of compound **7** (0.15 g, 0.38 mmol) in anhydrous dichloromethane (30 mL). The reaction mixture was stirred at room temperature for 12 h. The organic layer was diluted with dichloromethane (30 mL) and extracted in aqueous hydrochloric acid (0.5 M, 2 × 40 mL) and water (3 x 40 mL). The organic layer was dried over anhydrous sodium sulfate and concentrated under vacuum. The crude compound was purified by gravity column chromatography (dichloromethane-ethyl acetate**/**3:1) to afford the title compound **15** as a yellow solid (0.196 g, 95%); mp 144–148 °C; IR (KBr): υ_max_ 3309, 2960, 1681, 1650, 1582, 1507, 1402, 1371, 1320, 1288, 1253, 1222, 1180, 1134, 1006, 833, 700 cm**^−^**^1^; ^1^H NMR (CDCl_3_, 300 MHz): δ 10.79 (bs, 2H, 2 x NH) (disappears on D_2_O exchange), 8.50 (d, *J* = 8.7 Hz, 2H, ArH), 8.07 (d, *J* = 2.0 Hz, 2H, ArH), 7.33 (dd, *J* = 2.0, 8.6 Hz, 2H, ArH), 6.81 (bs, 2H, 2 x NH butylamine) (disappears on D_2_O exchange), 3.91 (s, 2H, ArCH_2_Ar), 3.31 (dd, *J* = 6.8, 13.2 Hz, 4H, 2 x CH_2_CH_2_), 2.14 (s, 6H, 2 x COCH_3_), 1.47–1.52 (m, 4H, 2 x CH_2_CH_2_CH_2_CH_3_), 1.28–1.35 (m, 4H, 2 x CH_2_CH_3_), 0.87 (t, *J* = 7.6 Hz, 6H, 2 x CH_3_); ^13^C NMR (CDCl_3_, 75.6 MHz): δ 192.4 (COCH_3_), 169.5 (COCONH), 163.2 (COCONH), 140.9 (ArC), 137.4 (ArC), 134.9 (ArC), 134.7 (ArC), 121.6 (ArC), 119.4 (ArC), 40.4 (ArCH_2_Ar), 39.8 (CH_2_CH_2_CH_2_), 31.7 (CH_2_CH_2_CH_2_), 25.8 (OCH_3_), 20.4 (CH_2_CH_2_CH_3_), 14.1 (CH_3_); HRMS (+ESI) *m*/*z* [M + Na]^+^ calcd for C_29_H_36_N_4_NaO_6_: 559.2533; found: 559.2513. 

*N,N’-{4,4’-bis-[2-(2-acetamidophenyl)-N-butyl-2-oxoacetamide]}oxide* (**16**). Compound **16** was prepared by the same method as compound **15** from compound **8** (0.15 g, 0.38 mmol) and *n*-butylamine (0.20 mL, 2.0 mmol) as a yellow solid (0.202 g, 98%); mp 188–191 °C; IR (KBr): υ_max_ 3311, 2960, 1686, 1656, 1589, 1513, 1406, 1370, 1324, 1286, 1264, 1222, 1181, 1162, 1008, 847, 699 cm**^−^**^1^; ^1^H NMR (CDCl_3_, 300 MHz): δ 10.75 (bs, 2H, 2 x NH) (disappears on D_2_O exchange), 7.99 (d, *J* = 3.0 Hz, 2H, ArH), 8.58 (d, *J* = 9.3 Hz, 2H, ArH), 7.25 (dd, *J* = 3.4, 9.1 Hz, 2H, ArH), 6.97 (bs, 2H, 2 x NH butylamine) (disappears on D_2_O exchange), 3.34 (dd, *J* = 6.9, 13.2 Hz, 4H, 2 x CH_2_CH_2_), 2.21 (s, 6H, 2 x COCH_3_), 1.51–1.56 (m, 4H, 2 x CH_2_CH_2_CH_2_CH_3_), 1.32–1.39 (m, 4H, 2 x CH_2_CH_3_), 0.91 (t, *J* = 7.2 Hz, 6H, 2 x CH_3_); ^13^C NMR (CDCl_3_, 75.6 MHz): δ 191.3 (COCH_3_), 169.0 (COCONH), 162.4 (COCONH), 151.2 (ArC), 137.7 (ArC), 126.8 (ArC), 123.4 (ArC), 122.7 (ArC), 120.2 (ArC), 39.4 (CH_2_CH_2_CH_2_), 31.2 (CH_2_CH_2_CH_2_), 25.3 (OCH_3_), 20.0 (CH_2_CH_2_CH_3_), 13.7 (CH_3_); HRMS (+ESI) *m*/*z* [M + Na]^+^ calcd for C_28_H_34_N_4_NaO_7_: 561.2325; found: 561.2300.

*N,N’-[4,4’-bis-(N-{2-[2-oxo-2-(piperidin-1-yl)acetyl]phenyl}acetamide)]methylene* (**17**). Compound **17** was prepared by the same method as compound **15** from compound **7** (0.15 g, 0.38 mmol) and piperidine (0.20 mL, 2.0 mmol) as a yellow solid (0.114 g, 53%); mp 181–183 °C; IR (KBr): υ_max_ 2939, 2859, 1701, 1642, 1591, 1520, 1451, 1412, 1367, 1326, 1298, 1251, 1182, 1136, 988, 851, 784, 752 cm**^−^**^1^; ^1^H NMR (CDCl_3_, 300 MHz): δ11.15 (bs, 2H, 2 x NH) (disappears on D_2_O exchange), 8.66 (d, *J* = 8.8 Hz, 2H, ArH), 7.35 (dd, *J* = 1.8, 8.7 Hz, 2H, ArH), 7.28 (d, *J* = 1.8 Hz, 2H, ArH), 3.89 (s, 2H, ArCH_2_Ar), 3.55 (t, *J* = 5.5 Hz, 4H, 2 x NCH_2_), 3.13 (t, *J* = 5.5 Hz, 4H, 2 x NCH_2_), 2.18 (s, 6H, 2 x COCH_3_), 1.51–1.57 (m, 8H, 2 x CH_2_CH_2_CH_2_CH_2_CH_2_), 1.29–1.38 (m, 4H, 2 x CH_2_CH_2_CH_2_); ^13^C NMR (CDCl_3_, 75.6 MHz): δ 196.4 (COCH_3_), 169.8 (COCON), 164.5 (COCON), 141.3 (ArC), 137.6 (ArC), 135.0 (ArC), 133.6 (ArC), 121.4 (ArC), 118.5 (ArC), 47.5 (NCH_2_), 42.5 (NCH_2_), 40.1 (ArCH_2_Ar), 25.7 (NCH_2_CH_2_), 25.9 (OCH_3_), 25.7 (NCH_2_CH_2_), 24.6 (CH_2_CH_2_CH_2_); HRMS (+ESI) *m*/*z* [M + Na]^+^ calcd for C_31_H_36_N_4_NaO_6_: 583.2533; found: 583.2493.

*N,N’-[4,4’-bis-(N-{2-[2-oxo-2-(piperidin-1-yl)acetyl]phenyl}acetamide)]oxide* (**18**). Compound **18** was prepared by the same method as compound **15** from compound **8** (0.15 g, 0.38 mmol) and piperidine (0.20 mL, 2.0 mmol) as a yellow solid (0.198 g, 92%); mp 158–160 °C; IR (KBr): υ_max_ 3444, 3288, 1682, 1557, 1489, 1415, 1371, 1335, 1303, 1289, 1253, 1126, 929, 839, 669, 617, 559 cm**^−^**^1^; ^1^H NMR (CDCl_3_, 300 MHz): δ 11.08 (bs, 2H, 2 x NH) (disappears on D_2_O exchange), 8.75 (d, *J* = 8.8 Hz, 2H, ArH), 7.23 (dd, *J* = 2.8, 9.1 Hz, 2H, ArH), 7.20 (d, *J* = 1.9 Hz, 2H, ArH), 3.58 (t, *J* = 5.3 Hz, 4H, 2 x NCH_2_), 3.23 (t, *J* = 5.3 Hz, 4H, 2 x NCH_2_), 2.22 (s, 6H, 2 x COCH_3_), 1.63–1.65 (m, 4H, 2 x CH_2_CH_2_CH_2_), 1.51–1.57 (m, 8H, 2 x CH_2_CH_2_CH_2_CH_2_CH_2_); ^13^C NMR (CDCl_3_, 75.6 MHz): δ 195.2 (COCH_3_), 169.3 (COCON), 163.8 (COCON), 151.3 (ArC), 138.4 (ArC), 127.0 (ArC), 122.6 (ArC), 122.4 (ArC), 119.1 (ArC), 47.1 (NCH_2_), 42.2 (NCH_2_), 26.1 (NCH_2_CH_2_), 25.4 (OCH_3_), 25.3 (NCH_2_CH_2_), 24.2 (CH_2_CH_2_CH_2_); HRMS (+ESI) *m*/*z* [M + Na]^+^ calcd for C_30_H_34_N_4_NaO_7_: 585.2325; found: 585.2293.

*2,2′-[Methylene-bis(2-acetamido-5,1-phenylene)]bis(N-hexyl-2-oxoacetamide)* (**19**). A mixture of compound **7** (0.24 g, 0.61 mmol) and hexylamine (0.12 g, 1.22 mmol) in dichloromethane (30 mL) was heated at reflux overnight. After cooling to room temperature, the solvent was evaporated in vacuo and the crude product was purified by column chromatography using silica gel and *n*-hexane/DCM as eluent. The title compound **19** was obtained as an off-white solid (0.26 g, 71%); mp 182–184 °C; IR (KBr): υ_max_ 3305, 3053, 2928, 2855, 1694, 1650, 1585, 1518, 1465, 1411, 1369, 1293, 1223, 1176, 1006, 855, 811 cm**^−^**^1^; ^1^H NMR (CDCl_3_, 300 MHz): δ 10.89 (s, 2H, 2 x NHCO), 8.56 (d, *J* = 8.6 Hz, 2H, ArH), 8.16 (d, *J* = 2.0 Hz, 2H, ArH), 7.42 (dd, *J* = 8.7, 1.9 Hz, 2H, ArH), 6.98 (t, *J* = 5.5 Hz, 2H, 2 x CONH), 4.00 (s, 2H, ArCH_2_Ar), 3.47-3.49 (q, *J* = 6.7, 6.3 Hz, 4H, 2 x NHCH_2_CH_2_(CH_2_)_3_CH_3_), 2.25 (s, 6H, 2 x COCH_3_), 1.55–1.66 (m, 4H, 2 x NHCH_2_CH_2_(CH_2_)_3_CH_3_), 1.30–1.44 (m, 12H, 2 x NHCH_2_CH_2_(CH_2_)_3_CH_3_), 0.93 (t, *J* = 6.6 Hz, 6H, 2 x NHCH_2_(CH_2_)_4_CH_3_); ^13^C NMR (CDCl_3_, 75.6 MHz): δ 162.8, 169.1, 192.0 (6 x C=O), 121.2, 134.5, 136.9 (6 x ArCH), 119.0, 134.3, 140.5 (6 x ArC), 40.0 (2 x NHCH_2_), 39.7 (ArCH_2_Ar), 25.4 (2 x COCH_3_), 31.4 (2 x CH_2_(CH_2_)_4_CH_3_), 22.5, 26.6, 29.2, 14.0 (2 x CH_2_(CH_2_)_4_CH_3_); HRMS (ESI) *m*/*z* [M + H]^+^ calcd for C_33_H_45_N_4_O_6_: 593.3333; found: 593.3326.

*2,2′-[Methylene-bis(2-acetamido-5,1-phenylene)]bis(N-dodecyl-2-oxoacetamide)* (**20**). Compound **20** was prepared by the same method as compound **19** from compound **7** (0.24 g, 0.61 mmol) and dodecylamine (0.23 g, 1.22 mmol) as an off-white solid (0.26 g, 69%); mp 162–164 °C; IR (KBr): υ_max_ 3344, 3293, 2919, 2850, 1694, 1650, 1586, 1519, 1467, 1412, 1370, 1294, 1224, 1177, 1014, 855, 721 cm^−1^; ^1^H NMR (CDCl_3_, 300 MHz): δ 10.89 (s, 2H, 2 x NHCO), 8.54 (d, *J* = 8.5 Hz, 2H, ArH), 8.17 (d, *J* = 2.1 Hz, 2H, ArH), 7.45 (dd, *J* = 8.6, 2.12 Hz, 2H, ArH), 6.98 (t, *J* = 5.9 Hz, 2H, 2 x CONH), 4.03 (s, 2H, ArCH_2_Ar), 3.37–3.44 (q, *J* = 6.7, 6.8 Hz, 4H, 2 x NHCH_2_CH_2_(CH_2_)_9_CH_3_), 2.28 (s, 6H, 2 x COCH_3_), 1.56-1.66 (m, 4H, 2 x NHCH_2_CH_2_(CH_2_)_9_CH_3_), 1.26–1.40 (m, 36H, 2 x CH_2_CH_2_(CH_2_)_9_CH_3_), 0.93 (t, *J* = 7.0 Hz, 6H, 2 x CH_2_(CH_2_)_9_CH_3_); ^13^C NMR (CDCl_3_, 75.6 MHz): δ 162.8, 169.9, 191.7 (6 x C = O), 121.6, 134.9, 136.9 (6 x ArCH), 120.6, 134.2, 139.9 (6 x ArC), 40.0 (2 x NHCH_2_), 39.6 (ArCH_2_Ar), 25.2 (2 x COCH_3_), 22.7, 26.9, 29.2, 29.2, 29.4, 29.5, 29.6, 29.6, 31.3 (2 x CH_2_(CH_2_)_10_CH_3_), 14.1 (2 x CH_2_(CH_2_)_10_CH_3_); HRMS (ESI) *m*/*z* [M + Na]^+^calcd for C_45_H_68_N_4_NaO_6_: 761.5211; found: 761.5207.

*N,N’-(4,4’-bis-{methyl-2-[2-(2-acetamidophenyl)-2-oxoacetamido]acetate}) methane* (**21**). A mixture of the glycine methyl ester hydrochloride (0.24 g, 1.9 mmol) and saturated sodium hydrogen carbonate solution (3 mL) in water (7 mL) was added to a stirred solution of compound **7** (0.15 g, 0.38 mmol)) in dichloromethane (20 mL) was. The reaction mixture was stirred at room temperature for 24 h. The organic layer was diluted with dichloromethane (20 mL) and extracted in aqueous hydrochloric acid (0.5 M, 30 mL) and water (2 × 30 mL). The combined organic extracts were dried over anhydrous sodium sulfate and concentrated under vacuum. The crude compound was purified by gravity column chromatography (dichloromethane-ethyl acetate = 4:1) to afford the title compound as a yellow solid (0.120 g, 55%); mp 204–206 °C; IR (KBr): υ_max_ 3279, 1750, 1698, 1666, 1651, 1592, 1519, 1412, 1367, 1326, 1293, 1234, 1217, 1177, 1005, 688 cm**^−^**^1^; ^1^H NMR (CDCl_3_, 300 MHz): δ 10.49 (bs, 2H, 2 x NH) (disappears on D_2_O exchange), 9.07 (d, *J* = 11.2 Hz, 2H, ArH), 7.74 (s, 2H, ArH), 7.60 (d, *J* = 1.5 Hz, 2H, 2 x α-NH Gly) (disappears on D_2_O exchange), 7.49 (dd, *J* = 2.0, 8.5 Hz, 2H, ArH), 3.97 (d, *J* = 5.7 Hz, 4H, 2 x NHCH_2_COOCH_3_), 3.91 (s, 2H, ArCH_2_Ar), 3.67 (s, 6H, 2 x COOCH_3_), 2.05 (s, 6H, 2 x COCH_3_); ^13^C NMR (CDCl_3_, 75.6 MHz): δ 189.8 (COCH_3_), 169.5 (COCONH), 168.9 (COCONH), 163.2 (COOCH_3_), 149.8 (ArC), 138.6 (ArC), 127.1 (ArC), 123.5 (ArC), 122.6 (ArC), 119.6 (ArC), 53.1 (COOCH_3_), 40.9 (NHCH_2_COOCH_3_), 38.9 (ArCH_2_Ar), 24.9 (OCH_3_); HRMS (+ESI) *m*/*z* [M + Na]^+^ calcd for C_27_H_28_N_4_NaO_10_: 591.1703; found: 591.1692.

*N,N’-(4,4’-bis-{methyl-2-[2-(2-acetamidophenyl)-2-oxoacetamido]-4-methylpentanoate})methane* (**22**). Compound **22** was prepared by the same method as compound **21** from compound **7** (0.15 g, 0.38 mmol) and l-leucine methyl ester hydrochloride (0.30 g, 1.9 mmol) as a yellow solid (0.149 g, 57%); mp 184–186 °C; IR (KBr): υ_max_ 3328, 2958, 1747, 1648, 1592, 1522, 1438, 1415, 1370, 1298, 1253, 1231, 1176 cm**^−^**^1^; ^1^H NMR (CDCl_3_, 300 MHz): δ 10.84 (bs, 2H, 2 x NH) (disappears on D_2_O exchange), 8.53 (d, *J* = 8.8 Hz, 2H, ArH), 8.01 (d, *J* = 1.9 Hz, 2H, 2 x α-NH Leu) (disappears on D_2_O exchange), 7.35 (dd, *J* = 2.1, 8.7 Hz, 2H, ArH), 7.25 (d, *J* = 8.4 Hz, 2H, ArH), 4.57–4.64 (m, 2H, 2 x NHCHCOOCH_3_), 3.91 (s, 2H, ArCH_2_Ar), 3.66 (s, 6H, 2 x COOCH_3_), 2.14 (s, 6H, 2 x COCH_3_), 1.56-1.69 (m, 4H, 2 x CH_2_CH(CH_3_)_2_), 1.18–1.20 (m, 2H, 2 x CH(CH_3_)_2_), 0.89 (dd, *J* = 2.6, 5.9 Hz, 12H, 2 x CH(CH_3_)_2_); ^13^C NMR (CDCl_3_, 75.6 MHz): δ 191.9 (COCH_3_), 172.9 (COCONH), 169.5 (COCONH), 163.3 (COOCH_3_), 141.1 (ArC), 137.7 (ArC), 134.8 (ArC), 134.6 (ArC), 121.4 (ArC), 119.0 (ArC), 52.9 (COOCH_3_), 51.3 (NHCHCOOCH_3_), 41.7 (CH_2_CH(CH_3_)_2_), 40.3 (ArCH_2_Ar), 25.8 (OCH_3_), 25.3 (CH(CH_3_)_2_), 23.0 (CH_3_), 22.2 (CH_3_); HRMS (+ESI) *m*/*z* [M + Na]^+^ calcd for C_35_H_44_N_4_NaO_10_: 703.2955; found: 703.2948.

*N,N’-(4,4’-bis-{methyl-2-[2-(2-acetamidophenyl)-2-oxoacetamido]-4-(methylthio)butanoate})methane* (**23**). Compound **23** was prepared by the same method as compound **21** from compound **7** (0.15 g, 0.38 mmol) and l-methionine methyl ester hydrochloride (0.38 g, 1.9 mmol) as a yellow solid (0.140 g, 51%); mp 172–174 °C; IR (KBr): υ_max_ 3292, 1746, 1695, 1663, 1642, 1589, 1523, 1433, 1412, 1294, 1250, 1219, 1173 cm**^−^**^1^; ^1^H NMR (CDCl_3_, 300 MHz): δ 10.80 (bs, 2H, 2 x NH) (disappears on D_2_O exchange), 8.52 (d, *J* = 8.5 Hz, 2H, ArH), 8.01 (d, *J* = 1.9 Hz, 2H, 2 x α-NH Met) (disappears on D_2_O exchange), 7.49 (d, *J* = 8.1Hz, 2H, ArH), 7.35 (dd, *J* = 2.0, 8.7 Hz, 2H, ArH), 4.68–4.77 (m, 2H, 2 x NHCHCOOCH_3_), 3.91 (s, 2H, ArCH_2_Ar), 3.70 (s, 6H, 2 x COOCH_3_), 2.47 (t, *J* = 7.2 Hz, 4H, 2 x CH_2_CH_2_S), 2.15 (s, 6H, 2 x COCH_3_), 2.03 (s, 6H, 2 x SCH_3_), 1.05 (t, *J* = 7.2 Hz, 4H, 2 x CH_2_CH_2_S); ^13^C NMR (CDCl_3_, 75.6 MHz): δ 191.6 (COCH_3_), 171.9 (COCONH), 169.6 (COCONH), 163.3 (COOCH_3_), 141.1 (ArC), 137.7 (ArC), 134.8 (ArC), 134.5 (ArC), 121.5 (ArC), 118.9 (ArC), 53.2 (COOCH_3_), 52.0 (NHCHCOOCH_3_), 40.3 (ArCH_2_Ar), 31.6 (CH_2_CH_2_S), 30.3 (CH_2_CH_2_S), 25.8 (OCH_3_), 15.9 (SCH_3_); HRMS (+ESI) *m*/*z* [M + Na]^+^ calcd for C_33_H_40_N_4_O_10_NaS_2_: 739.2084; found: 739.2071.

*N,N’-(4,4’-bis-{methyl-2-[2-(2-acetamidophenyl)-2-oxoacetamido]-3-methylbutanoate})methane* (**24**). Compound **24** was prepared by the same method as compound **21** from compound **7** (0.15 g, 0.38 mmol) and l-valine methyl ester hydrochloride (0.32 g, 1.9 mmol) as a yellow solid (0.201 g, 80%); mp 204–207 °C; IR (KBr): υ_max_ 3251, 2962, 1740, 1696, 1666, 1644, 1588, 1521, 1469, 1438, 1413, 1369, 1295, 1245, 1225, 1211, 1176, 1141 cm**^−^**^1^; ^1^H NMR (CDCl_3_, 300 MHz): δ 10.87 (bs, 2H, 2 x NH) (disappears on D_2_O exchange), 8.51 (d, *J* = 8.6 Hz, 2H, ArH), 7.93 (d, *J* = 1.9 Hz, 2H, 2 x α-NH Val) (disappears on D_2_O exchange), 7.39 (d, *J* = 8.8 Hz, 2H, ArH′), 7.32 (dd, *J* = 2.0, 8.8 Hz, 2H, ArH), 4.54 (dd, *J* = 4.9, 9.0 Hz, 2H, 2 x NHCHCOOCH_3_), 3.89 (s, 2H, ArCH_2_Ar), 3.67 (s, 6H, 2 x COOCH_3_), 2.18–2.22 (m, 2H, 2 x CH(CH_3_)_2_), 2.14 (s, 6H, 2 x COCH_3_), 0.89 (dd, *J* = 6.9, 12.7 Hz, 12H, 2 x CH(CH_3_)_2_); ^13^C NMR (CDCl_3_, 75.6 MHz): δ 192.3 (COCH_3_), 172.1 (COCONH), 169.6 (COCONH), 163.7 (COOCH_3_), 141.1 (ArC), 137.7 (ArC), 134.7 (ArC), 134.5 (ArC), 121.4 (ArC), 118.9 (ArC), 57.6 (NHCHCOOCH_3_), 52.8 (COOCH_3_), 40.3 (ArCH_2_Ar), 31.8 (CH(CH_3_)_2_), 25.8 (OCH_3_), 19.4 (2 x CH_3_), 18.0 (CH_3_); HRMS (+ESI) *m*/*z* [M + Na]^+^ calcd for C_33_H_40_N_4_NaO_10_: 675.2642; found: 675.2616.

*N,N’-(4,4’-bis-{methyl-2-[2-(2-acetamidophenyl)-2-oxoacetamido]-3-phenylpropanoate})methane* (**25**). Compound **25** was prepared by the same method as compound **21** from compound **7** (0.15 g, 0.38 mmol) and d-phenylalanine methyl ester hydrochloride (0.41 g, 1.9 mmol) as a yellow solid (0.081 g, 28%); mp 200–202 °C; $[\alpha {]}_{D}^{22}$ (c 0.1 in MeOH); IR (KBr): υ_max_ 3294, 1749, 1693, 1644, 1590, 1521, 1412, 1293, 1268, 1217, 1173, 701 cm**^−^**^1^; ^1^H NMR (CDCl_3_, 300 MHz): δ 10.79 (bs, 2H, 2 x NH) (disappears on D_2_O exchange), 8.50 (d, *J* = 9.0 Hz, 2H, ArH), 7.88 (s, 2H, 2 x α-NH Phe) (disappears on D_2_O exchange), 7.16–7.29 (d, 12H, ArH, ArH), 7.05 (d, *J* = 6.4 Hz, 2H, ArH), 4.86 (dd, *J* = 6.6, 14.8 Hz, 2H, 2 x NHCHCOOCH_3_), 3.84 (s, 2H, ArCH_2_Ar), 3.66 (s, 6H, 2 x COOCH_3_), 2.99-3.17 (m, 4H, 2 x CH_2_Ph), 2.11 (s, 6H, 2 x COCH_3_); ^13^C NMR (CDCl_3_, 75.6 MHz): δ 191.7 (COCH_3_), 171.5 (COCONH), 169.6 (COCONH), 163.0 (COOCH_3_), 141.1 (ArC), 137.6 (ArC), 135.7 (ArC), 134.7 (ArC), 134.5 (ArC), 129.6 (ArC), 129.1 (ArC), 127.8 (ArC), 121.4 (ArC), 118.8 (ArC), 53.7 (NHCHCOOCH_3_), 53.0 (COOCH_3_), 40.3 (ArCH_2_Ar), 38.3 (CH_2_Ph), 25.8 (OCH_3_); HRMS (+ESI) *m*/*z* [M + Na]^+^ calcd for C_41_H_40_N_4_NaO_10_: 771.2642; found: 771.2610.

*N,N’-(4,4’-bis-{methyl-2-[2-(2-acetamidophenyl)-2-oxoacetamido]-3-phenylpropanoate})methane* (**26**). Compound **26** was prepared from compound **7** (1.00 g, 2.56 mmol) and l-phenylalanine methyl ester hydrochloride (1.21 g, 5.64 mmol) in the presence of Et_3_N (1.04g, 10.2 mmol) as a yellow solid (0.78 mg, 48%); mp 201–202 °C; $[\alpha {]}_{D}^{23}$ (c 0.1 in MeOH)**;** IR υ_max_ 3291, 1742, 1690, 1642, 1594, 1523, 1418, 1289, 1272, 1212, 1171, 705 cm^−1^. ^1^H NMR (CDCl_3_, 400 MHz): δ 10.80 (bs, 2H, 2 x NH) (disappears on D_2_O exchange), 8.51 (d, *J* = 9.0 Hz, 2H, ArH), 7.89 (s, 2H, 2 x α-NH Phe) (disappears on D_2_O exchange), 7.14-7.30 (d, 12H, ArH, ArH), 7.06 (d, *J* = 6.4 Hz, 2H, ArH), 4.88 (dd, *J* = 6.6, 14.8 Hz, 2H, 2 x NHCHCOOCH_3_), 3.85 (s, 2H, ArCH_2_Ar), 3.67 (s, 6H, 2 x COOCH_3_), 2.98–3.18 (m, 4H, 2 x CH_2_Ph), 2.12 (s, 6H, 2 x COCH_3_); ^13^C NMR (CDCl_3_, 101 MHz): δ 191.8 (COCH_3_), 172.1 (COCONH), 169.6 (COCONH), 163.0 (COOCH_3_), 141.1 (ArC), 137.6 (ArC), 135.7 (ArC), 134.7 (ArC), 135.6 (ArC), 129.9 (ArC), 129.2 (ArC), 128.1 (ArC), 121.2 (ArC), 119.2 (ArC), 54.1 (NHCHCOOCH_3_), 53.1 (COOCH_3_), 40.2 (ArCH_2_Ar), 38.4 (CH_2_Ph), 25.9 (OCH_3_); HRMS (+ESI) *m*/*z* [M + Na]^+^ calcd for C_41_H_40_N_4_NaO_10_: 771.2642; found: 771.2612.

*N,N’-(4,4’-bis-{methyl-2-[2-(2-acetamidophenyl)-2-oxoacetamido]acetate})oxide* (**27**). Compound **27** was prepared by the same method as compound **21** from compound **8** (0.15 g, 0.38 mmol) and glycine methyl ester hydrochloride (0.24 g, 1.9 mmol) as a yellow solid (0.061 g, 28%); mp 180–182 °C; IR (KBr): υ_max_ 3354, 3293, 2923, 1747, 1686, 1658, 1588, 1512, 1438, 1408, 1373, 1327, 1286, 1262, 1219, 1183, 1011 cm^−1^; ^1^H NMR (CDCl_3_, 300 MHz): δ 10.69 (bs, 2H, 2 x NH) (disappears on D_2_O exchange), 8.60 (d, *J* = 9.3 Hz, 2H, ArH), 7.93 (d, *J* = 3.0 Hz, 2H, 2 x α-NH Gly) (disappears on D_2_O exchange), 7.36 (t, *J* = 5.3 Hz, 2H, ArH), 7.49 (dd, *J* = 3.0, 9.2 Hz, 2H, ArH), 4.05 (d, *J* = 5.5 Hz, 4H, 2 x NHCH_2_COOCH_3_), 3.68 (s, 6H, 2 x COOCH_3_), 2.17 (s, 6H, 2 x COCH_3_); ^13^C NMR (CDCl_3_, 75.6 MHz): δ 190.9 (COCH_3_), 169.8 (COCONH), 169.5 (COCONH), 163.2 (COOCH_3_), 151.6 (ArC), 138.5 (ArC), 127.8 (ArC), 123.5 (ArC), 123.1 (ArC), 119.9 (ArC), 53.1 (COOCH_3_), 41.5 (NHCH_2_COOCH_3_), 25.8 (OCH_3_). HRMS (+ESI) *m*/*z* [M + Na]^+^ calcd for C_26_H_26_N_4_NaO_11_: 593.1496; found: 593.1472.

*N,N’-(4,4’-bis-{methyl-2-[2-(2-acetamidophenyl)-2-oxoacetamido]-4-methylpentanoate})oxide* (**28**). Compound **28** was prepared by the same method as compound **21** from compound **8** (0.15 g, 0.38 mmol) and l-leucine methyl ester hydrochloride (0.30 g, 1.9 mmol) as a yellow solid (0.068 g, 26%); mp 106–108 °C; IR (KBr): υ_max_ 3313, 2958, 1748, 1655, 1589, 1519, 1438, 1410, 1370, 1263, 1217, 1182, 1162, 1013, 830, 698 cm^−1^; ^1^H NMR (CDCl_3_, 300 MHz): δ 10.82 (bs, 2H, 2 x NH) (disappears on D_2_O exchange), 8.64 (d, *J* = 9.3 Hz, 2H, ArH), 7.83 (d, *J* = 2.7 Hz, 2H, 2 x α-NH Leu) (disappears on D_2_O exchange), 7.41 (d, *J* = 8.5 Hz, 2H, ArH), 7.28 (dd, *J* = 2.9, 9.2 Hz, 2H, ArH), 4.60–4.67 (m, 2H, 2 x NHCHCOOCH_3_), 3.67 (s, 6H, 2 x COOCH_3_), 2.21 (s, 6H, 2 x COCH_3_), 1.57–1.72 (m, 6H, 2 x CH_2_CH(CH_3_)_2_), 0.93 (d, *J* = 5.9 Hz, 12H, CH(CH_3_)_2_); ^13^C NMR (CDCl_3_, 75.6 MHz): δ 191.3 (COCH_3_), 172.7 (COCONH), 169.0 (COCONH), 162.9 (COOCH_3_), 151.1 (ArC), 138.0 (ArC), 127.3 (ArC), 122.9 (ArC), 122.6 (ArC), 119.4 (ArC), 52.5 (COOCH_3_), 50.8 (NHCHCOOCH_3_), 41.3 (CH_2_CH(CH_3_)_2_), 25.3 (OCH_3_), 24.9 (CH(CH_3_)_2_), 22.8 (CH_3_), 21.8 (CH_3_); HRMS (+ESI) *m*/*z* [M + Na]^+^ calcd for C_34_H_42_N_4_NaO_11_: 705.2748; found: 705.2720.

*N,N’-(4,4’-bis-{methyl-2-[2-(2-acetamidophenyl)-2-oxoacetamido]-4-(methylthio)butanoate})oxide* (**29**). Compound **29** was prepared by the same method as compound **21** from compound **8** (0.15 g, 0.38 mmol) and l-methionine methyl ester hydrochloride (0.38 g, 1.9 mmol) as a yellow solid (0.206 g, 75%); mp 102–104 °C; IR (KBr): υ_max_ 3274, 1743, 1658, 1588, 1519, 1489, 1410, 1370, 1271, 1218, 1183, 1162 cm^−1^; ^1^H NMR (CDCl_3_, 300 MHz): δ 10.77 (bs, 2H, 2 x NH) (disappears on D_2_O exchange), 8.63 (d, *J* = 9.3 Hz, 2H, ArH), 7.87 (d, *J* = 2.9 Hz, 2H, 2 x α-NH Met) (disappears on D_2_O exchange), 7.65 (d, *J* = 8.1 Hz, 2H, ArH), 7.29 (dd, *J* = 2.9, 9.2 Hz, 2H, ArH), 4.74 (dd, *J* = 8.8, 12.9 Hz, 2H, 2 x NHCHCOOCH_3_), 3.72 (s, 6H, 2 x COOCH_3_), 2.49–2.56 (m, 4H, 2 x CH_2_CH_2_S), 2.21 (s, 6H, 2 x COCH_3_), 2.07 (s, 6H, 2 x SCH_3_), 1.03 (t, *J* = 6.8 Hz, 4H, 2 x CH_2_CH_2_S); ^13^C NMR (CDCl_3_, 75.6 MHz): δ 190.8 (COCH_3_), 171.6 (COCONH), 169.1 (COCONH), 162.7 (COOCH_3_), 151.1 (ArC), 138.0 (ArC), 127.3(ArC), 122.9 (ArC), 122.6 (ArC), 119.5 (ArC), 52.8 (COOCH_3_), 51.5 (NHCHCOOCH_3_), 31.1 (CH_2_CH_2_S), 29.9 (CH_2_CH_2_S), 25.3 (OCH_3_), 15.5 (SCH_3_); HRMS (+ESI) *m*/*z* [M + Na]^+^ calcd for C_32_H_38_N_4_O_11_NaS_2_: 741.1876; found: 741.1854.

*N,N’-(4,4’-bis-{methyl-2-[2-(2-acetamidophenyl)-2-oxoacetamido]-3-methylbutanoate})oxide* (**30**). Compound **30** was prepared by the same method as compound **21** from compound **8** (0.15 g, 0.38 mmol) and l-valine methyl ester hydrochloride (0.32 g, 1.9 mmol) as a yellow solid (0.115 g, 46%); mp 99–101 °C; IR (KBr): υ_max_ 3215, 2992, 1743, 1701, 1660, 1591, 1518, 1485, 1417, 1370, 1289, 1256, 1215, 1210, 1016, 920, 836, 741 cm^−1^; ^1^H NMR (CDCl_3_, 300 MHz): δ 10.80 (bs, 2H, 2 x NH) (disappears on D_2_O exchange), 8.61 (d, *J* = 9.2 Hz, 2H, ArH), 7.70 (d, *J* = 2.8 Hz, 2H, 2 x α-NH Val) (disappears on D_2_O exchange), 7.47 (d, *J* = 9.1 Hz, 2H, ArH), 7.24 (dd, *J* = 2.8, 9.2 Hz, 2H, ArH), 4.54 (dd, *J* = 4.8, 9.1 Hz, 2H, 2 x NHCHCOOCH_3_), 3.66 (s, 6H, 2 x COOCH_3_), 2.16 (s, 6H, 2 x COCH_3_), 1.18–1.20 (m, 2H, 2 x CH(CH_3_)_2_), 0.88 (dd, *J* = 6.9, 15.3 Hz, 12H, 2 x CH(CH_3_)_2_); ^13^C NMR (CDCl_3_, 75.6 MHz): δ 192.0 (COCH_3_), 172.4 (COCONH), 169.4 (COCONH), 163.7 (COOCH_3_), 151.5 (ArC), 138.5 (ArC), 127.8 (ArC), 123.1 (ArC), 123.0 (ArC), 119.7 (ArC), 57.4 (NHCHCOOCH_3_), 52.8 (COOCH_3_), 31.8 (CH(CH_3_)_2_), 25.7 (OCH_3_), 18.0 (CH_3_), 19.4 (CH_3_); HRMS (+ESI) *m*/*z* [M + Na]^+^ calcd for C_33_H_40_N_4_NaO_10_: 677.2435; found: 677.2419.

*N,N’-(4,4’-bis-{methyl-2-[2-(2-acetamidophenyl)-2-oxoacetamido]-3-phenylpropanoate})oxide* (**31**). Compound **31** was prepared by the same method as compound **21** from compound **8** (0.15 g, 0.38 mmol) and d-phenylalanine methyl ester hydrochloride (0.41 g, 1.9 mmol) as a yellow solid (0.196 g, 68%); mp 200–202 °C; IR (KBr): υ_max_ 3298, 1745, 1669, 1587, 1518, 1496, 1439, 1410, 1370, 1267, 1218, 1180, 702 cm^−1^; ^1^H NMR (CDCl_3_, 300 MHz): δ 10.78 (bs, 2H, 2 x NH) (disappears on D_2_O exchange), 8.67 (d, *J* = 9.3 Hz, 2H, ArH), 7.79 (d, *J* = 2.9 Hz, 2H, 2 x α-NH Phe) (disappears on D_2_O exchange), 7.22-7.31 (m, 12H, ArH), 7.11 (dd, *J* = 1.7, 8.0 Hz, 2H, ArH), 4.86–4.93 (m, 2H, 2 x NHCHCOOCH_3_), 3.70 (s, 6H, 2 x COOCH_3_), 3.00–3.19 (m, 4H, 2 x CH_2_Ph), 2.21 (s, 6H, 2 x COCH_3_); ^13^C NMR (CDCl_3_, 75.6 MHz): δ 190.9 (COCH_3_), 171.3 (COCONH), 169.0 (COCONH), 162.3 (COOCH_3_), 151.1 (ArC), 138.1 (ArC), 135.2 (ArC), 129.2 (ArC), 128.8 (ArC), 127.4 (ArC), 127.3 (ArC), 123.0 (ArC), 122.6 (ArC), 119.3 (ArC), 53.2 (NHCHCOOCH_3_), 52.6 (COOCH_3_), 37.9 (2 x CH_2_Ph), 25.4 (OCH_3_); HRMS (+ESI) *m*/*z* [M + Na]^+^ calcd for C_40_H_38_N_4_NaO_11_: 773.2435; found: 773.2419.

*N,N’-(4,4’-bis-{methyl-2-[2-(2-acetamidophenyl)-2-oxoacetamido]-3-phenylpropanoate})*oxide (**32**). Compound **32** was prepared by the same method as compound **21** from compound **8** (1.00 g, 2.55 mmol), l-phenylalanine methyl ester hydrochloride (1.21 g, 5.61 mmol) and Et_3_N (1.03 g, 10.20 mmol) as a yellow solid (1.50 g, 78%); mp 201–202 °C; [α]_D_ -12 (c 0.1 in MeOH); IR: υ_max_ 3292, 1743, 1671, 1582, 1520, 1494, 1440, 1411, 1371, 1265, 1211, 1178, 701 cm^−1^; ^1^H NMR (CDCl_3_, 400 MHz): δ 10.74 (bs, 2H, 2 x NH) (disappears on D_2_O exchange), 8.65 (d, *J* = 9.2 Hz, 2H, ArH), 7.80 (d, *J* = 3.0 Hz, 2H, 2 x α-NH Phe) (disappears on D_2_O exchange), 7.21–7.34 (m, 12H, ArH), 7.10 (dd, *J* = 1.8, 8.0 Hz, 2H, ArH), 4.87–4.91 (m, 2H, 2 x NHCHCOOCH_3_), 3.71 (s, 6H, 2 x COOCH_3_), 3.00–3.20 (m, 4H, 2 x CH_2_Ph), 2.22 (s, 6H, 2 x COCH_3_); ^13^C NMR (CDCl_3_, 101 MHz): δ 190.8 (COCH_3_), 171.2 (COCONH), 168.5 (COCONH), 162.0 (COOCH_3_), 151.2 (ArC), 137.8 (ArC), 135.1 (ArC), 129.1 (ArC), 128.7 (ArC), 127.3 (ArC), 127.1 (ArC), 123.0 (ArC), 122.5 (ArC), 119.0 (ArC), 53.1 (NHCHCOOCH_3_), 52.5 (COOCH_3_), 37.7 (2 x CH_2_Ph), 25.3 (OCH_3_); HRMS (+ESI) *m*/*z* [M + Na]^+^ calcd for C_40_H_38_N_4_NaO_11_: 773.2435; found: 773.2428.

### Single-Crystal X-Ray Diffraction

The X-ray diffraction measurements for compounds **22**–**24** were carried out at MX1 and MX2 beamlines at the Australian Synchrotron Facility, Melbourne. The procedure for diffraction intensity measurements on both beamlines was similar. The crystal was mounted on the goniometer using a cryo loop for diffraction measurements, and it was coated with paraffin oil and then quickly transferred to the cold stream using cryo stream attachment. Data were collected using Si<111> monochromated synchrotron X-ray radiation (λ = 0.71023 Å) at 100(2) K and were corrected for Lorentz and polarization effects using the XDS software [23]. The structure was solved by direct methods and the full-matrix least-squares refinements were carried out using SHELXL [26]. X-ray crystallographic information files (CIF) for the structures **22**–**24** are CCDC 1505262, 1,505, 263 and 1956306. A copy of the data can be obtained free of charge from CCDC, 12 Union Road, Cambridge CB2 1EZ, UK or email: deposit@ccdc.cam.ac.uk.

## 4. Conclusions

The synthesis of a library of bis-glyoxylamide peptidomimetics was achieved by nucleophilic ring-opening reactions of bis-*N*-acetylisatins linked at C5 by a methylene or oxygen bridge with alcohols, amines, and amino acid methyl ester hydrochlorides. The bis-isatins were prepared using a modified Sandmeyer isonitrosoacetanilide isatin synthesis using 1,4-dioxane instead of hydrochloric acid as the reaction solvent. Amines were the most reactive reagents for ring-opening of the bis-*N*-acetylisatins and the products were obtained in high yields. The addition of sodium bicarbonate was needed for ring-opening of bis-*N*-acetylisatins with amino acid methyl ester hydrochlorides and the reactions were complete in 24 h. The alcohols were slow to react and heating the reaction mixture at reflux was required for ethanol. The biological activity of these compounds is currently under investigation.

## Figures and Tables

**Table 1:** Bis-glyoxylamide peptidomimetics **21–32**.

No	Product	X	Amino Acid Methyl Esters	Yields (%)
1	**21**	CH_2_	Gly OMe	55
2	**22**	CH_2_	l-Leu OMe	57
3	**23**	CH_2_	l-Met OMe	51
4	**24**	CH_2_	l-Val OMe	80
5	**25**	CH_2_	d-Phe OMe	28
6	**26**	CH_2_	l-Phe OMe	48
7	**27**	O	Gly OMe	28
8	**28**	O	l-Leu OMe	26
9	**29**	O	l-Met OMe	76
10	**30**	O	l-Val OMe	46
11	**31**	O	d-Phe OMe	68
12	**32**	O	l-Phe OMe	78

## Supplementary Materials

The following are available online at https://www.mdpi.com/1420-3049/24/23/4343/s1, ^1^H and ^13^C NMR spectra for the synthesized compounds.
