# Supplementary material for: Synthesis of Bis-Glyoxylamide Peptidomimetics Derived from Bis-N-acetylisatins Linked at C5 by a Methylene or Oxygen Bridge
Source: Molecules. 2019 Nov 27;24(23):4343. doi: 10.3390/molecules24234343 (PMC6930608; doi:10.3390/molecules24234343)

Supervisor Kumar-1 bisisatin5

COMPOUND 3

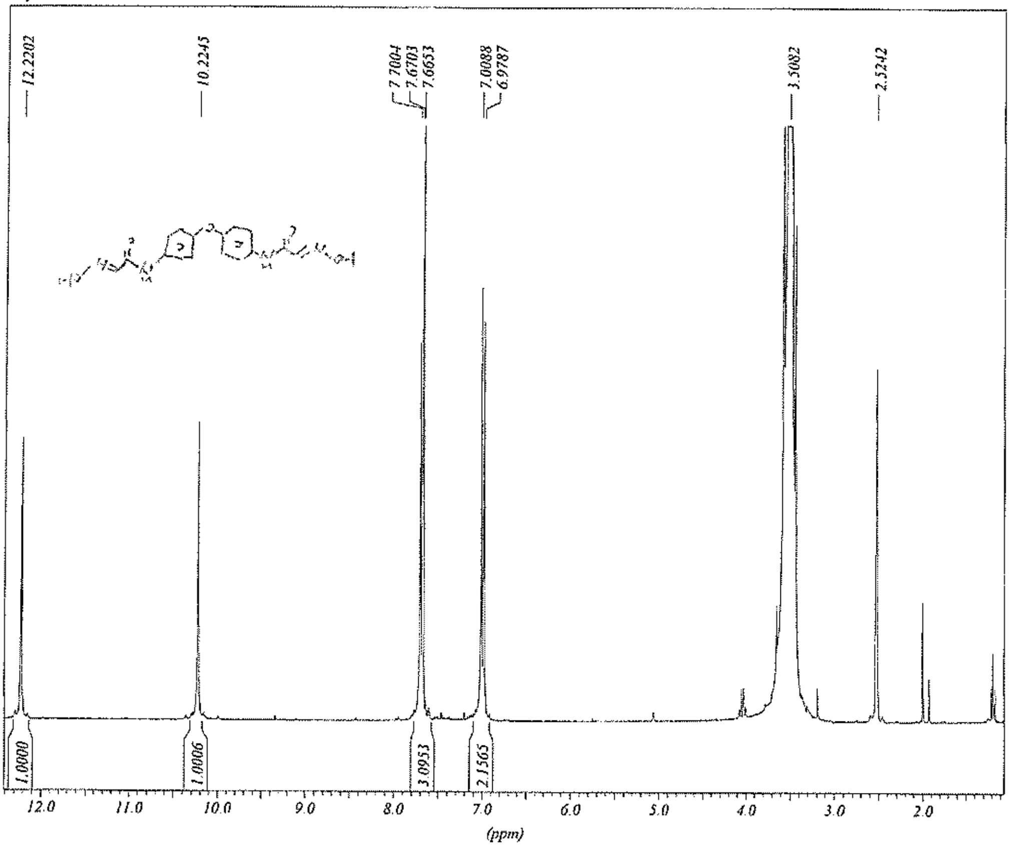

*** Current Data Parameters ***

NAME	0RP9RM~A
EXPNO	10
PROCNO	1

*** Acquisition Parameters ***

DATE_t	21:50:38
DATE_d	Aug 24 2009
DS	2
LOCNUC	2H
NS	16
O1	1853.67 Hz
O2	1853.67 Hz
RG	114.0000000
SFO1	300.1718537 MHz
SOLVENT	DMSO
SW	20.5644 ppm
TD	32768
TE	300.2 K

*** Processing Parameters ***

LB	0.30 Hz
PC	1.00
SF	300.1700000 MHz
SI	16384

*** 1D NMR Plot Parameters ***

SR	-0.00 Hz
SOLVENT	?

Supervisor Kumarbisisatin5

COMPOUND 3

BRUKER

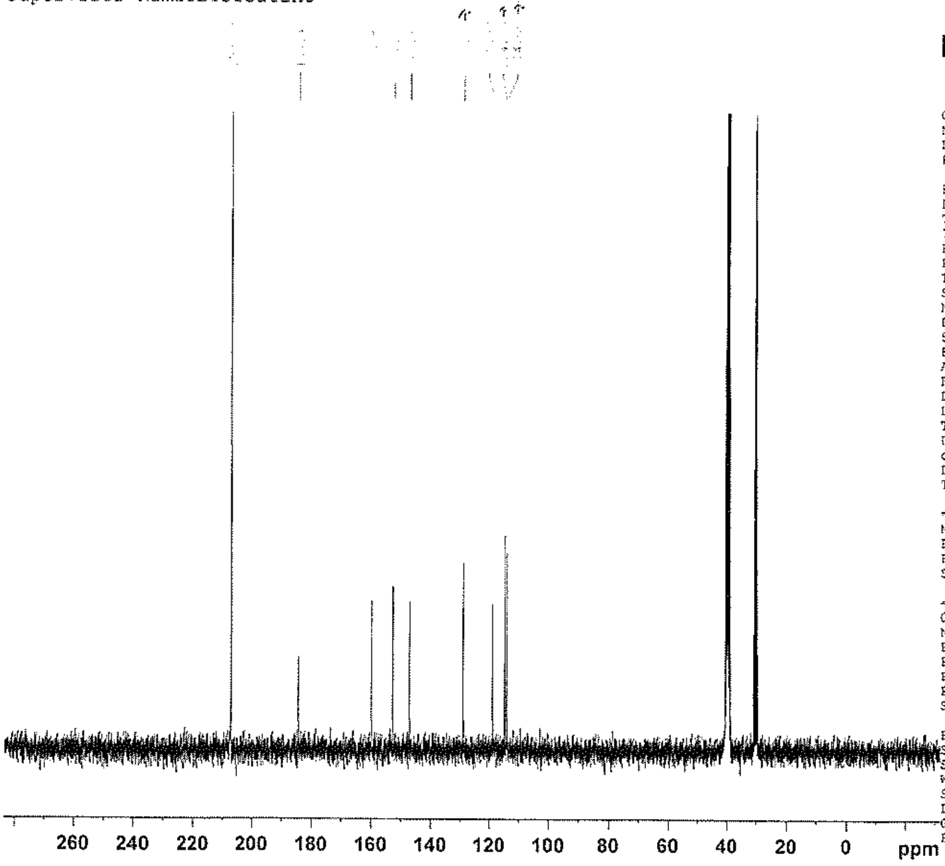

Current Data Parameters

NAME	101208-zrn
EXPNO	21
PROCNO	1

F2 - Acquisition Parameters

Date_	20101209
Time	6.07
INSTRUM	dpx1
PROBHD	5 mm QNP 1H/13
PULPROG	zgpg30
TD	65536
SOLVENT	DMSO
NS	1024
DS	2
SWH	23809.523 Hz
FIDRES	0.363304 Hz
AQ	1.3763061 sec
RG	16384
DW	21.000 usec
DE	25.00 usec
TE	293.6 K
D1	2.00000000 sec
d11	0.03000000 sec
DELTA	1.89999998 sec
TD0	1

======== CHANNEL f1 ========

NUC1	13C
P1	7.50 usec
PL1	-3.00 dB
SFO1	75.4873094 MHz

======== CHANNEL f2 ========

CPDPRG2	waltz16
NUC2	1H
PCPD2	105.00 usec
PL2	-3.00 dB
PL12	17.34 dB
PL13	23.60 dB
SFO2	300.1715008 MHz

F2 - Processing parameters

SI	32768
SF	75.4778155 MHz
WDW	EM
SSB	0
LB	1.00 Hz
GB	0
PC	1.40

Supervisor Kumar-1 bisisatin5

COMPOUND 4

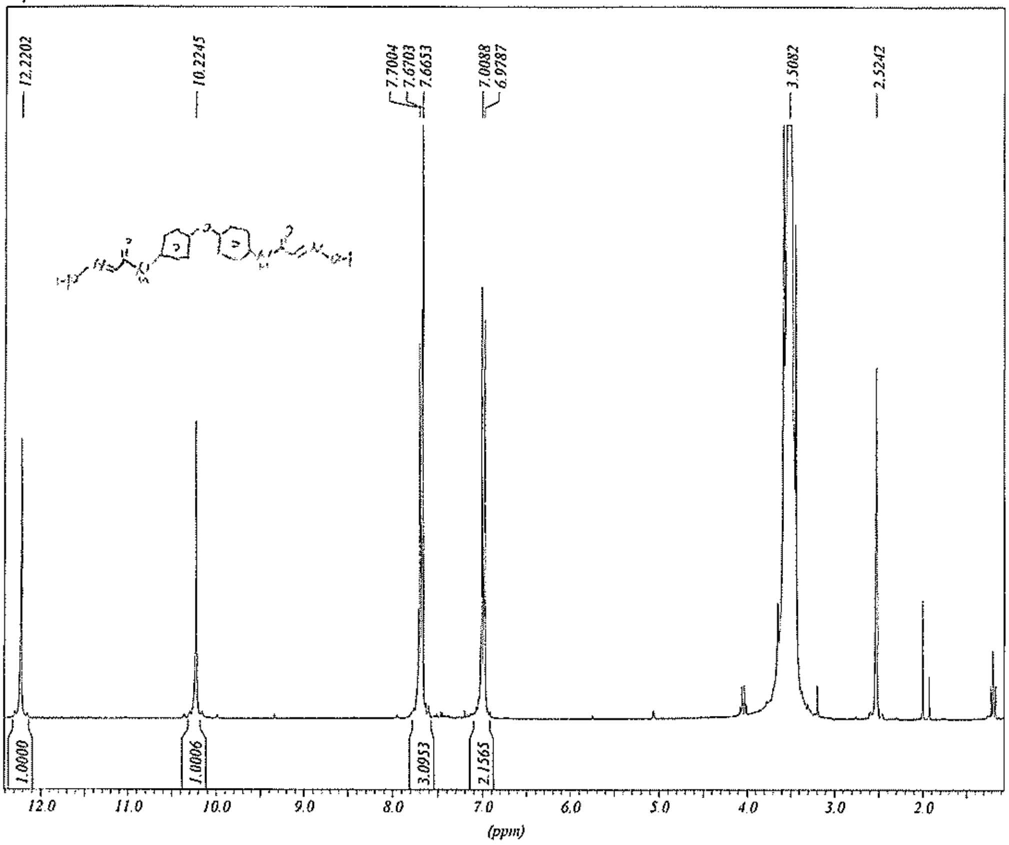

*** Current Data Parameters ***

NAME	:	0RP9RM-A
EXPNO	:	10
PROCNO	:	1

*** Acquisition Parameters ***

DATE_t	:	21:50:38
DATE_d	:	Aug 24 2009
DS	:	2
LOCNUC	:	2H
NS	:	16
O1	:	1853.67 Hz
O2	:	1853.67 Hz
RG	:	114.0000000
SFO1	:	300.1718537 MHz
SOLVENT	:	DMSO
SW	:	20.5644 ppm
TD	:	32768
TE	:	300.2 K

*** Processing Parameters ***

LB	:	0.30 Hz
PC	:	1.00
SF	:	300.1700000 MHz
SI	:	16384

*** 1D NMR Plot Parameters ***

SR	:	-0.00 Hz
SOLVENT	:	?

Supervisor Kumarbisisatin5

COMPOUND 4

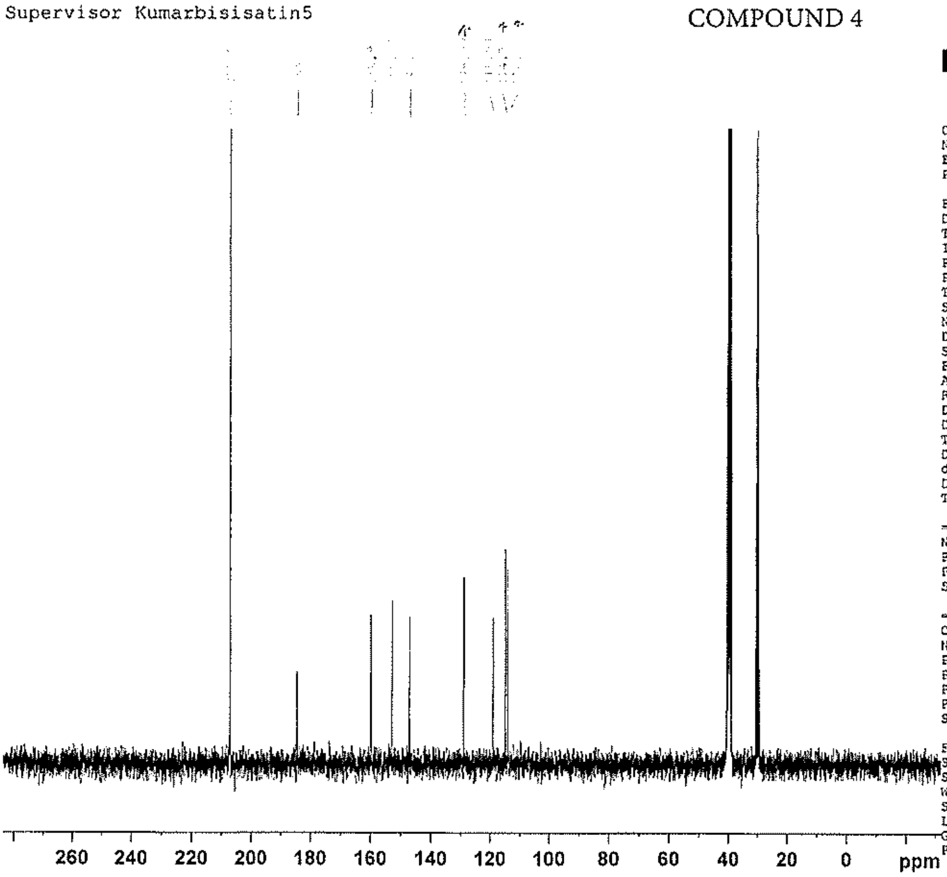

BRUKER

Current Data Parameters

NAME	101208-zrn
EXPNO	21
PROCNO	1

F2 - Acquisition Parameters

Date_	20101209
Time	6.07
INSTRUM	dpx1
PROBHD	5 mm QNP 1H/13
PULPROG	zgpg30
TD	65536
SOLVENT	DMSO
NS	1024
DS	2
SWH	23809.523 Hz
FIDRES	0.363304 Hz
AQ	1.3763061 sec
RG	16384
DW	21.000 usec
DE	25.00 usec
TE	293.6 K
D1	2.00000000 sec
d11	0.03000000 sec
DELTA	1.89999998 sec
TD0	1

======== CHANNEL f1 ========

NUC1	13C
P1	7.50 usec
PL1	-3.00 dB
SFO1	75.4873094 MHz

======== CHANNEL f2 ========

CPDPRG2	waltz16
NUC2	1H
PCPD2	105.00 usec
PL2	-3.00 dB
PL12	17.34 dB
PL13	23.60 dB
SFO2	300.1715008 MHz

F2 - Processing parameters

SI	32768
SF	75.4778155 MHz
WDW	EM
SSB	0
LB	1.00 Hz
GB	0
PC	1.40

COMPOUND 5

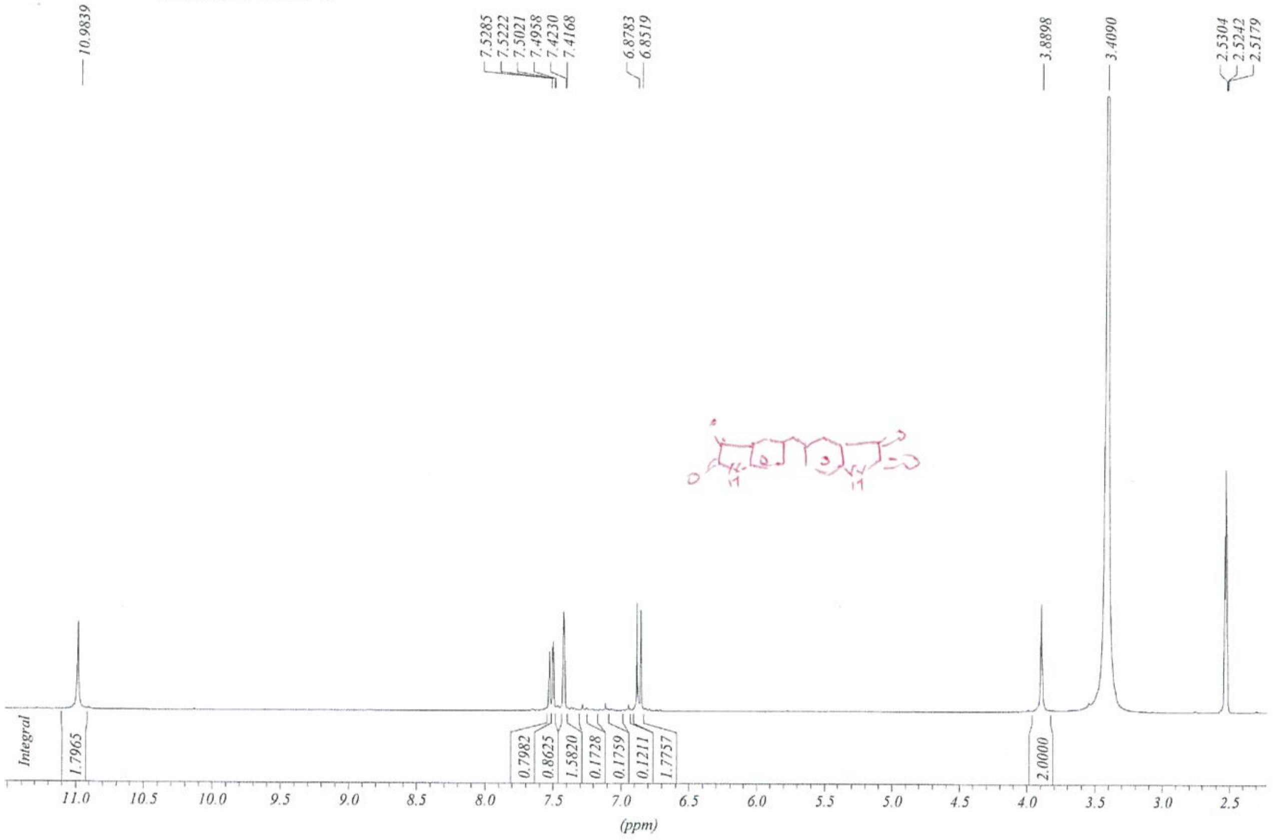

8

Supervisor Kumarbisisatin4

COMPOUND 5

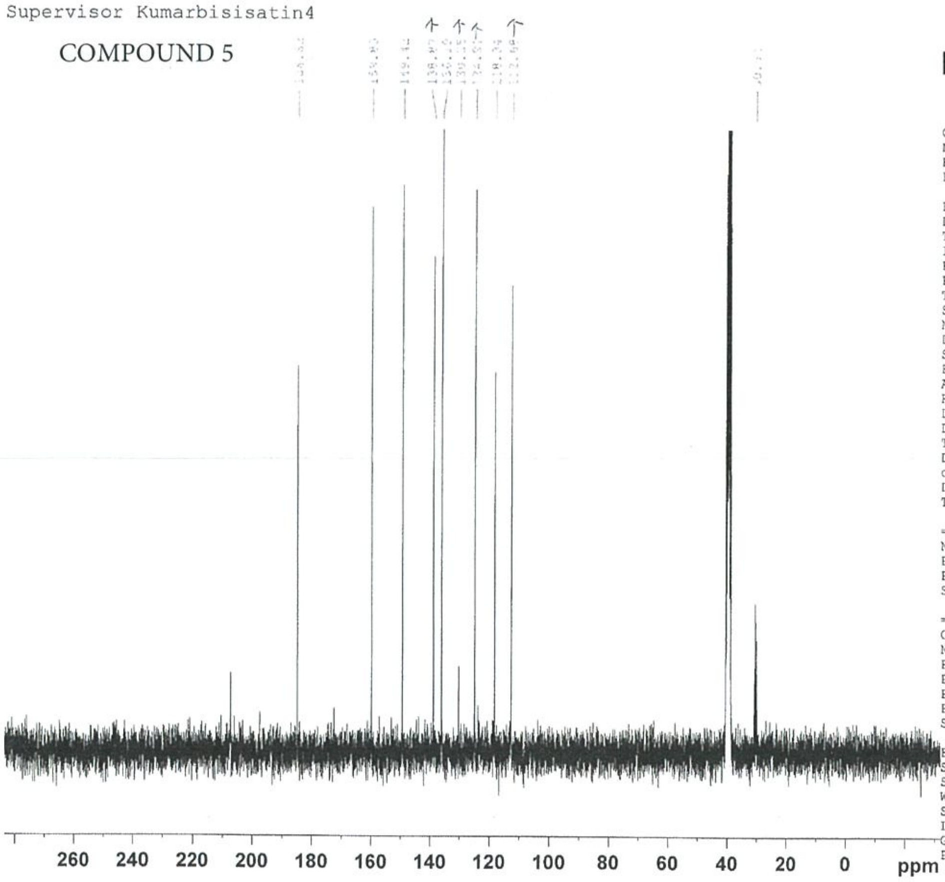

Current Data Parameters
NAME 101208-zrn
EXPNO 11
PROCNO 1

F2 - Acquisition Parameters
Date_ 20101209
Time 4.46
INSTRUM dpx1
PROBHD 5 mm QNP 1H/13
PULPROG zgpg30
TD 65536
SOLVENT DMSO
NS 1024
DS 2
SWH 23809.523 Hz
FIDRES 0.363304 Hz
AQ 1.3763061 sec
RG 14596.5
DW 21.000 usec
DE 25.00 usec
TE 293.6 K
D1 2.00000000 sec
d11 0.03000000 sec
DELTA 1.89999998 sec
TD0 1

======== CHANNEL f1 ========
NUC1 13C
P1 7.50 usec
PL1 -3.00 dB
SFO1 75.4873094 MHz

======== CHANNEL f2 ========
CPDPRG2 waltz16
NUC2 1H
PCPD2 105.00 usec
PL2 -3.00 dB
PL12 17.34 dB
PL13 23.60 dB
SFO2 300.1715008 MHz

F2 - Processing parameters
SI 32768
SF 75.4778155 MHz
WDW EM
SSB 0
LB 1.00 Hz
GB 0
PC 1.40

8

Supervisor Kumarbisisatin 5

COMPOUND 6

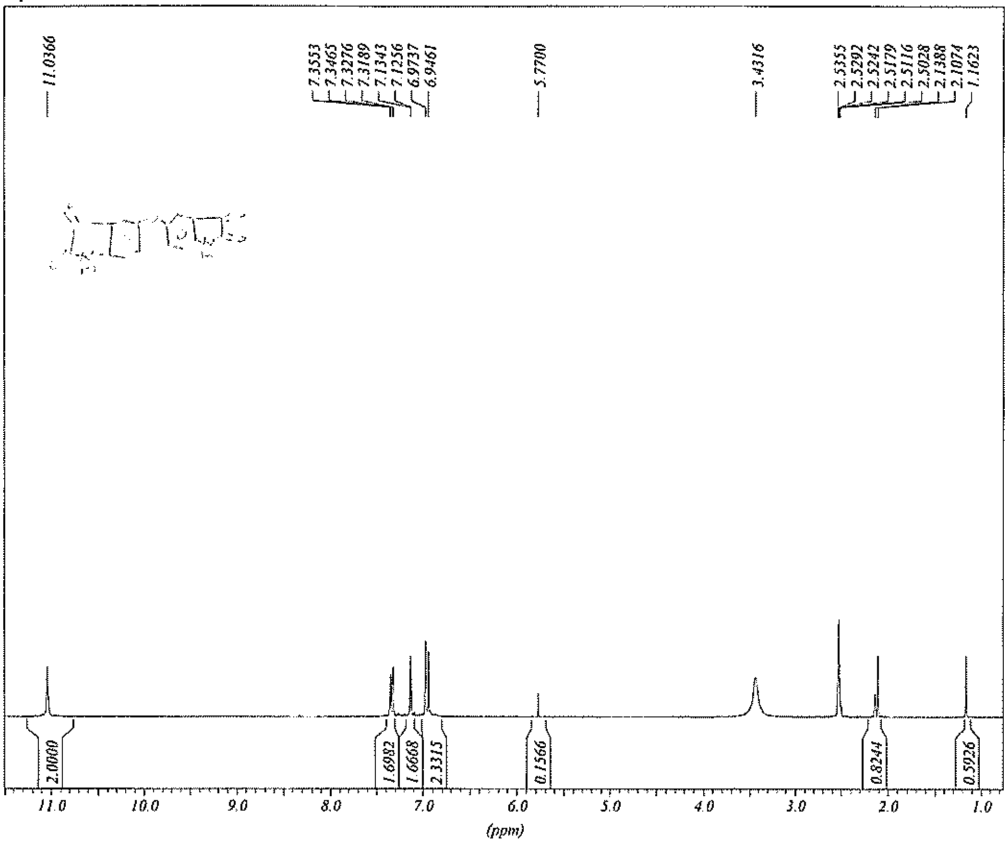

*** *Current Data Parameters* ***

NAME	:	09PYBK~L
EXPNO	:	60
PROCNO	:	1

*** *Acquisition Parameters* ***

DATE_t	:	08:25:11
DATE_d	:	Sep 01 2009
DS	:	2
LOCNUC	:	2H
NS	:	16
O1	:	1853.67 Hz
O2	:	1853.67 Hz
RG	:	645.0999756
SFO1	:	300.1718537 MHz
SOLVENT	:	DMSO
SW	:	20.5644 ppm
TD	:	32768
TE	:	300.2 K

*** *Processing Parameters* ***

LB	:	0.30 Hz
PC	:	1.00
SF	:	300.1700000 MHz
SI	:	16384

*** *1D NMR Plot Parameters* ***

SR	:	-0.00 Hz
SOLVENT	:	?

12

Supervisor Kumaracyl-bisisatin5

COMPOUND 6

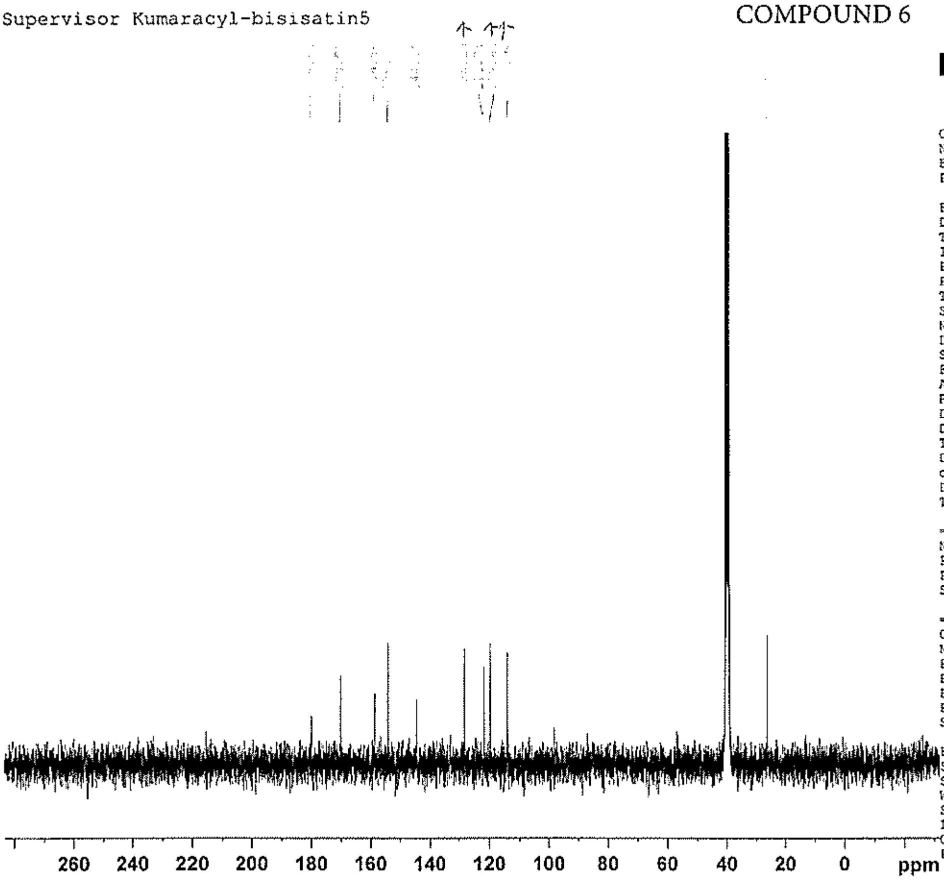

BRUKER

Current Data Parameters	
NAME	101208-zrn
EXPNO	31
PROCNO	1

F2 - Acquisition Parameters	
Date_	20101209
Time	7.29
INSTRUM	dpx1
PROBHD	5 mm QNP 1H/13
PULPROG	zgpg30
TD	65536
SOLVENT	DMSO
NS	1024
DS	2
SWH	23809.523 Hz
FIDRES	0.363304 Hz
AQ	1.3763061 sec
RG	16384
DW	21.000 usec
DE	25.00 usec
TE	293.6 K
D1	2.00000000 sec
d11	0.03000000 sec
DELTA	1.89999998 sec
TD0	1

======== CHANNEL f1 ========	
NUC1	13C
P1	7.50 usec
PL1	-3.00 dB
SFO1	75.4873094 MHz

======== CHANNEL f2 ========	
CPDPRG2	waltz16
NUC2	1H
PCPD2	105.00 usec
PL2	-3.00 dB
PL12	17.34 dB
PL13	23.60 dB
SFO2	300.1715008 MHz

F2 - Processing parameters	
SI	32768
SF	75.4778155 MHz
WDW	EM
SSB	0
LB	1.00 Hz
GB	0
PC	1.40

8

Supervisor Kumaracyl-bisisatin-4

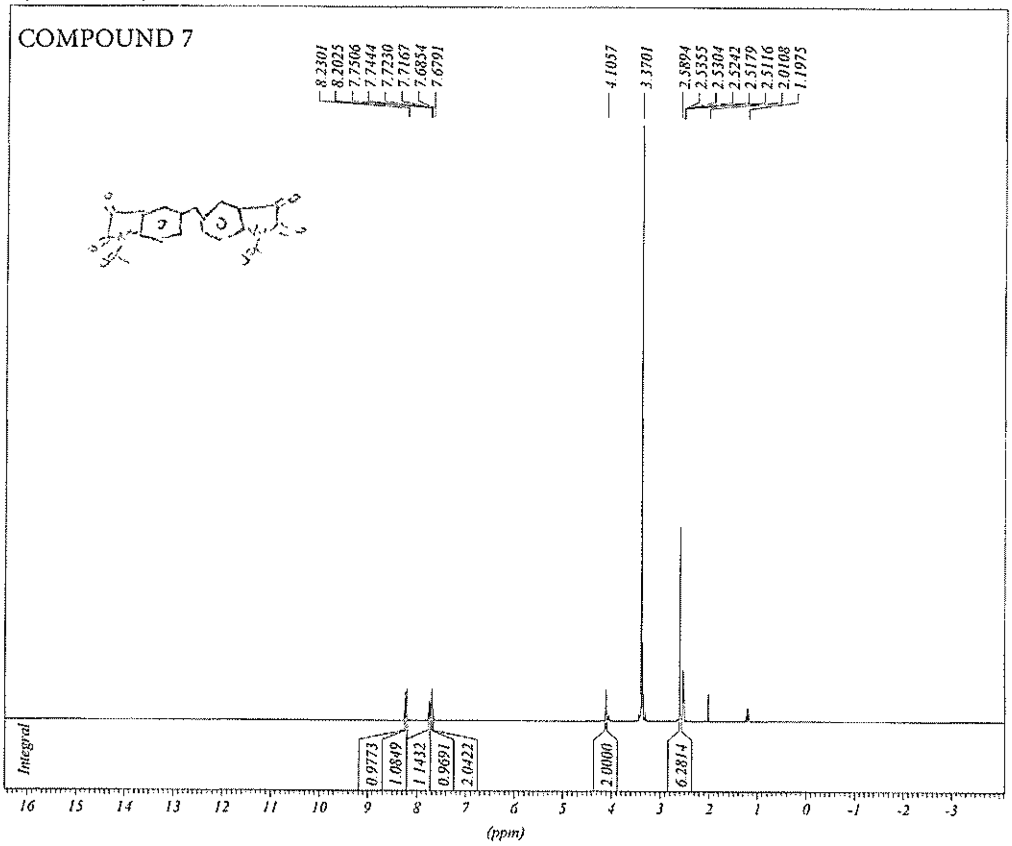

*** Current Data Parameters ***

NAME	090612~1
EXPNO	20
PROCNO	1

*** Acquisition Parameters ***

DATE_t	01:26:58
DATE_d	Jun 12 2009
DS	2
LOCNUC	2H
NS	16
O1	1853.67 Hz
O2	1853.67 Hz
RG	406.3999939
SFO1	300.1718537 MHz
SOLVENT	DMSO
SW	20.5644 ppm
TD	32768
TE	298.2 K

*** Processing Parameters ***

LB	0.30 Hz
PC	1.00
SF	300.1700000 MHz
SI	16384

*** 1D NMR Plot Parameters ***

SR	-0.00 Hz
SOLVENT	?

9

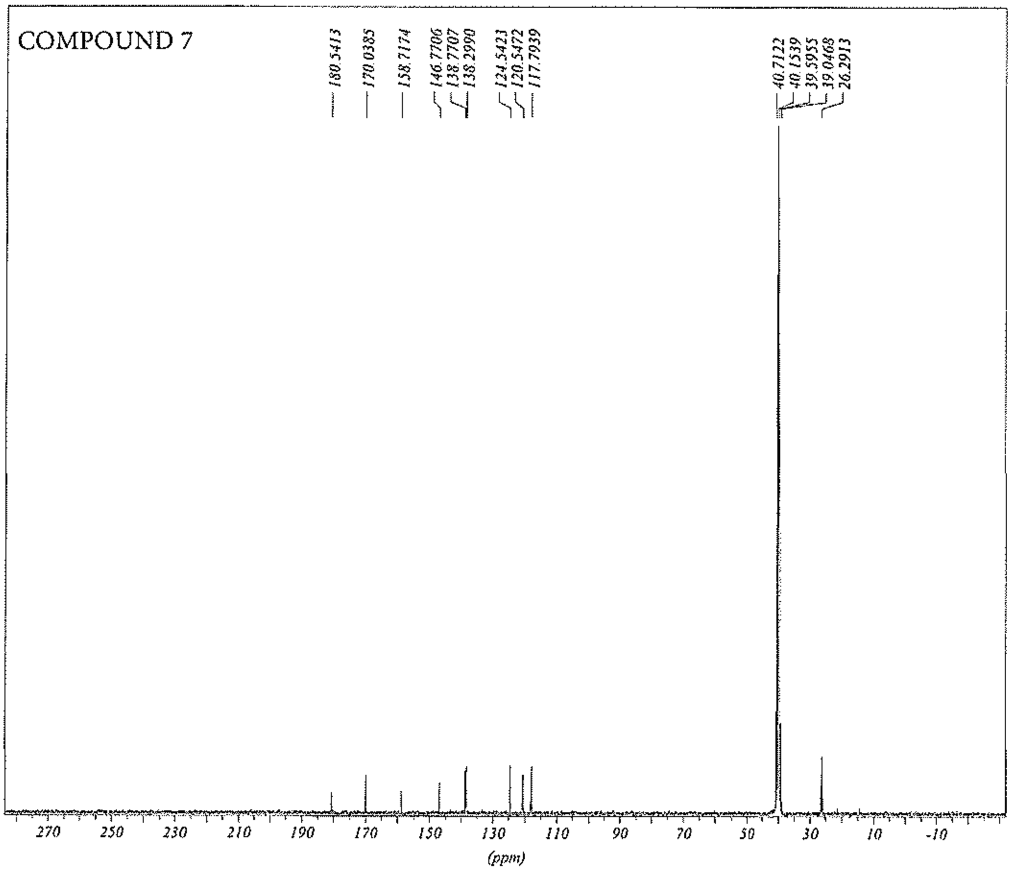

*** Current Data Parameters ***

NAME	090612~1
EXPNO	21
PROCNO	1

*** Acquisition Parameters ***

DATE_t	03:25:11
DATE_d	Jun 12 2009
DS	2
LOCNUC	2H
NS	2048
O1	9532.38 Hz
O2	1500.85 Hz
RG	16384.0000000
SFO1	75.4873094 MHz
SOLVENT	DMSO
SW	315.4109 ppm
TD	65536
TE	298.2 K

*** Processing Parameters ***

LB	1.00 Hz
PC	1.40
SF	75.4778147 MHz
SI	32768

*** 1D NMR Plot Parameters ***

SR	37.74 Hz
SOLVENT	?

Supervisor Kumar
acyl-bisisatin5
1H DMSO F:\\zrn 12

COMPOUND 8

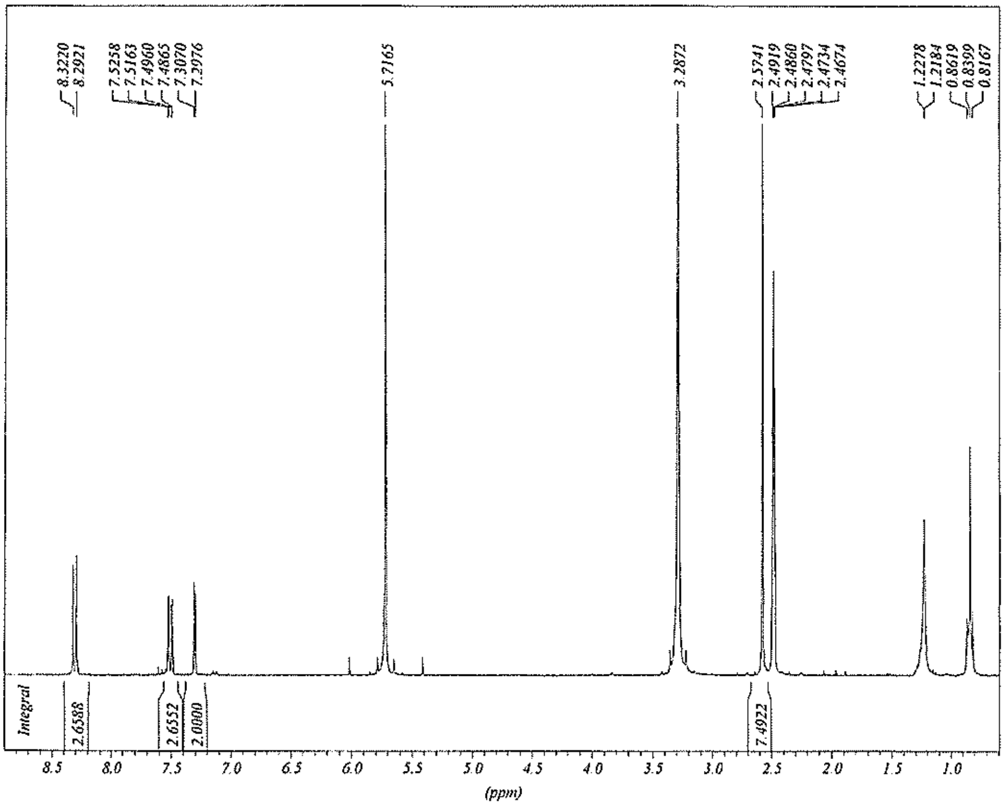

*** Current Data Parameters ***

NAME	:	0AQVQ7~L
EXPNO	:	4
PROCNO	:	1

*** Acquisition Parameters ***

DATE_t	:	13:30:26
DATE_d	:	Sep 05 2009
DS	:	4
LOCNUC	:	2H
NS	:	16
O1	:	1853.67 Hz
O2	:	0.00 Hz
RG	:	362.0000000
SFO1	:	300.1318537 MHz
SOLVENT	:	DMSO
SW	:	20.5671 ppm
TD	:	32768
TE	:	298.0 K

*** Processing Parameters ***

LB	:	0.30 Hz
PC	:	1.00
SF	:	300.1300071 MHz
SI	:	65536

*** 1D NMR Plot Parameters ***

SR	:	7.14 Hz
SOLVENT	:	?

Supervisor Kumaracyl-bisisatin5

COMPOUND 8

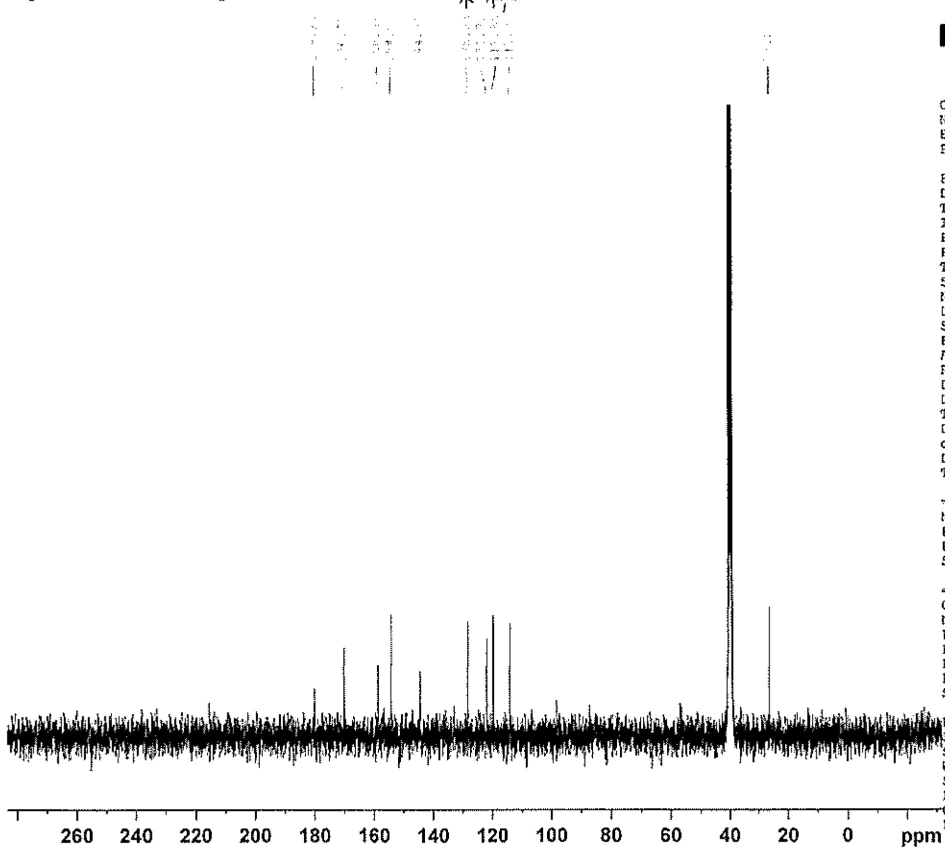

BRUKER

Current Data Parameters

NAME	101208-zrn	
EXPNO	31	
PROCNO	1	

F2 - Acquisition Parameters

Date_	20101209	
Time	7.29	
INSTRUM	dpx1	
PROBHD	5 mm QNP 1H/13	
PULPROG	zgpg30	
TD	65536	
SOLVENT	DMSO	
NS	1024	
DS	2	
SWH	23809.523	Hz
FIDRES	0.363304	Hz
AQ	1.3763061	sec
RG	16384	
DW	21.000	usec
DE	25.00	usec
TE	293.6	K
D1	2.00000000	sec
d11	0.03000000	sec
DELTA	1.89999998	sec
TD0	1	

======== CHANNEL f1 ========

NUC1	13C	
P1	7.50	usec
PL1	-3.00	dB
SFO1	75.4873094	MHz

======== CHANNEL f2 ========

CPDPRG2	waltz16	
NUC2	1H	
PCPD2	105.00	usec
PL2	-3.00	dB
PL12	17.34	dB
PL13	23.60	dB
SFO2	300.1715008	MHz

F2 - Processing parameters

SI	32768	
SF	75.4778155	MHz
WDW	EM	
SSB	0	
LB	1.00	Hz
GB	0	
PC	1.40	

8

Supervisor Kumarbisisatin 4 + MeOH

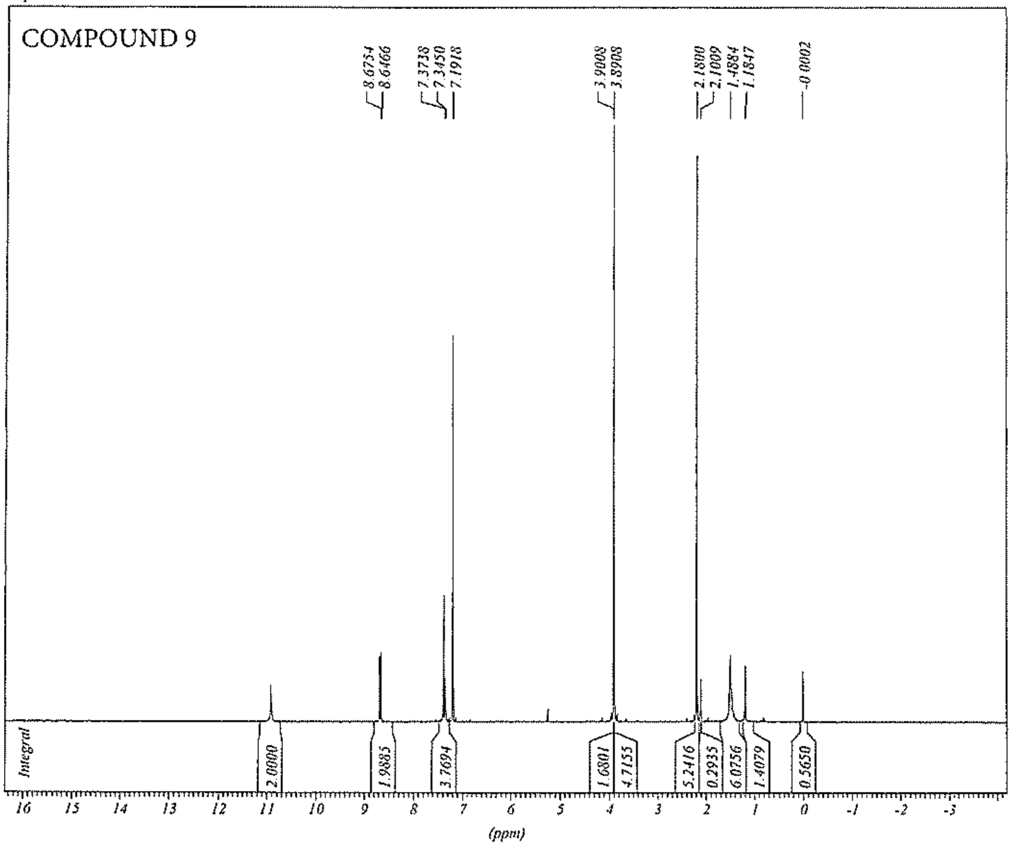

*** Current Data Parameters ***

NAME	02UNV8-H
EXPNO	20
PROCNO	1

*** Acquisition Parameters ***

DATE_t	07:13:25
DATE_d	Jul 21 2009
DS	2
LOCNUC	2H
NS	16
O1	1853.67 Hz
O2	1853.67 Hz
RG	1149.4000244
SFO1	300.1718537 MHz
SOLVENT	CDCl3
SW	20.5644 ppm
TD	32768
TE	300.2 K

*** Processing Parameters ***

LB	0.30 Hz
PC	1.00
SF	300.1700257 MHz
SI	16384

*** 1D NMR Plot Parameters ***

SR	25.69 Hz
SOLVENT	?

Supervisor Kumarbisisatin 4 + MeOH

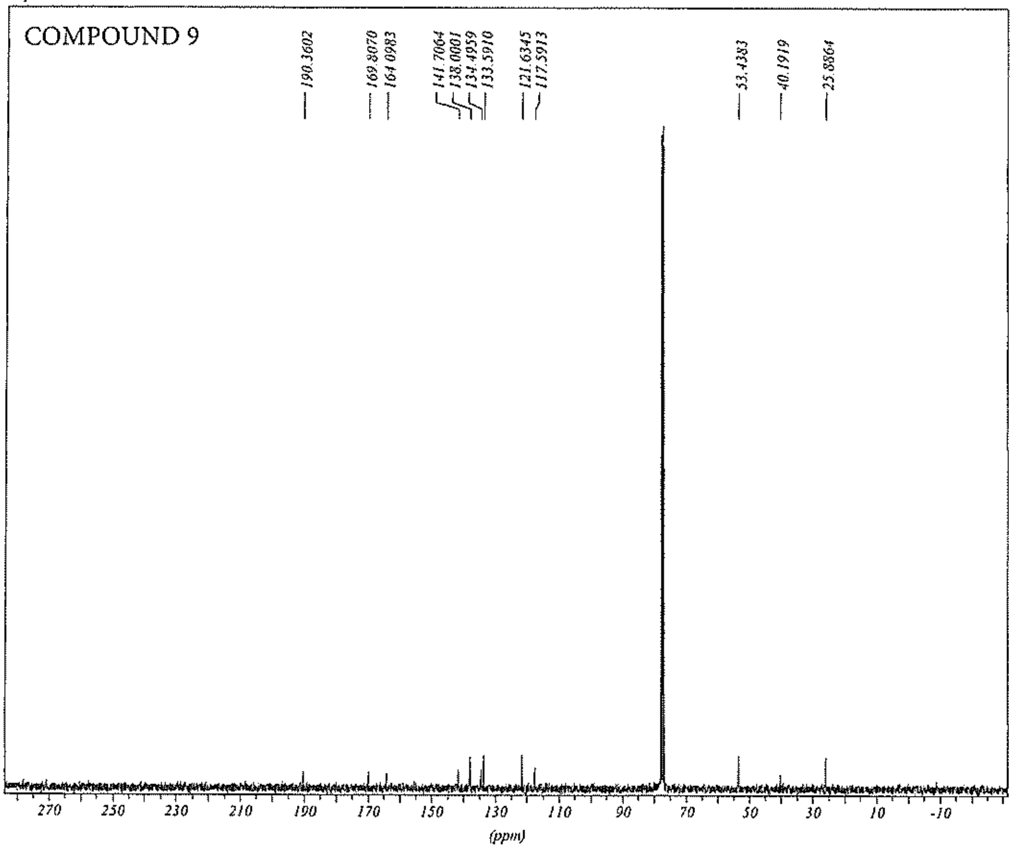

*** Current Data Parameters ***

NAME	02UNV8-H
EXPNO	21
PROCNO	1

*** Acquisition Parameters ***

DATE_t	09:11:41
DATE_d	Jul 21 2009
DS	2
LOCNUC	2H
NS	2048
O1	9532.38 Hz
O2	1500.85 Hz
RG	16384.0000000
SFO1	75.4873094 MHz
SOLVENT	CDCl3
SW	315.4109 ppm
TD	65536
TE	300.2 K

*** Processing Parameters ***

LB	1.00 Hz
PC	1.40
SF	75.4777770 MHz
SI	32768

*** 1D NMR Plot Parameters ***

SR	-0.00 Hz
SOLVENT	?

Supervisor KumarMeOH+bisisatin5

COMPOUND 11

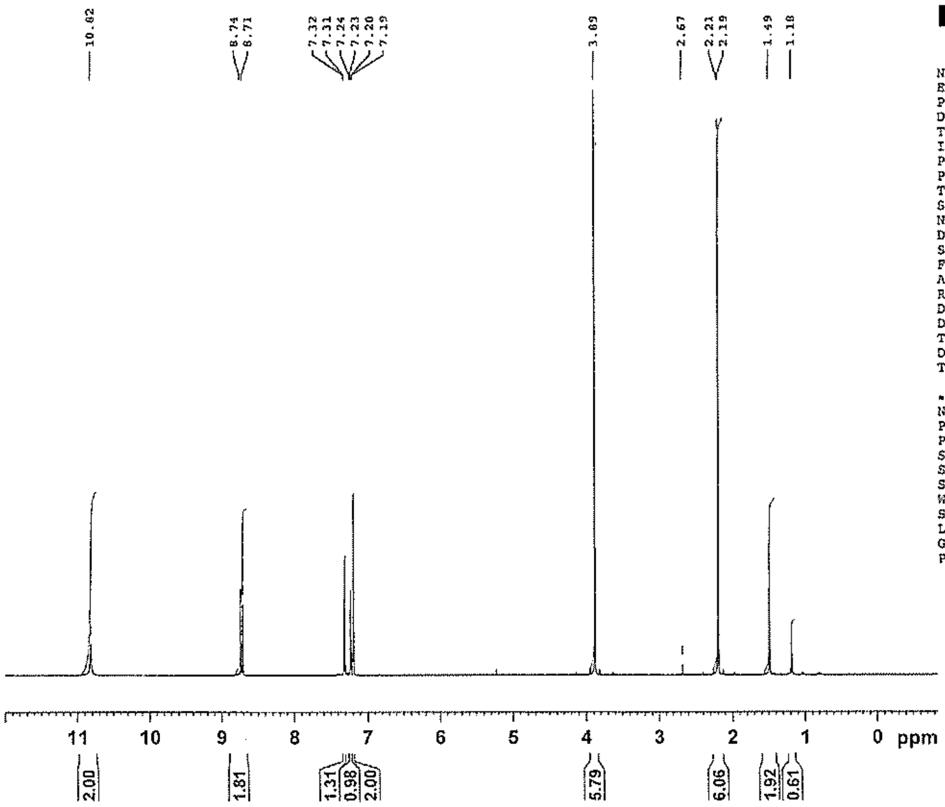

NAME	090911-zrn	
EXPNO	20	
PROCNO	1	
Date_	20091109	
Time	16.08	
INSTRUM	dpx1	
PROBHD	5 mm QNP 1H/13	
PULPROG	zg	
TD	32768	
SOLVENT	CDCl3	
NS	16	
DS	2	
SWH	6172.839	Hz
FIDRES	0.188380	Hz
AQ	2.6542580	sec
RG	645.1	
DW	81.000	usec
DE	12.00	usec
TE	298.2	K
D1	1.50000000	sec
TD0	1	
======== CHANNEL f1 ========		
NUC1	1H	
P1	10.10	usec
PL1	-3.00	dB
SFO1	300.1718537	MHz
SI	65536	
SF	300.1700318	MHz
WDW	EM	
SSB	0	
LB	0.30	Hz
GB	0	
PC	1.00	

24

Supervisor KumarMeOH+bisisatin5

COMPOUND 11

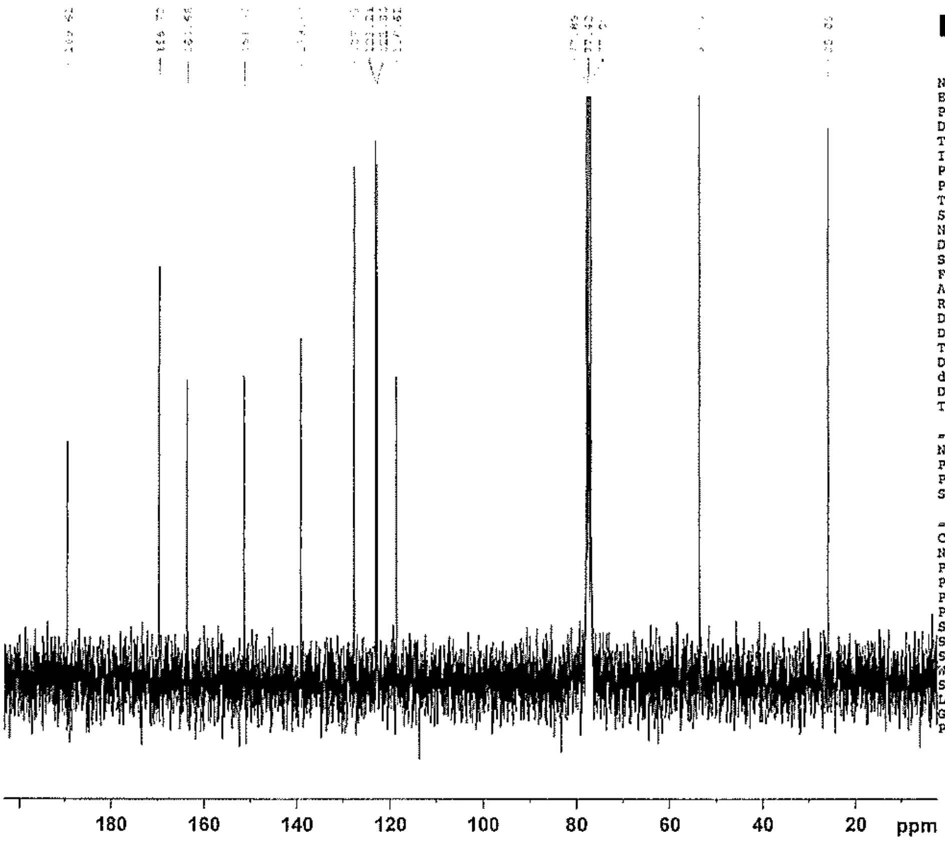

NAME	090911-zrn	
EXPNO	21	
PROCNO	1	
Date_	20091109	
Time	18.06	
INSTRUM	dpx1	
PROBHD	5 mm QNP 1H/13	
PULPROG	zgpg30	
TD	65536	
SOLVENT	CDCl3	
NS	2048	
DS	2	
SWH	23809.523	Hz
FIDRES	0.363304	Hz
AQ	1.3763061	sec
RG	16384	
DW	21.000	usec
DE	25.00	usec
TE	298.2	K
D1	2.00000000	sec
d11	0.03000000	sec
DELTA	1.89999998	sec
TD0	1	
======== CHANNEL f1 ========		
NUC1	13C	
P1	7.50	usec
PL1	-3.00	dB
SFO1	75.4873094	MHz
======== CHANNEL f2 ========		
CPDPRG2	waltz16	
NUC2	1H	
PCPD2	105.00	usec
PL2	-3.00	dB
PL12	17.34	dB
PL13	23.60	dB
SFO2	300.1715008	MHz
SI	32768	
SF	75.4777770	MHz
WDW	EM	
SSB	0	
LB	1.00	Hz
GB	0	
PC	1.40	

Supervisor Kumar
EtOH+bisisatin 5
1H CDCl3 F:\\ zrn 20

COMPOUND 12

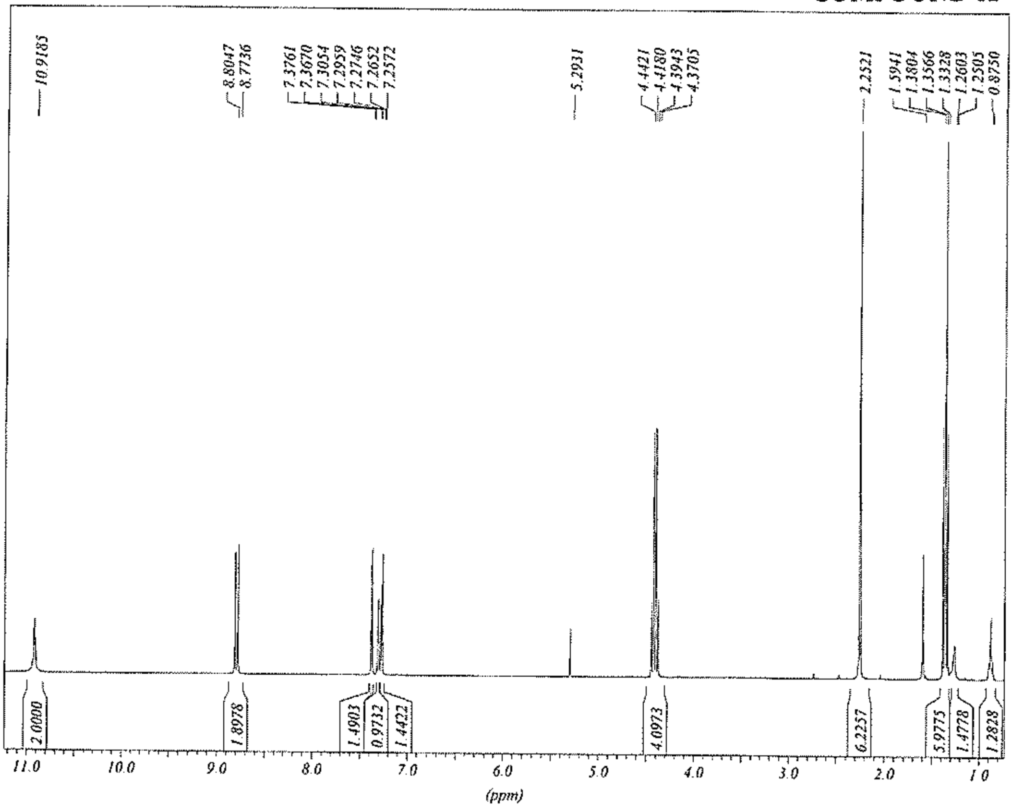

*** Current Data Parameters ***

NAME	:	0YF2PZ-5
EXPNO	:	5
PROCNO	:	1

*** Acquisition Parameters ***

DATE_t	:	00:06:26
DATE_d	:	Nov 06 2009
DS	:	0
LOCNUC	:	2H
NS	:	16
O1	:	2100.91 Hz
O2	:	0.00 Hz
RG	:	287.3999939
SFO1	:	300.1321009 MHz
SOLVENT	:	CDCl3
SW	:	19.9752 ppm
TD	:	32768
TE	:	298.0 K

*** Processing Parameters ***

LB	:	0.30 Hz
PC	:	1.00
SF	:	300.1300071 MHz
SI	:	65536

*** 1D NMR Plot Parameters ***

SR	:	7.14 Hz
SOLVENT	:	?

25

Supervisor Kumar
EtOH+bisisatin 5
13C{1H} CDCl3 F:\\ zrn 20

COMPOUND 12

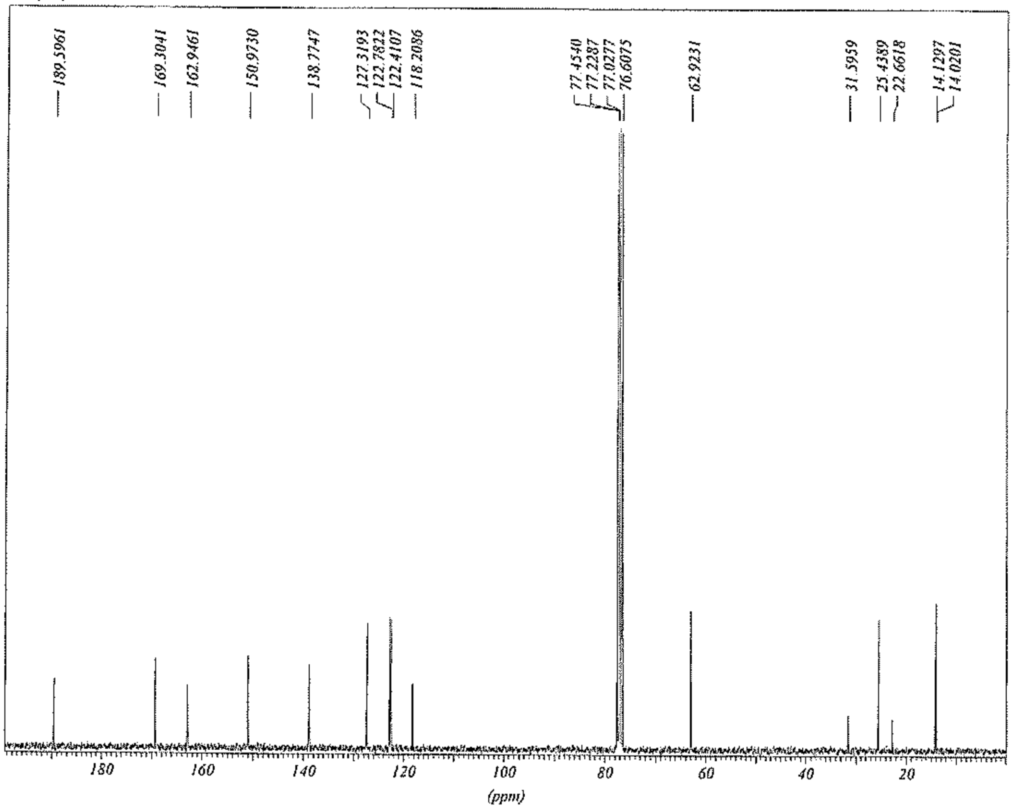

*** Current Data Parameters ***

NAME	:	0YF2PZ-5
EXPNO	:	6
PROCNO	:	1

*** Acquisition Parameters ***

DATE_t	:	01:55:33
DATE_d	:	Nov 06 2009
DS	:	0
LOCNUC	:	2H
NS	:	2048
O1	:	7546.77 Hz
O2	:	1500.85 Hz
RG	:	18390.4003906
SFO1	:	75.4752658 MHz
SOLVENT	:	CDCl3
SW	:	199.5388 ppm
TD	:	32768
TE	:	297.9 K

*** Processing Parameters ***

LB	:	1.00 Hz
PC	:	1.40
SF	:	75.4677485 MHz
SI	:	32768

*** 1D NMR Plot Parameters ***

SR	:	29.46 Hz
SOLVENT	:	?

Supervisor Kumar
NH3+bisisatin4
1H DMSO F:\\ zrn 9

COMPOUND 13

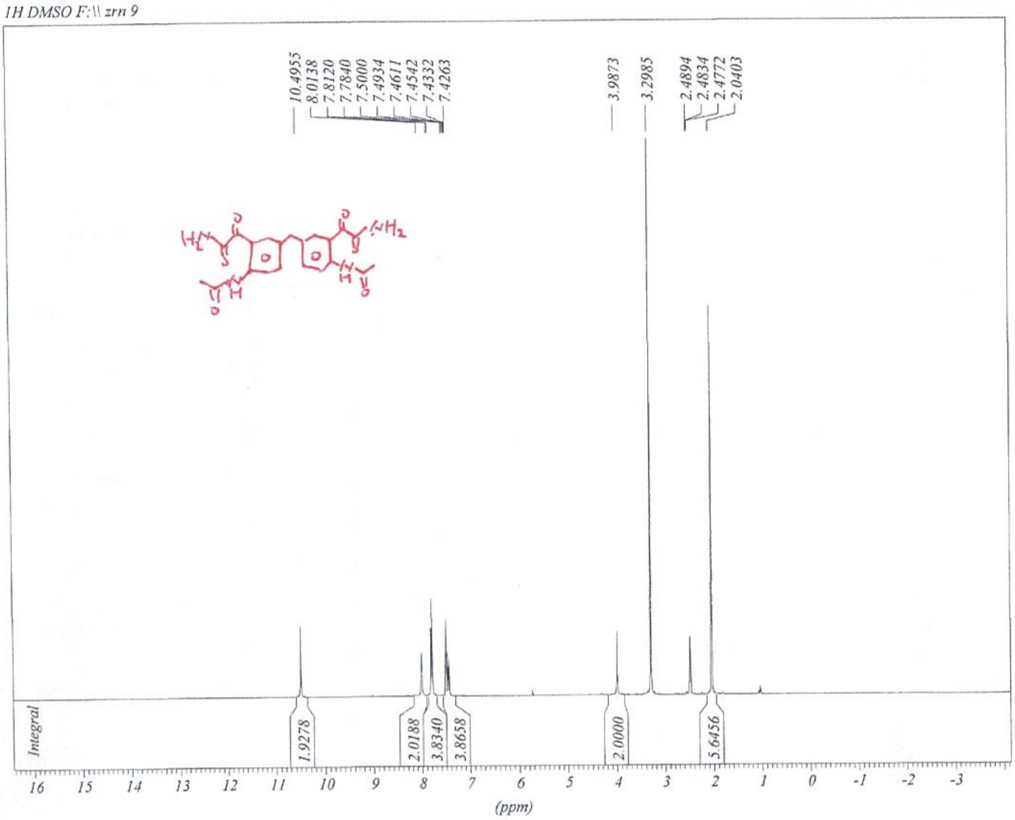

*** Current Data Parameters ***

NAME	:	0G8AGH~1
EXPNO	:	4
PROCNO	:	1

*** Acquisition Parameters ***

DATE_t	:	12:44:46
DATE_d	:	Aug 31 2009
DS	:	4
LOCNUC	:	2H
NS	:	16
O1	:	1853.67 Hz
O2	:	0.00 Hz
RG	:	362.0000000
SFO1	:	300.1318537 MHz
SOLVENT	:	DMSO
SW	:	20.5671 ppm
TD	:	32768
TE	:	298.0 K

*** Processing Parameters ***

LB	:	0.30 Hz
PC	:	1.00
SF	:	300.1300071 MHz
SI	:	65536

*** 1D NMR Plot Parameters ***

SR	:	7.14 Hz
SOLVENT	:	?

14

Supervisor Kumar
NH3+bisisatin4
13C{1H} DMSO F:\\ zrn 9

COMPOUND 13

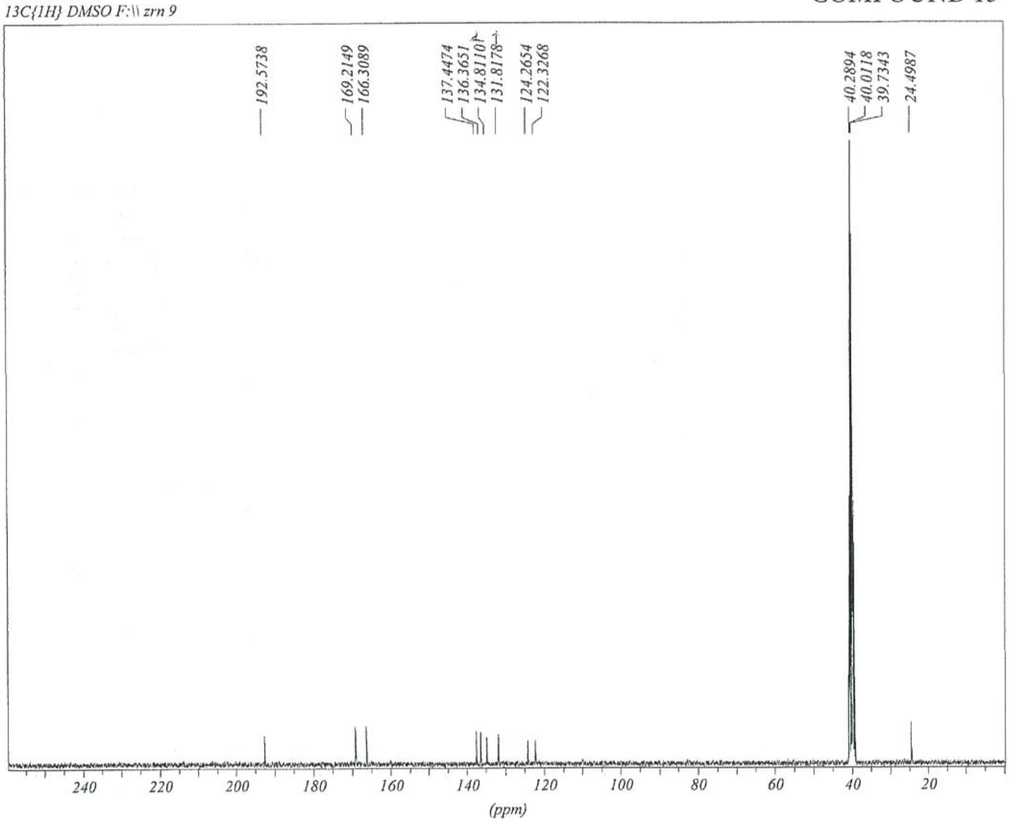

*** Current Data Parameters ***

NAME	:	0G8AGH~1
EXPNO	:	5
PROCNO	:	1

*** Acquisition Parameters ***

DATE_t	:	13:50:17
DATE_d	:	Aug 31 2009
DS	:	0
LOCNUC	:	2H
NS	:	1024
O1	:	9810.80 Hz
O2	:	1500.85 Hz
RG	:	16384.0000000
SFO1	:	75.4775298 MHz
SOLVENT	:	DMSO
SW	:	259.7838 ppm
TD	:	65536
TE	:	298.0 K

*** Processing Parameters ***

LB	:	1.00 Hz
PC	:	1.40
SF	:	75.4677485 MHz
SI	:	65536

*** 1D NMR Plot Parameters ***

SR	:	29.46 Hz
SOLVENT	:	?

Supervisor Kumar
NH3+bisisatin 5
1H DMSO F:\\ zrn 19

COMPOUND 14

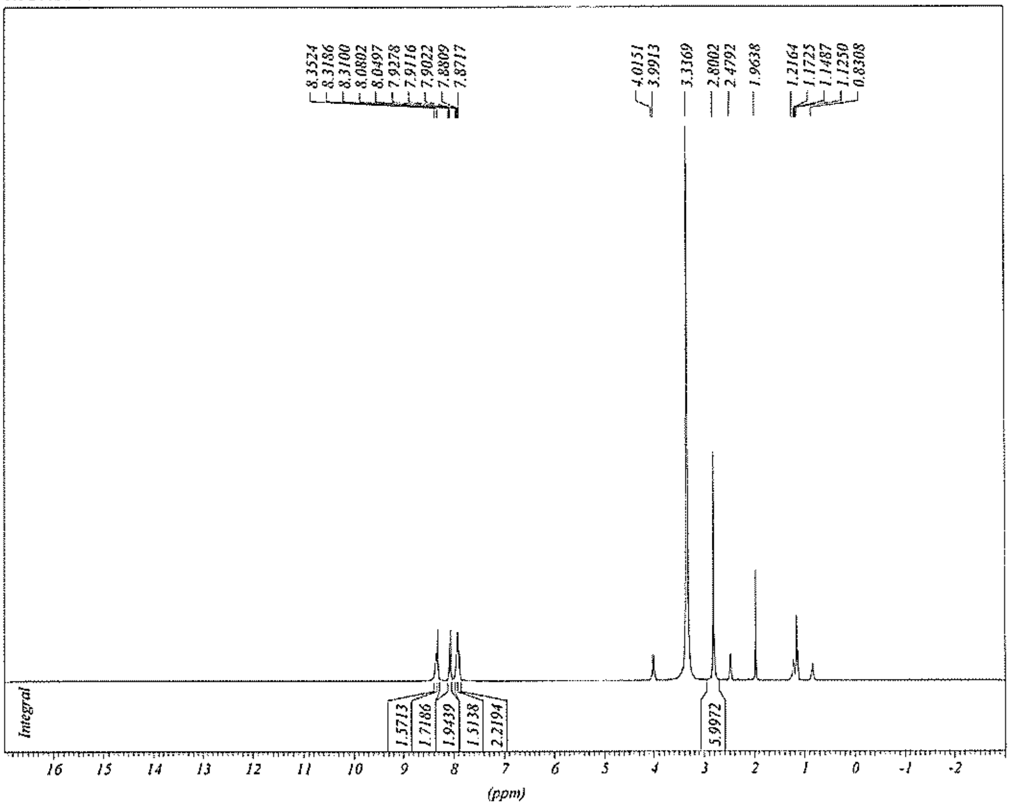

*** Current Data Parameters ***

NAME : 0YF2PZ~5
EXPNO : 2
PROCNO : 1

*** Acquisition Parameters ***

DATE_t : 21:10:57
DATE_d : Nov 05 2009
DS : 0
LOCNUC : 2H
NS : 16
O1 : 2100.91 Hz
O2 : 0.00 Hz
RG : 143.6999969
SFO1 : 300.1321009 MHz
SOLVENT : DMSO
SW : 19.9752 ppm
TD : 32768
TE : 298.3 K

*** Processing Parameters ***

LB : 0.30 Hz
PC : 1.00
SF : 300.1300071 MHz
SI : 65536

*** 1D NMR Plot Parameters ***

SR : 7.14 Hz
SOLVENT : ?

15

Supervisor Kumar
NH3+bisisatin 5
13C{1H} DMSO F:\\ zrn 19

COMPOUND 14

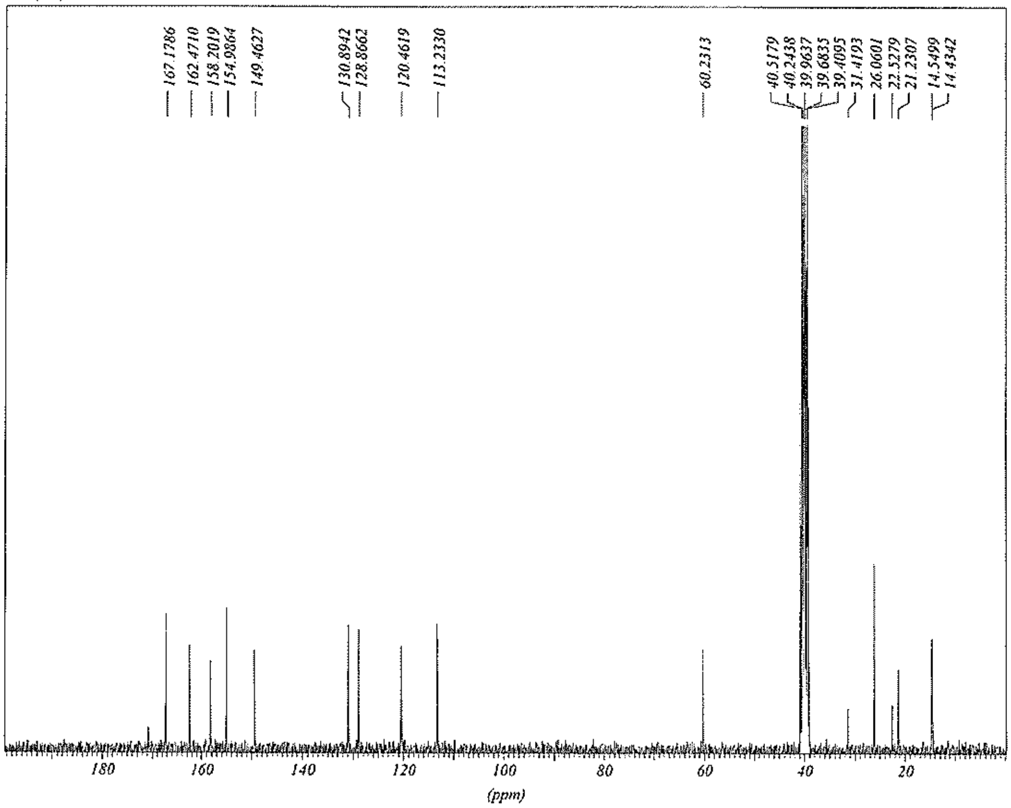

*** Current Data Parameters ***

NAME : 0YF2PZ~5
EXPNO : 3
PROCNO : 1

*** Acquisition Parameters ***

DATE_t : 23:00:24
DATE_d : Nov 05 2009
DS : 0
LOCNUC : 2H
NS : 2048
O1 : 7546.77 Hz
O2 : 1500.85 Hz
RG : 16384.0000000
SFO1 : 75.4752658 MHz
SOLVENT : DMSO
SW : 199.5368 ppm
TD : 32768
TE : 297.6 K

*** Processing Parameters ***

LB : 1.00 Hz
PC : 1.40
SF : 75.4677485 MHz
SI : 32768

*** 1D NMR Plot Parameters ***

SR : 29.46 Hz
SOLVENT : ?

Supervisor Kumarbutylamine + bisisatin4

COMPOUND 15

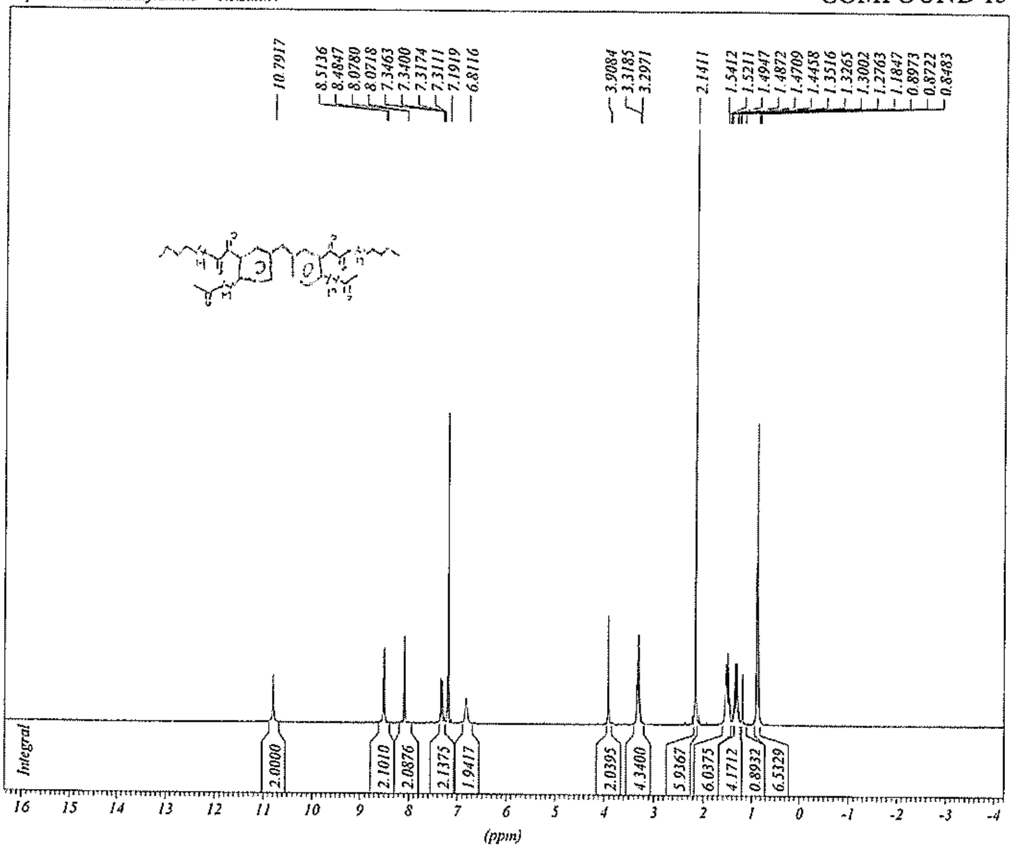

*** *Current Data Parameters* ***

NAME	:	097KL5~8
EXPNO	:	20
PROCNO	:	1

*** *Acquisition Parameters* ***

DATE_t	:	10:41:04
DATE_d	:	Aug 27 2009
DS	:	2
LOCNUC	:	2H
NS	:	16
O1	:	1853.67 Hz
O2	:	1853.67 Hz
RG	:	574.7000122
SFO1	:	300.1718537 MHz
SOLVENT	:	CDCl3
SW	:	20.5644 ppm
TD	:	32768
TE	:	300.2 K

*** *Processing Parameters* ***

LB	:	0.30 Hz
PC	:	1.00
SF	:	300.1700321 MHz
SI	:	16384

*** *1D NMR Plot Parameters* ***

SR	:	32.08 Hz
SOLVENT	:	?

16

Supervisor Kumarbutylamine + bisisatin4

COMPOUND 15

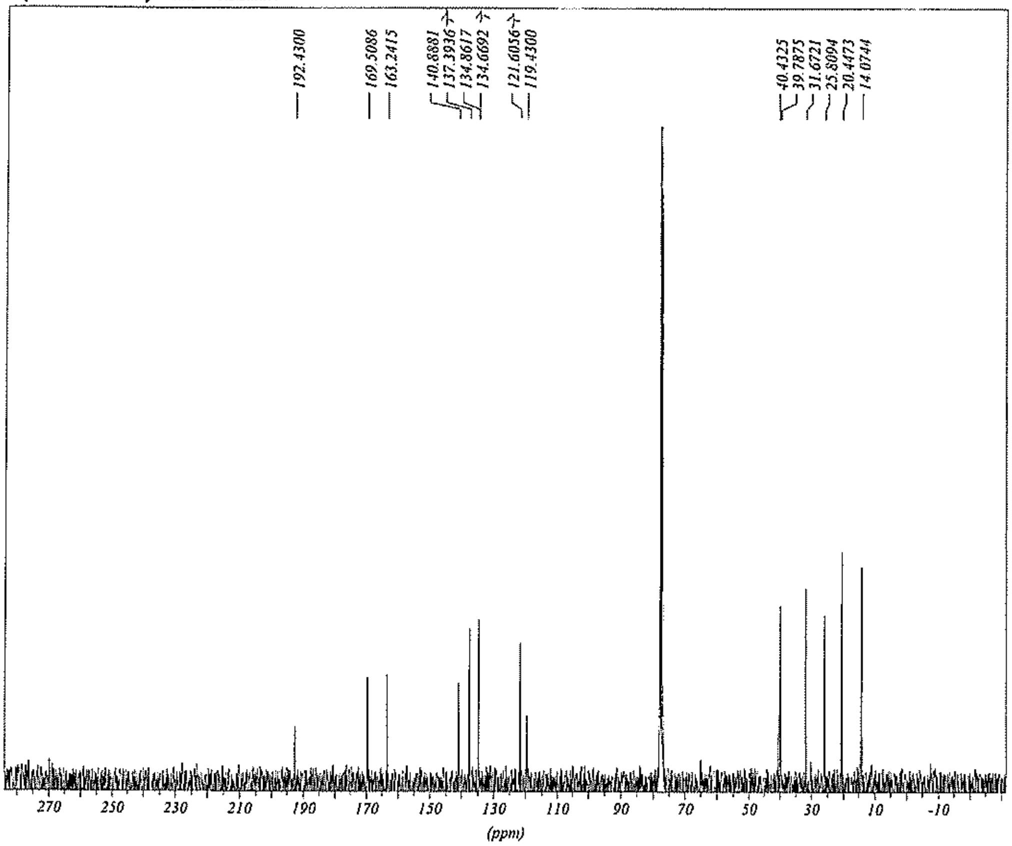

*** *Current Data Parameters* ***

NAME	:	097KL5~8
EXPNO	:	21
PROCNO	:	1

*** *Acquisition Parameters* ***

DATE_t	:	12:39:22
DATE_d	:	Aug 27 2009
DS	:	2
LOCNUC	:	2H
NS	:	2048
O1	:	9532.38 Hz
O2	:	1500.85 Hz
RG	:	16384.0000000
SFO1	:	75.4873094 MHz
SOLVENT	:	CDCl3
SW	:	315.4109 ppm
TD	:	65536
TE	:	300.2 K

*** *Processing Parameters* ***

LB	:	1.00 Hz
PC	:	1.40
SF	:	75.4777770 MHz
SI	:	32768

*** *1D NMR Plot Parameters* ***

SR	:	-0.00 Hz
SOLVENT	:	?

Supervisor Kumar
butylamine+bisisatin5
1H CDCl3 F:\\zrn 5

COMPOUND 16

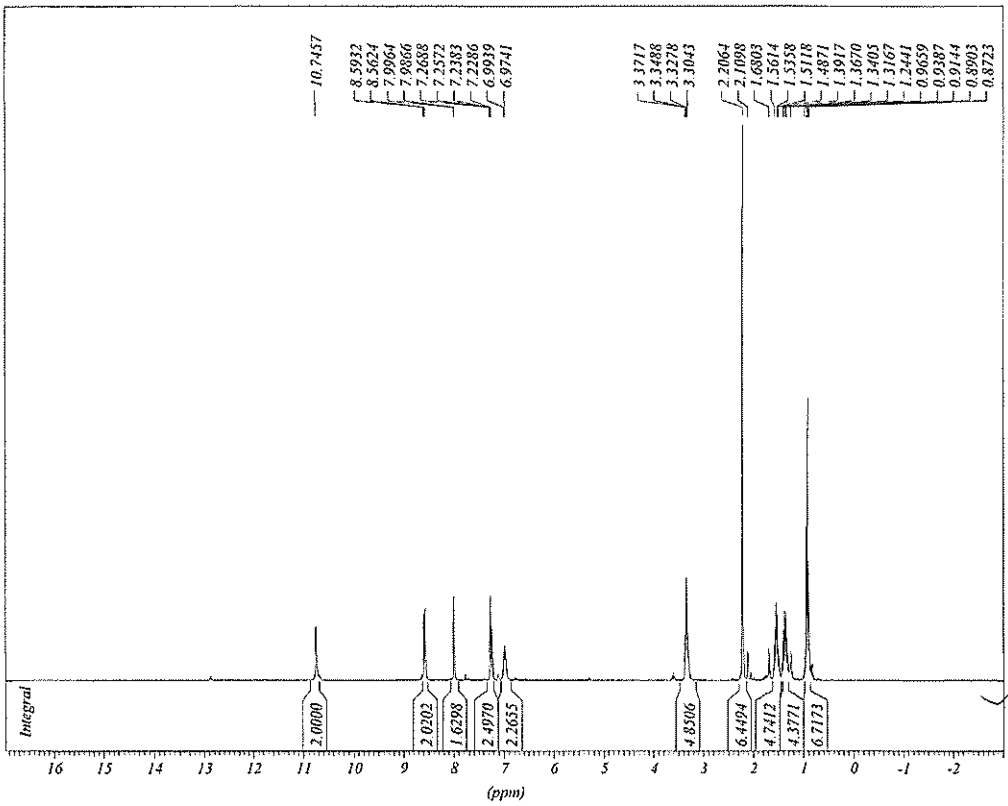

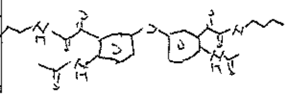

*** Current Data Parameters ***

NAME	:	0XTMCT-3
EXPNO	:	1
PROCNO	:	1

*** Acquisition Parameters ***

DATE_t	:	01:01:03
DATE_d	.	Oct 27 2009
DS	:	0
LOCNUC	:	2H
NS	:	16
O1	·	2100.91 Hz
O2	.	0.00 Hz
RG	:	143.6999969
SFO1		300.1321009 MHz
SOLVENT	:	CDCl3
SW		19.9752 ppm
TD		32768
TE	:	297.9 K

*** Processing Parameters ***

LB		0.30 Hz
PC		1.00
SF	.	300.1300071 MHz
SI		65536

*** 1D NMR Plot Parameters ***

SR		7.14 Hz
SOLVENT		?

17

Supervisor Kumar
butylamine+bisisatin5
13C{1H} CDCl3 F:\\zrn 5

COMPOUND 16

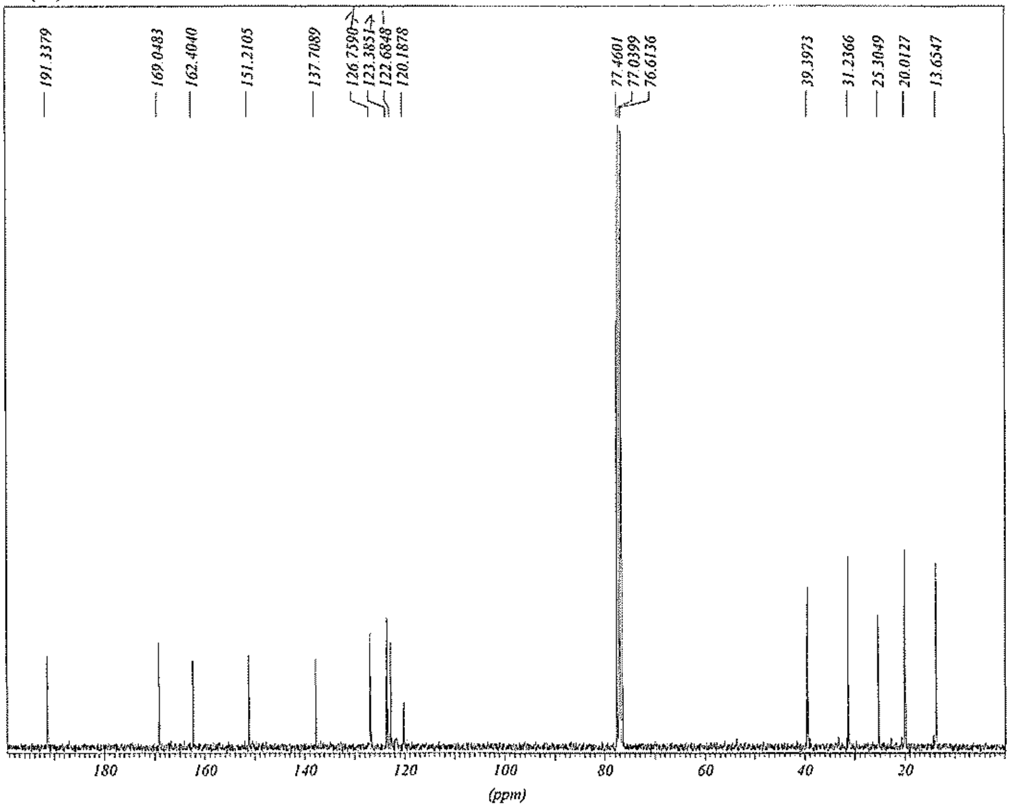

*** Current Data Parameters ***

NAME		0XTMCT-3
EXPNO	·	2
PROCNO	·	1

*** Acquisition Parameters ***

DATE_t	.	02:50:13
DATE_d	:	Oct 27 2009
DS	:	0
LOCNUC	:	2H
NS	:	2048
O1	:	7546.77 Hz
O2	:	1500.85 Hz
RG	:	16384.0000000
SFO1	:	75.4752658 MHz
SOLVENT	:	CDCl3
SW	:	199.5388 ppm
TD	:	32768
TE	:	297.8 K

*** Processing Parameters ***

LB		1.00 Hz
PC	:	1.40
SF	:	75.4677485 MHz
SI	:	32768

*** 1D NMR Plot Parameters ***

SR	:	29.46 Hz
SOLVENT	:	?

Supervisor Kumarbisisatin4 + piperidine

COMPOUND 17

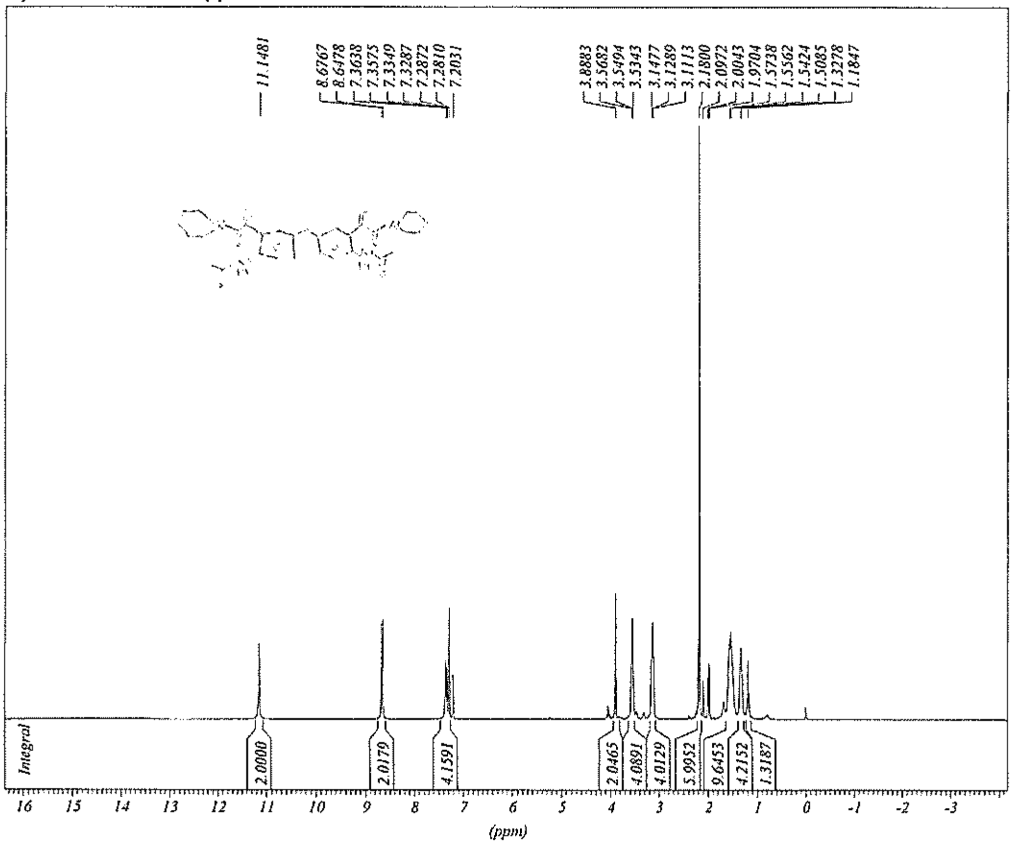

*** Current Data Parameters ***

NAME	:	0EE514~Q
EXPNO	:	30
PROCNO	:	1

*** Acquisition Parameters ***

DATE_t	:	22:52:20
DATE_d	:	Jul 18 2009
DS	:	2
LOCNUC	:	2H
NS	:	16
O1	:	1853.67 Hz
O2	:	1853.67 Hz
RG	:	203.1999969
SFO1	:	300.1718537 MHz
SOLVENT	:	CDCl3
SW	:	20.5644 ppm
TD	:	32768
TE	:	300.2 K

*** Processing Parameters ***

LB	:	0.30 Hz
PC	:	1.00
SF	:	300.1700223 MHz
SI	:	16384

*** 1D NMR Plot Parameters ***

SR	:	22.30 Hz
SOLVENT	:	?

12

Supervisor Kumarbisisatin4 + piperidine 13C

COMPOUND 17

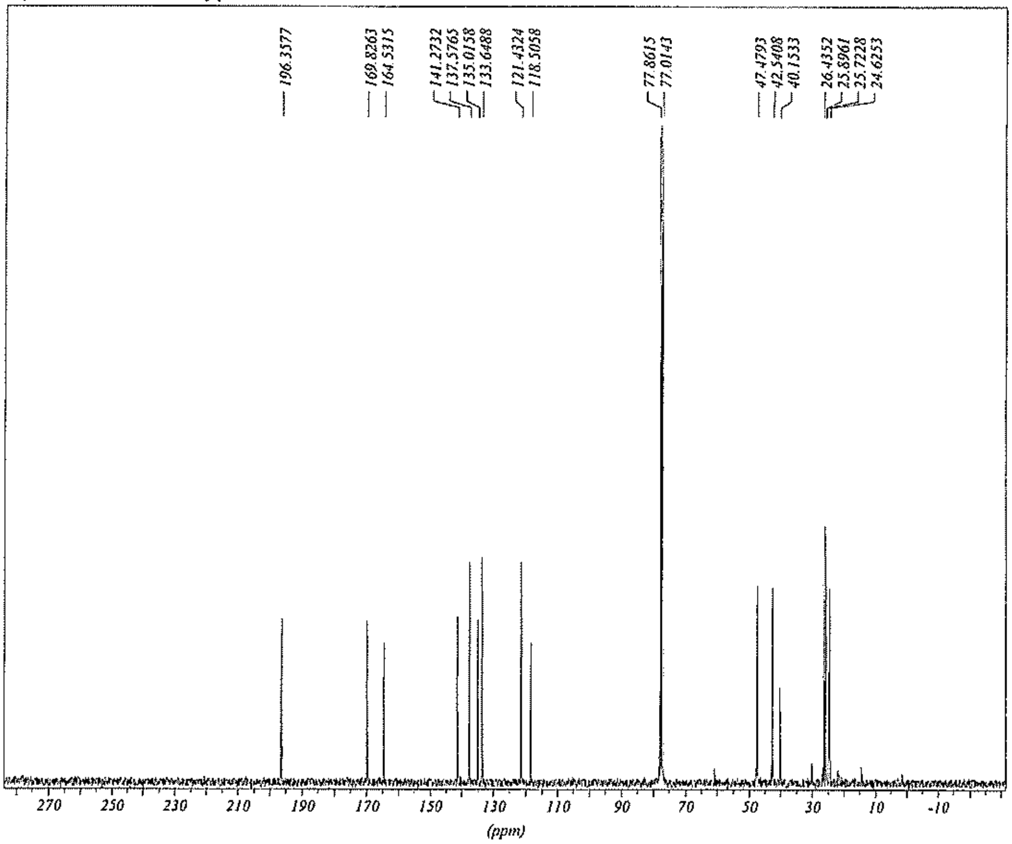

*** Current Data Parameters ***

NAME	:	0EE514~Q
EXPNO	:	31
PROCNO	:	1

*** Acquisition Parameters ***

DATE_t	:	00:50:42
DATE_d	:	Jul 19 2009
DS	:	2
LOCNUC	:	2H
NS	:	2048
O1	:	9532.38 Hz
O2	:	1500.85 Hz
RG	:	16384.0000000
SFO1	:	75.4873094 MHz
SOLVENT	:	CDCl3
SW	:	315.4109 ppm
TD	:	65536
TE	:	300.2 K

*** Processing Parameters ***

LB	:	1.00 Hz
PC	:	1.40
SF	:	75.4777770 MHz
SI	:	32768

*** 1D NMR Plot Parameters ***

SR	:	-0.00 Hz
SOLVENT	:	?

Supervisor Kumar
Pypiridine+bisisatin5
1H CDCl3 F:\\ zrn 49

COMPOUND 18

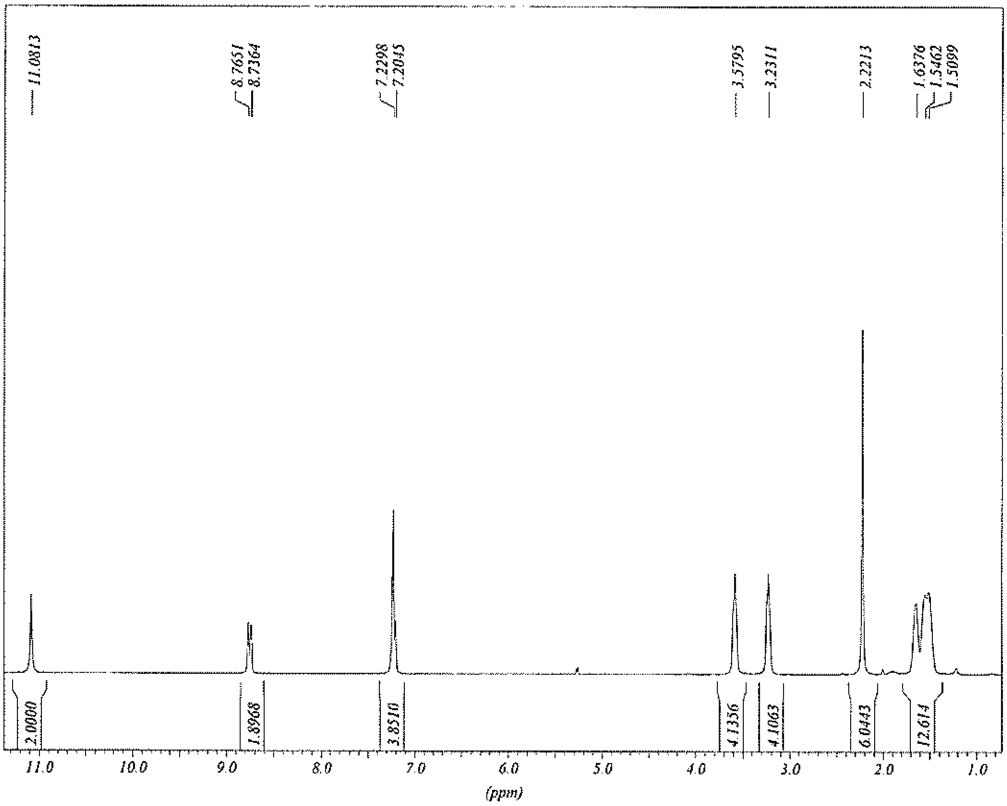

*** Current Data Parameters ***

NAME	:	0Z3MOS~T
EXPNO	:	4
PROCNO	:	1

*** Acquisition Parameters ***

DATE_t	:	00:44:36
DATE_d	:	Oct 29 2009
DS	:	0
LOCNUC	:	2H
NS	:	16
O1	:	2100.91 Hz
O2	:	0.00 Hz
RG	:	57.0000000
SFO1	:	300.1321009 MHz
SOLVENT	:	CDCl3
SW	:	19.9752 ppm
TD	:	32768
TE	:	297.9 K

*** Processing Parameters ***

LB	:	0.30 Hz
PC	:	1.00
SF	:	300.1300071 MHz
SI	:	65536

*** 1D NMR Plot Parameters ***

SR	:	7.14 Hz
SOLVENT	:	?

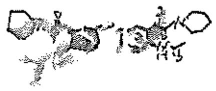

19

Supervisor Kumar
Pypiridine+bisisatin5
13C{1H} CDCl3 F:\\ zrn 49

COMPOUND 18

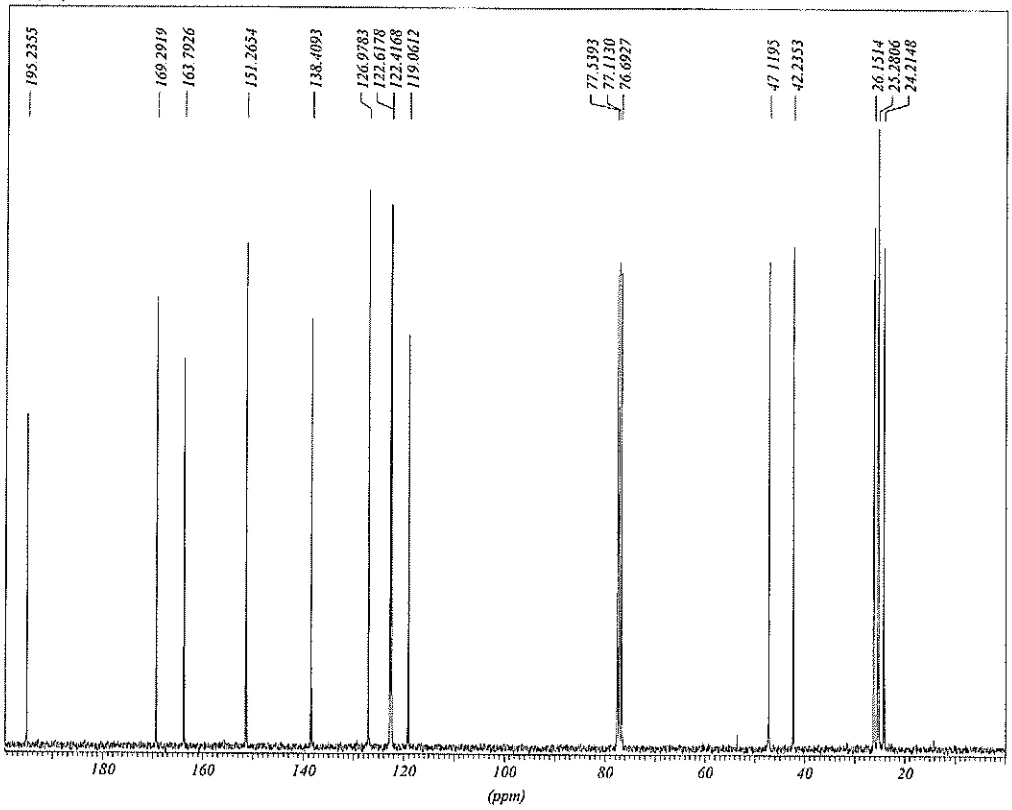

*** Current Data Parameters ***

NAME	:	0Z3MOS~T
EXPNO	:	5
PROCNO	:	1

*** Acquisition Parameters ***

DATE_t	:	02:33:52
DATE_d	:	Oct 29 2009
DS	:	0
LOCNUC	:	2H
NS	:	2048
O1	:	7546.77 Hz
O2	:	1500.85 Hz
RG	:	18390.4003906
SFO1	:	75.4752658 MHz
SOLVENT	:	CDCl3
SW	:	199.5388 ppm
TD	:	32768
TE	:	297.6 K

*** Processing Parameters ***

LB	:	1.00 Hz
PC	:	1.40
SF	:	75.4677485 MHz
SI	:	32768

*** 1D NMR Plot Parameters ***

SR	:	29.46 Hz
SOLVENT	:	?

Supervisor Kumarbisisatin 4+ gly

COMPOUND 21

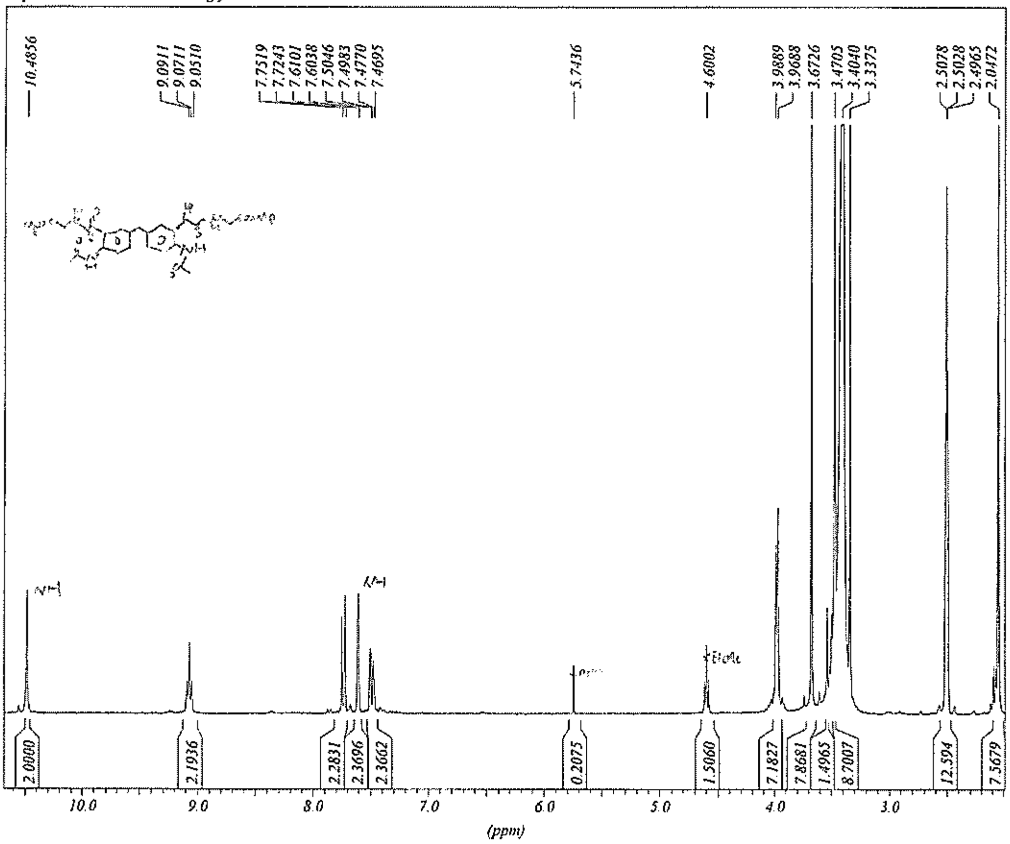

*** Current Data Parameters ***

NAME	:	0frew9~i
EXPNO	:	40
PROCNO	:	1

*** Acquisition Parameters ***

DATE_t	:	22:11:59
DATE_d	:	Jul 12 2009
DS	:	2
LOCNUC	:	2H
NS	:	16
O1	:	1853.67 Hz
O2	:	1853.67 Hz
RG	:	203.1999969
SFO1	:	300.1718537 MHz
SOLVENT	:	DMSO
SW	:	20.5644 ppm
TD	:	32768
TE	:	299.2 K

*** Processing Parameters ***

LB	:	0.30 Hz
PC	:	1.00
SF	:	300.1700000 MHz
SI	:	16384

*** 1D NMR Plot Parameters ***

SR	:	-0.00 Hz
SOLVENT	:	?

Supervisor Kumarbisisatin+leu

COMPOUND 22

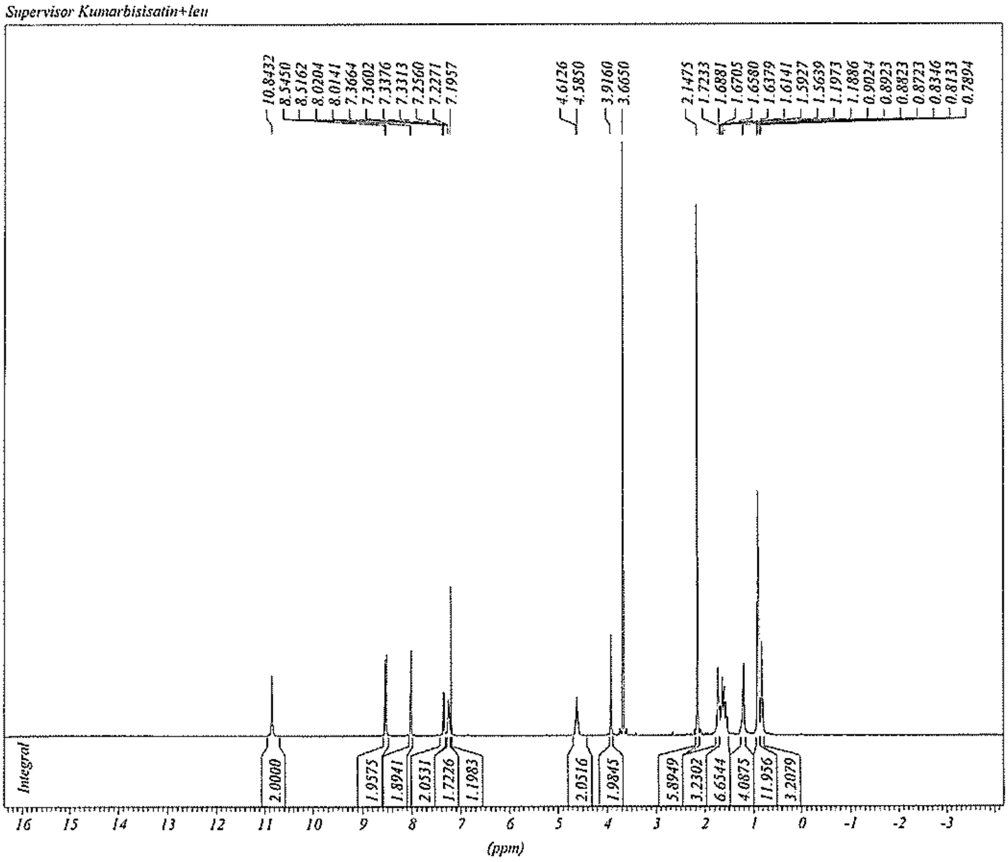

*** Current Data Parameters ***

NAME	:	0O5P2V~X
EXPNO	:	10
PROCNO	:	1

*** Acquisition Parameters ***

DATE_t	:	22:30:25
DATE_d	:	Jul 13 2009
DS	:	2
LOCNUC	:	2H
NS	:	16
O1	:	1853.67 Hz
O2	:	1853.67 Hz
RG	:	512.0000000
SFO1	:	300.1718537 MHz
SOLVENT	:	CDCl3
SW	:	20.5644 ppm
TD	:	32768
TE	:	299.2 K

*** Processing Parameters ***

LB	:	0.30 Hz
PC	:	1.00
SF	:	300.1700245 MHz
SI	:	16384

*** 1D NMR Plot Parameters ***

SR	:	24.52 Hz
SOLVENT	:	?

COMPOUND 22

Supervisor Kumarleu+bisisatin4

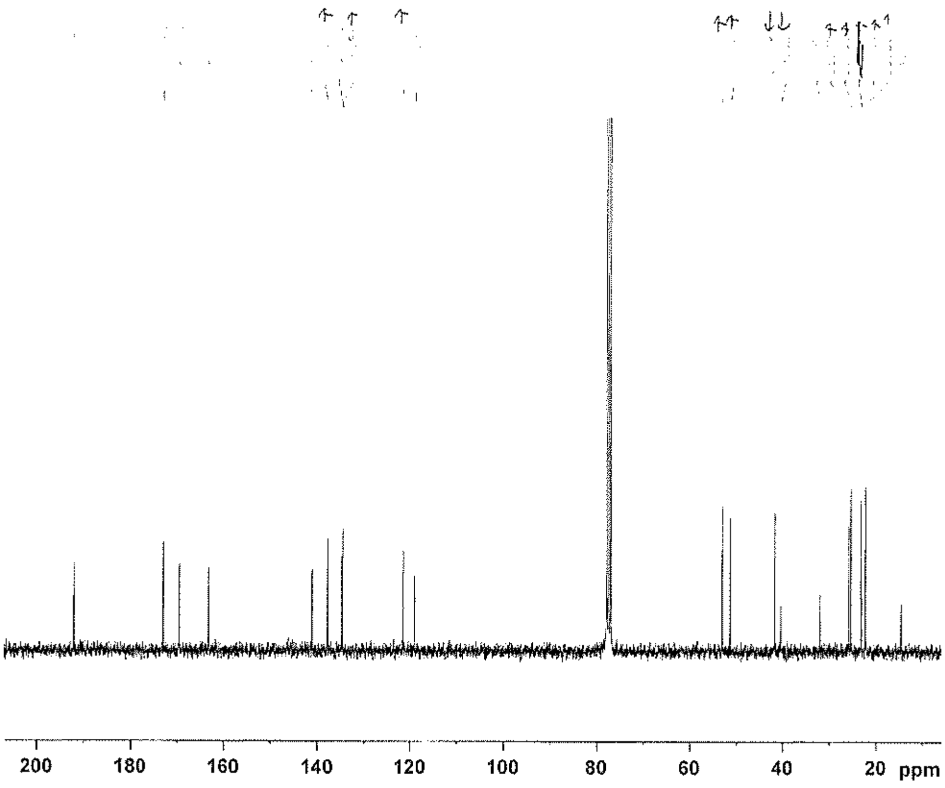

BRUKER

Current Data Parameters
NAME 090716-zrn
EXPNO 21
PROCNO 1

F2 - Acquisition Parameters
Date_ 20090717
Time 4.15
INSTRUM dpx1
PROBHD 5 mm QNP 1H/13
PULPROG zgpg30
TD 65536
SOLVENT CDCl3
NS 2048
DS 2
SWH 23809.523 Hz
FIDRES 0.363304 Hz
AQ 1.3763061 sec
RG 16384
DW 21.000 usec
DE 25.00 usec
TE 300.2 K
D1 2.00000000 sec
d11 0.03000000 sec
DELTA 1.89999998 sec
TD0 1

======== CHANNEL f1 ========
NUC1 13C
P1 7.50 usec
PL1 -3.00 dB
SFO1 75.4873094 MHz

======== CHANNEL f2 ========
CPDPRG2 waltz16
NUC2 1H
PCPD2 105.00 usec
PL2 -3.00 dB
PL12 17.60 dB
PL13 23.60 dB
SFO2 300.1715008 MHz

F2 - Processing parameters
SI 32768
SF 75.4777770 MHz
WDW EM
SSB 0
LB 1.00 Hz
GB 0
PC 1.40

COMPOUND 23

Supervisor Kumarbisisatin+met

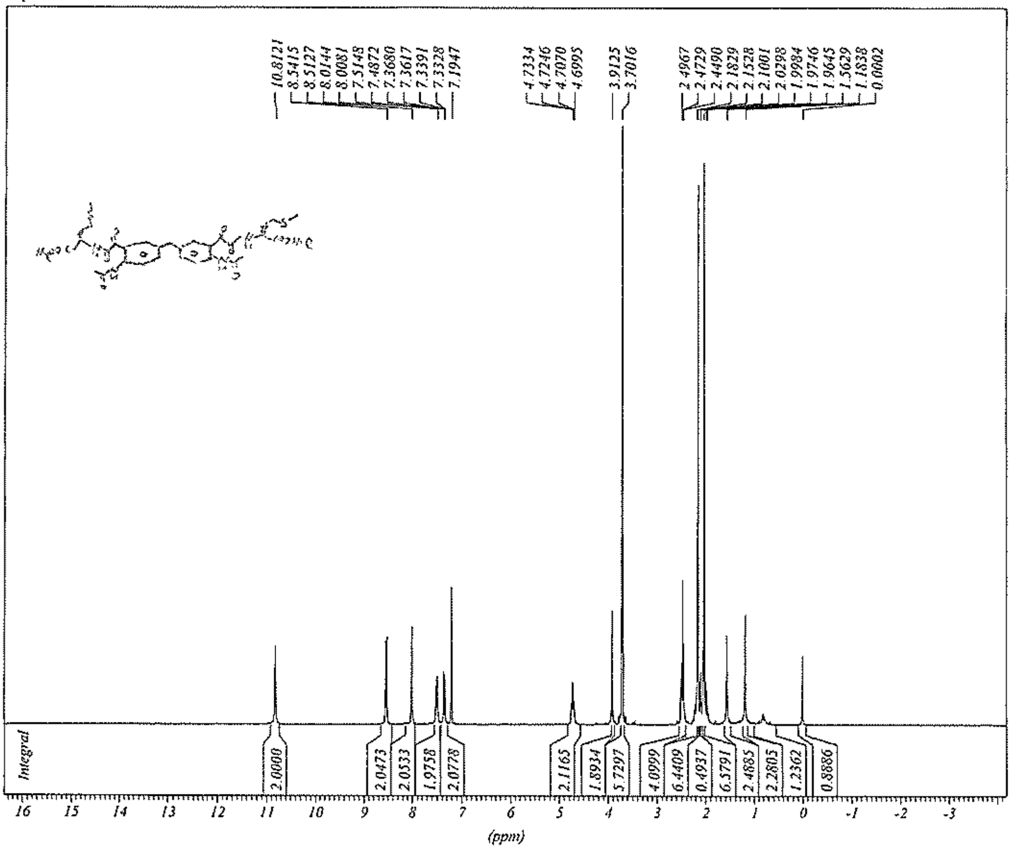

*** Current Data Parameters ***
NAME : 01Z3D8-4
EXPNO : 20
PROCNO : 1

*** Acquisition Parameters ***
DATE_t : 19:33:13
DATE_d : Jul 14 2009
DS : 2
LOCNUC : 2H
NS : 16
O1 : 1853.67 Hz
O2 : 1853.67 Hz
RG : 456.1000061
SFO1 : 300.1718537 MHz
SOLVENT : CDCl3
SW : 20.5644 ppm
TD : 32768
TE : 298.2 K

*** Processing Parameters ***
LB : 0.30 Hz
PC : 1.00
SF : 300.1700248 MHz
SI : 16384

*** 1D NMR Plot Parameters ***
SR : 24.82 Hz
SOLVENT : ?

COMPOUND 23

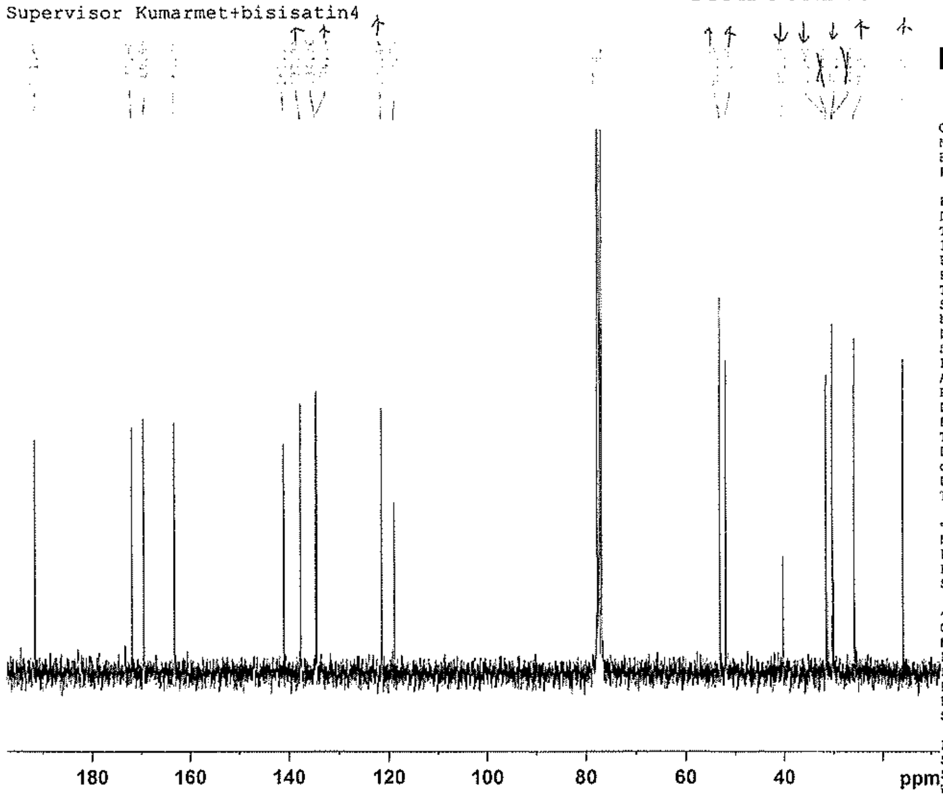

BRUKER

Current Data Parameters
NAME 090716-zrn
EXPNO 41
PROCNO 1

F2 - Acquisition Parameters
Date_ 20090717
Time 7.19
INSTRUM dpx1
PROBHD 5 mm QNP 1H/13
PULPROG zgpg30
TD 65536
SOLVENT CDCl3
NS 2048
DS 2
SWH 23809.523 Hz
FIDRES 0.363304 Hz
AQ 1.3763061 sec
RG 16384
DW 21.000 usec
DE 25.00 usec
TE 300.2 K
D1 2.00000000 sec
d11 0.03000000 sec
DELTA 1.89999998 sec
TD0 1

======== CHANNEL f1 ========
NUC1 13C
P1 7.50 usec
PL1 -3.00 dB
SFO1 75.4873094 MHz

======== CHANNEL f2 ========
CPDPRG2 waltz16
NUC2 1H
PCPD2 105.00 usec
PL2 -3.00 dB
PL12 17.60 dB
PL13 23.60 dB
SFO2 300.1715008 MHz

F2 - Processing parameters
SI 32768
SF 75.4777770 MHz
WDW EM
SSB 0
LB 1.00 Hz
GB 0
PC 1.40

COMPOUND 24

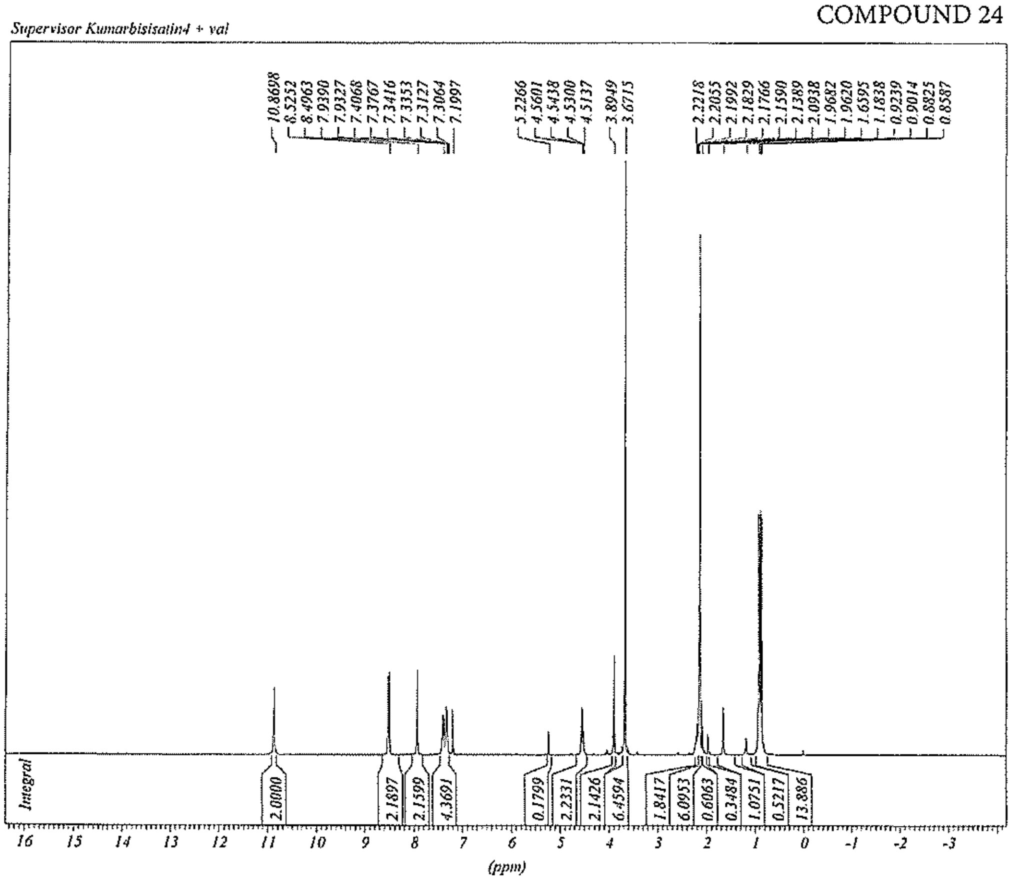

*** Current Data Parameters ***
NAME 0EE514~Q
EXPNO 20
PROCNO 1

*** Acquisition Parameters ***
DATE_t : 19:48:46
DATE_d : Jul 18 2009
DS : 2
LOCNUC : 2H
NS : 16
O1 : 1853.67 Hz
O2 : 1853.67 Hz
RG : 228.1000061
SFO1 : 300.1718537 MHz
SOLVENT : CDCl3
SW : 20.5644 ppm
TD : 32768
TE : 300.2 K

*** Processing Parameters ***
LB : 0.30 Hz
PC : 1.00
SF : 300.1700233 MHz
SI : 16384

*** 1D NMR Plot Parameters ***
SR : 23.33 Hz
SOLVENT : ?

COMPOUND 24

Supervisor Kumarbisisatin4 + val 13C

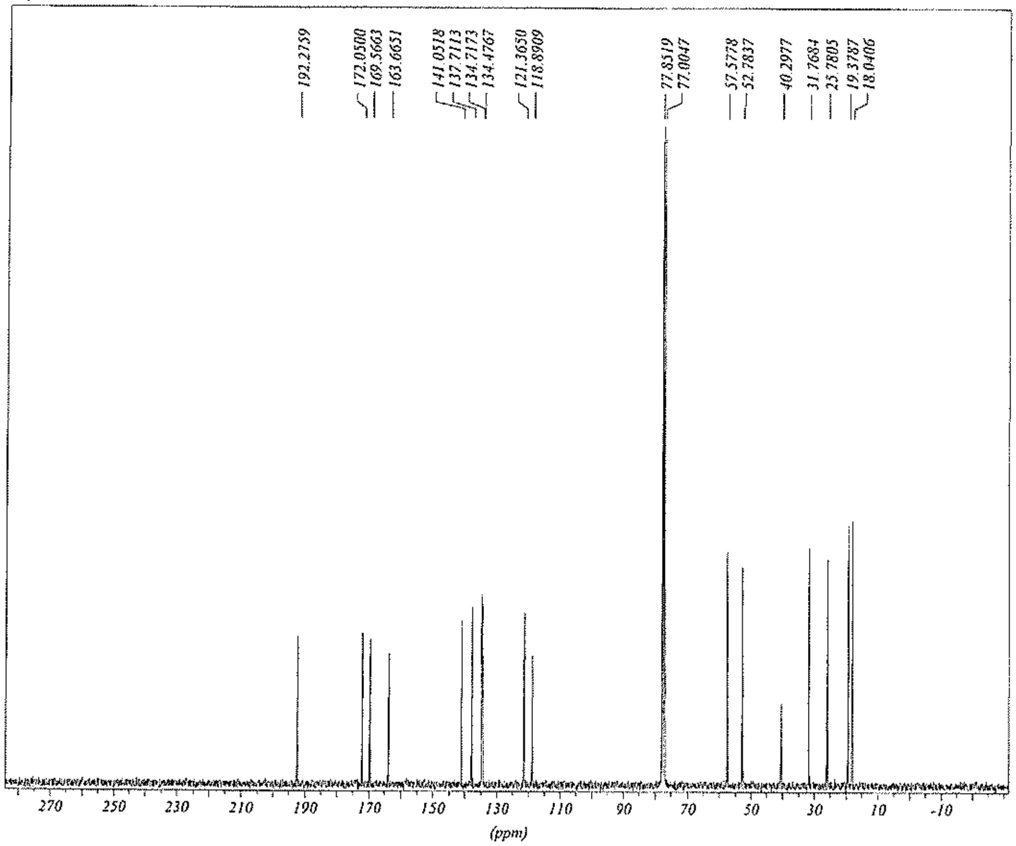

*** Current Data Parameters ***

NAME	:	0EE514~Q
EXPNO	:	21
PROCNO	:	1

*** Acquisition Parameters ***

DATE_t		21:47:01
DATE_d	:	Jul 18 2009
DS	:	2
LOCNUC	:	2H
NS		2048
O1	:	9532.38 Hz
O2	:	1500.85 Hz
RG	:	14596.5000000
SFO1	:	75.4873094 MHz
SOLVENT	:	CDCl3
SW	:	315.4109 ppm
TD	:	65536
TE	:	300.2 K

*** Processing Parameters ***

LB	:	1.00 Hz
PC	:	1.40
SF	:	75.4777770 MHz
SI	:	32768

*** 1D NMR Plot Parameters ***

SR		-0.00 Hz
SOLVENT	:	?

COMPOUND 25

Supervisor KumarD-Phe + bisisatin 4

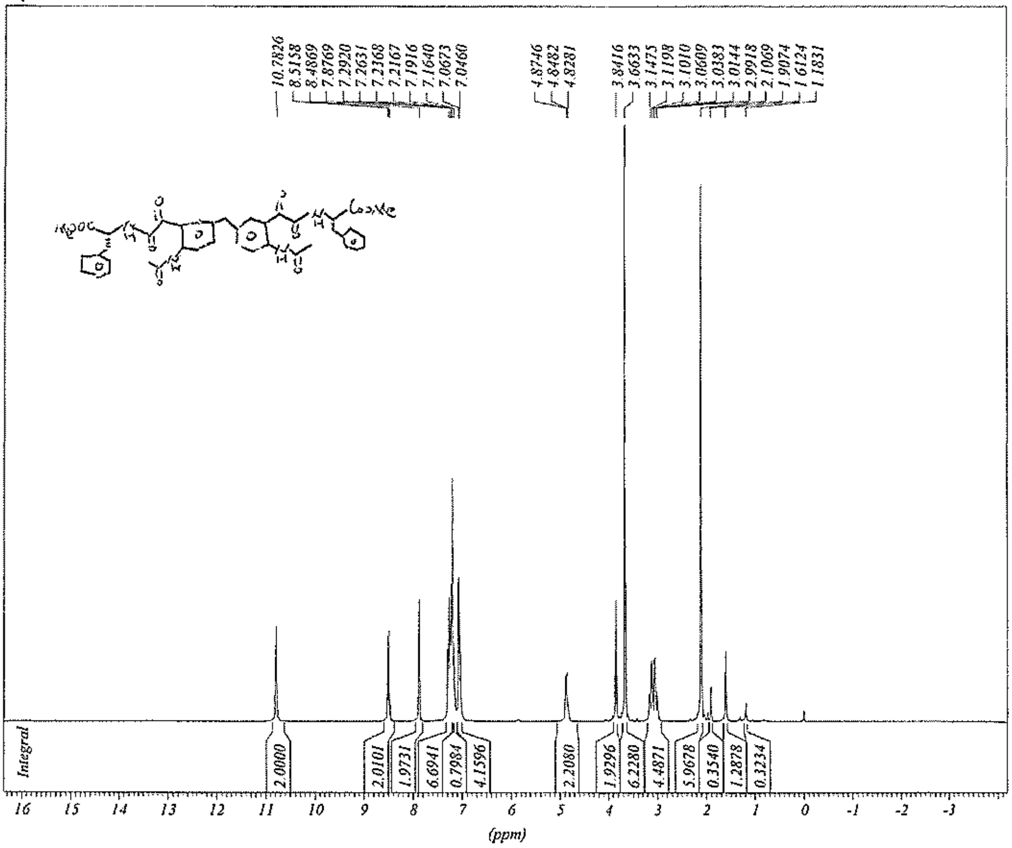

*** Current Data Parameters ***

NAME		01Z3D8~4
EXPNO	:	40
PROCNO		1

*** Acquisition Parameters ***

DATE_t		18:16:48
DATE_d		Jul 15 2009
DS		2
LOCNUC	:	2H
NS		16
O1		1853.67 Hz
O2		1853.67 Hz
RG	:	256.0000000
SFO1	:	300.1718537 MHz
SOLVENT	:	CDCl3
SW	:	20.5644 ppm
TD		32768
TE		298.2 K

*** Processing Parameters ***

LB	:	0.30 Hz
PC	:	1.00
SF		300.1700265 MHz
SI		16384

*** 1D NMR Plot Parameters ***

SR	:	26.53 Hz
SOLVENT	:	?

COMPOUND 27

Supervisor Kumarbisisatin 5+gly

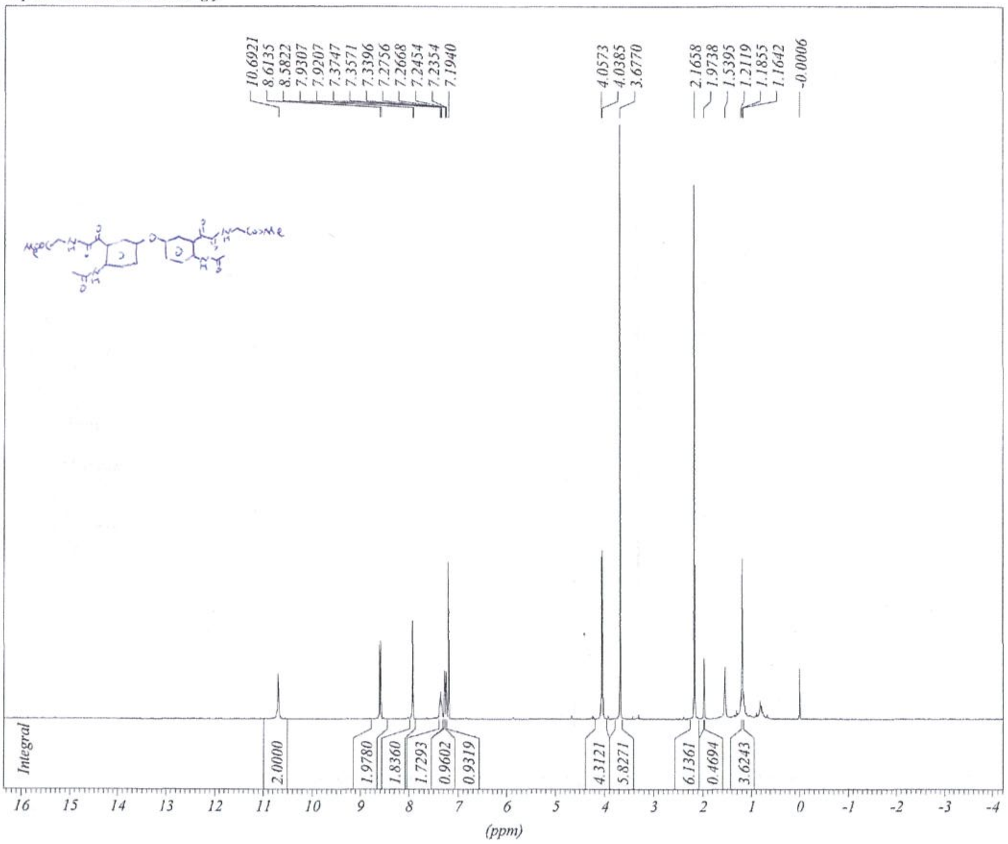

*** Current Data Parameters ***

NAME	:	0G8YST~9
EXPNO	:	20
PROCNO	:	1

*** Acquisition Parameters ***

DATE_t	:	14:37:03
DATE_d	:	Sep 23 2009
DS	:	2
LOCNUC	:	2H
NS	:	16
O1	:	1853.67 Hz
O2	:	1853.67 Hz
RG	:	574.7000122
SFO1	:	300.1718537 MHz
SOLVENT	:	CDCl3
SW	:	20.5644 ppm
TD	:	32768
TE	:	300.2 K

*** Processing Parameters ***

LB	:	0.30 Hz
PC	:	1.00
SF	:	300.1700315 MHz
SI	:	16384

*** 1D NMR Plot Parameters ***

SR	:	31.46 Hz
SOLVENT	:	?

32

COMPOUND 27

Supervisor Kumarbisisatin 5+gly

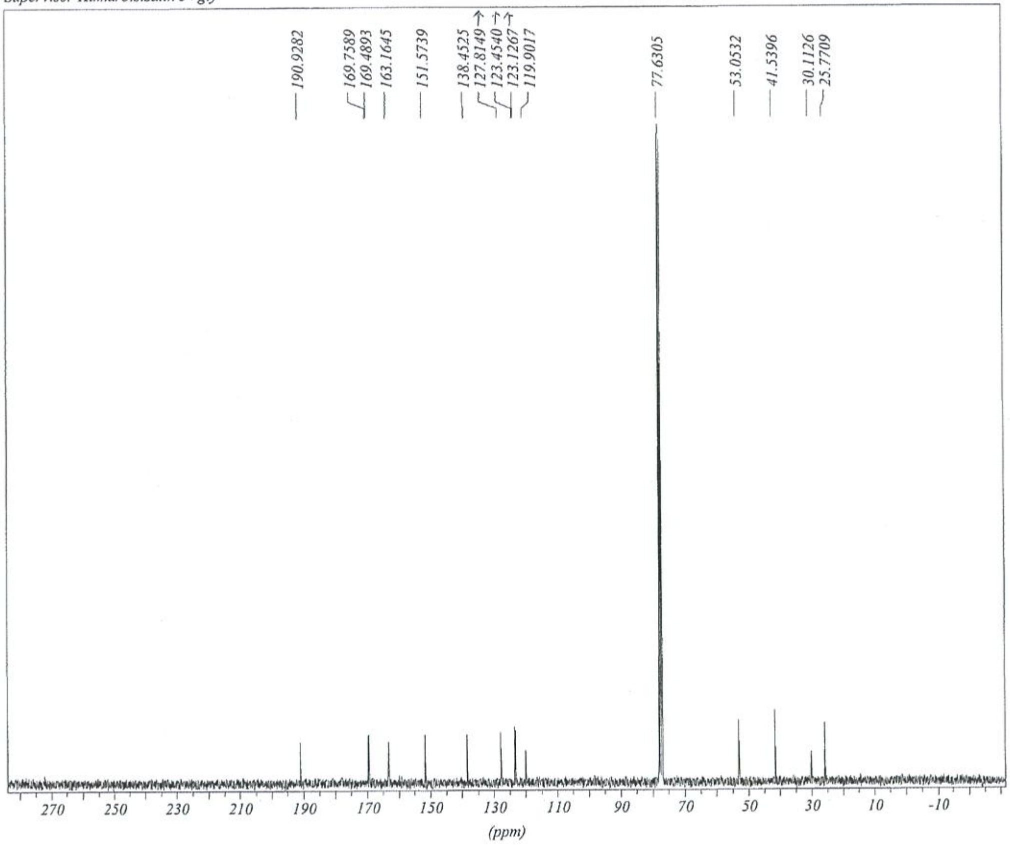

*** Current Data Parameters ***

NAME	:	0G8YST~9
EXPNO	:	21
PROCNO	:	1

*** Acquisition Parameters ***

DATE_t	:	16:05:59
DATE_d	:	Sep 23 2009
DS	:	2
LOCNUC	:	2H
NS	:	1536
O1	:	9532.38 Hz
O2	:	1500.85 Hz
RG	:	16384.0000000
SFO1	:	75.4873094 MHz
SOLVENT	:	CDCl3
SW	:	315.4109 ppm
TD	:	65536
TE	:	300.2 K

*** Processing Parameters ***

LB	:	1.00 Hz
PC	:	1.40
SF	:	75.4777770 MHz
SI	:	32768

*** 1D NMR Plot Parameters ***

SR	:	-0.00 Hz
SOLVENT	:	?

Supervisor Kumar
bisisatin 5+ leu
1H CDCl3 F:\\ zrn 23

COMPOUND 28

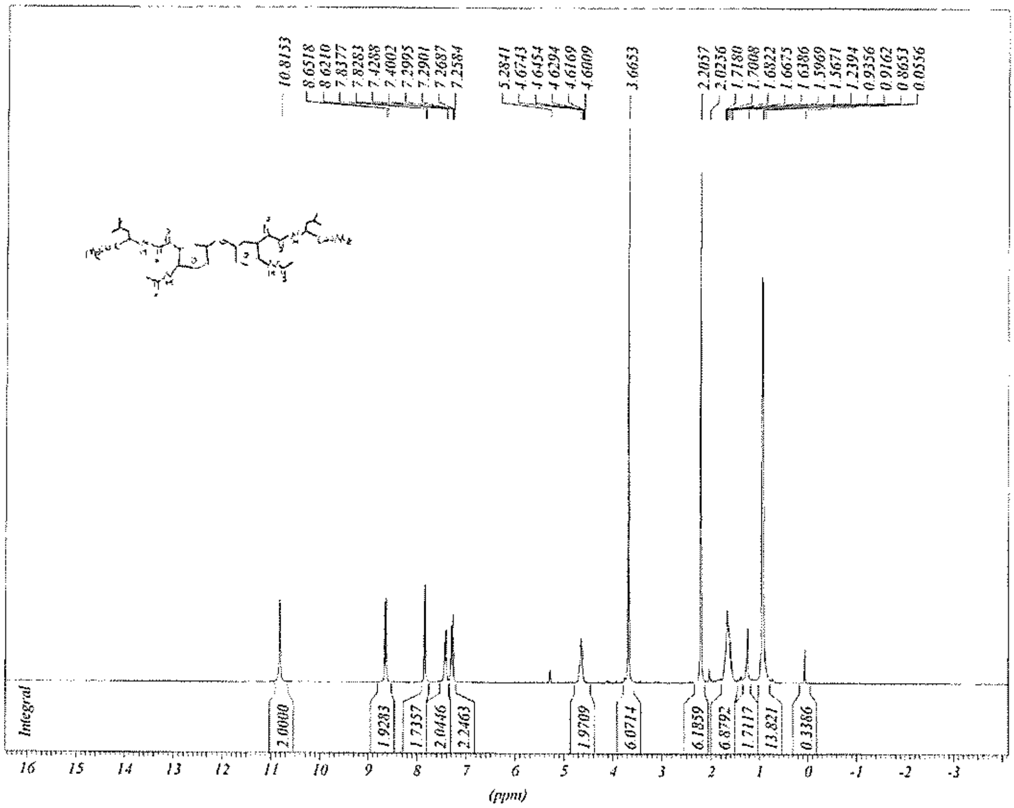

*** Current Data Parameters ***

NAME	:	06WLV8-W
EXPNO	:	1
PROCNO	:	1

*** Acquisition Parameters ***

DATE_t		01:45:26
DATE_d	:	Oct 15 2009
DS		4
LOCNUC	:	2H
NS		16
O1		1853.67 Hz
O2		0.00 Hz
RG		71.8000031
SFO1	:	300.1318537 MHz
SOLVENT		CDCl3
SW	:	20.5671 ppm
TD		32768
TE		297.5 K

*** Processing Parameters ***

LB	:	0.30 Hz
PC		1.00
SF	:	300.1300071 MHz
SI	:	65536

*** 1D NMR Plot Parameters ***

SR	:	7.14 Hz
SOLVENT	:	?

32

Supervisor Kumar
bisisatin 5+ leu
13C CDCl3 F:\\ zrn 23

COMPOUND 28

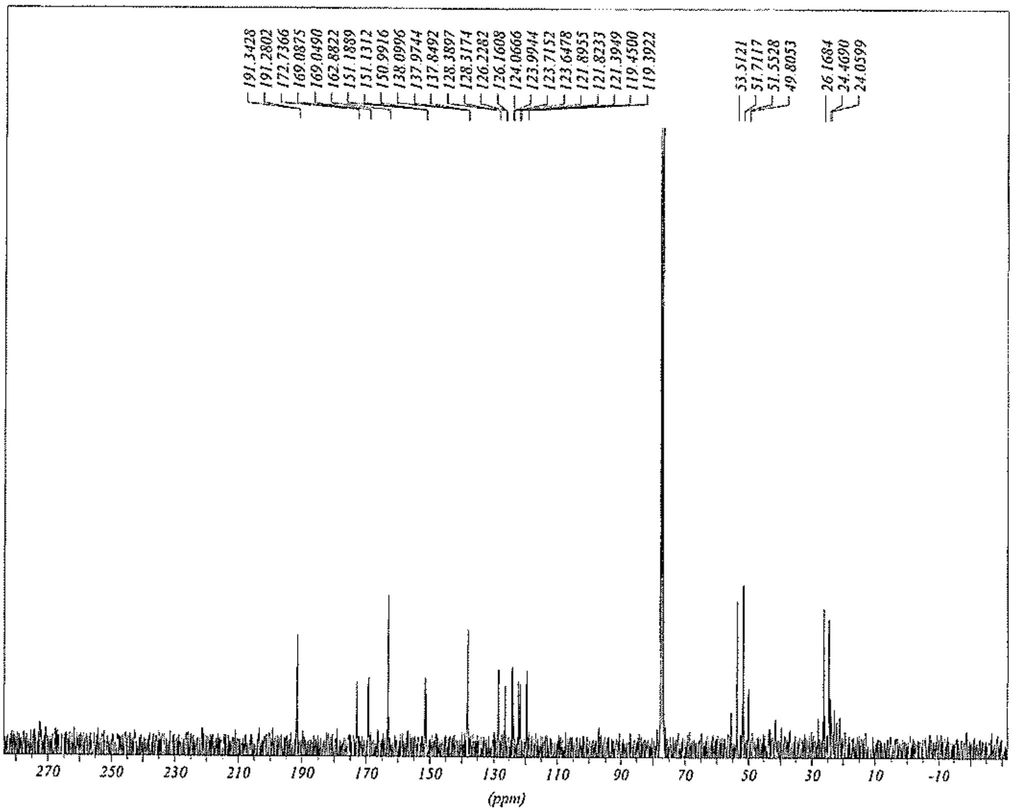

*** Current Data Parameters ***

NAME	:	06WLV8-W
EXPNO	:	2
PROCNO	:	1

*** Acquisition Parameters ***

DATE_t	:	03:43:09
DATE_d	:	Oct 15 2009
DS	:	0
LOCNUC	:	2H
NS	:	2048
O1	:	9502.00 Hz
O2	:	0.00 Hz
RG	:	20642.5000000
SFO1	:	75.4772510 MHz
SOLVENT	:	CDCl3
SW	:	315.4530 ppm
TD	:	65536
TE	:	298.4 K

*** Processing Parameters ***

LB	:	1.00 Hz
PC	:	1.40
SF	:	75.4677485 MHz
SI	:	65536

*** 1D NMR Plot Parameters ***

SR	:	-0.54 Hz
SOLVENT	:	?

Supervisor Kumar
met+bisisatin 5
1H CDCl3 F:\\ zrn 19

COMPOUND 29

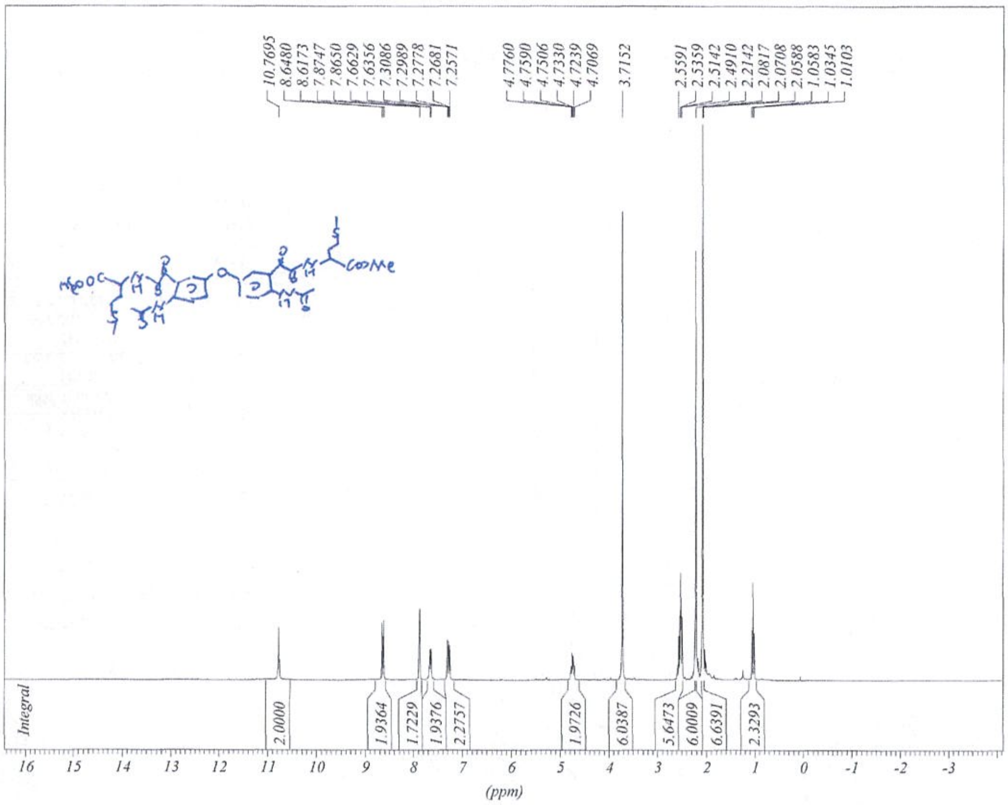

*** Current Data Parameters ***

NAME : 0JSD3M~4
EXPNO : 4
PROCNO : 1

*** Acquisition Parameters ***

DATE_t : 13:36:42
DATE_d : Oct 19 2009
DS : 4
LOCNUC : 2H
NS : 16
O1 : 1853.67 Hz
O2 : 0.00 Hz
RG : 128.0000000
SFO1 : 300.1318537 MHz
SOLVENT : CDCl3
SW : 20.5671 ppm
TD : 32768
TE : 297.8 K

*** Processing Parameters ***

LB : 0.30 Hz
PC : 1.00
SF : 300.1300071 MHz
SI : 65536

*** 1D NMR Plot Parameters ***

SR : 7.14 Hz
SOLVENT : ?

39

Supervisor Kumar
met+bisisatin 5
13C{1H} CDCl3 F:\\ zrn 19

COMPOUND 29

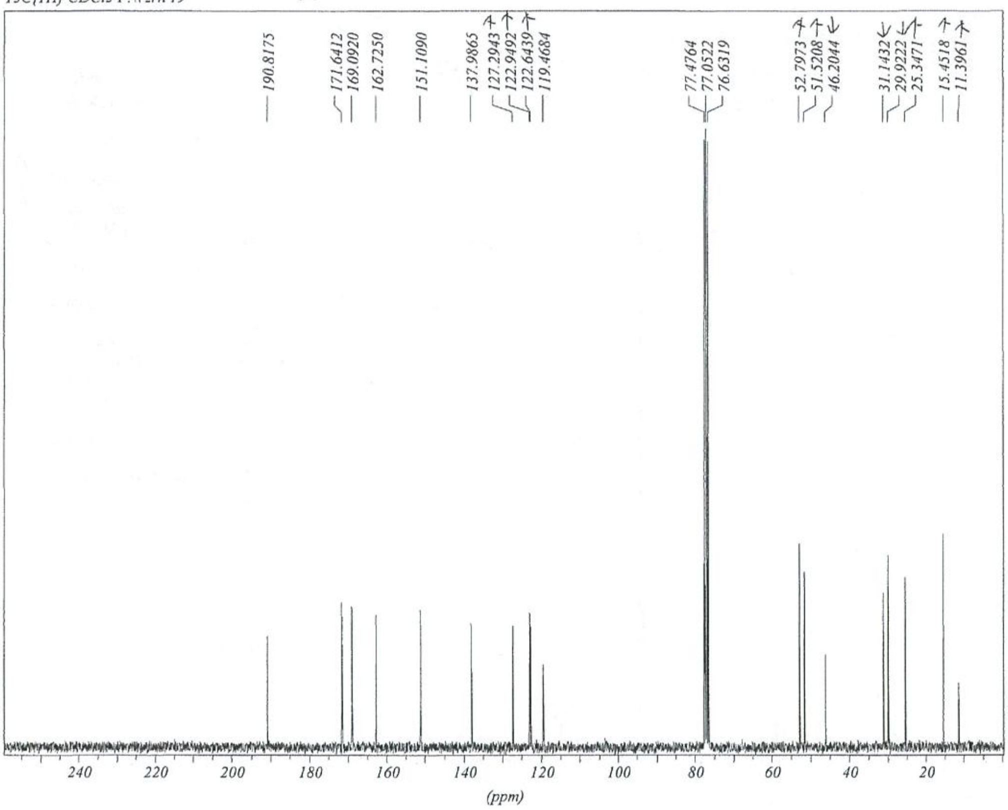

*** Current Data Parameters ***

NAME : 0JSD3M~4
EXPNO : 5
PROCNO : 1

*** Acquisition Parameters ***

DATE_t : 14:42:15
DATE_d : Oct 19 2009
DS : 0
LOCNUC : 2H
NS : 1024
O1 : 9810.80 Hz
O2 : 1500.85 Hz
RG : 18390.4003906
SFO1 : 75.4775298 MHz
SOLVENT : CDCl3
SW : 259.7838 ppm
TD : 65536
TE : 298.1 K

*** Processing Parameters ***

LB : 1.00 Hz
PC : 1.40
SF : 75.4677485 MHz
SI : 65536

*** 1D NMR Plot Parameters ***

SR : 29.46 Hz
SOLVENT : ?

Supervisor Kumar
D-phe+bisisatin5
1H CDCl3 F:\\ zrn 19

COMPOUND **30**

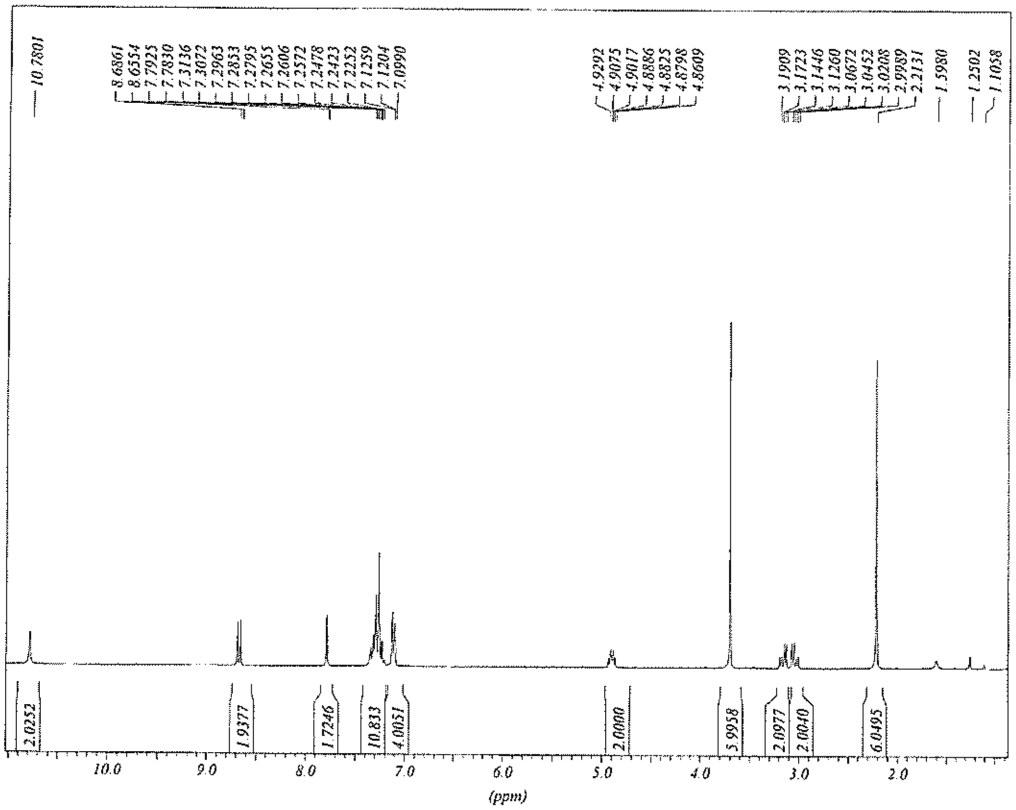

*** Current Data Parameters ***
NAME : 0UZEN5~A
EXPNO : 1
PROCNO : 1

*** Acquisition Parameters ***
DATE_t : 01:30:07
DATE_d : Oct 26 2009
DS : 0
LOCNUC : 2H
NS : 16
O1 : 2100.91 Hz
O2 : 0.00 Hz
RG : 322.5000000
SFO1 : 300.1321009 MHz
SOLVENT : CDCl3
SW : 19.9752 ppm
TD : 32768
TE : 298.3 K

*** Processing Parameters ***
LB : 0.30 Hz
PC : 1.00
SF : 300.1300071 MHz
SI : 65536

*** 1D NMR Plot Parameters ***
SR : 7.14 Hz
SOLVENT : ?

35

Supervisor Kumar
D-phe+bisisatin5
13C{1H} CDCl3 F:\\ zrn 19

COMPOUND **30**

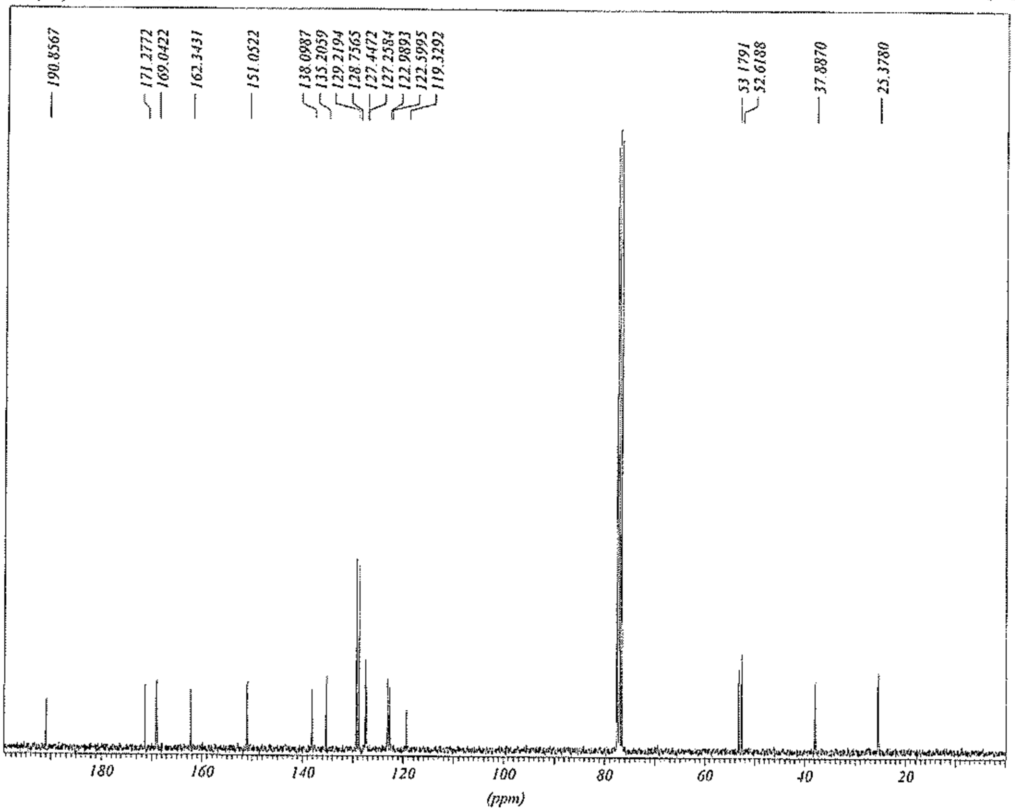

*** Current Data Parameters ***
NAME : 0UZEN5~A
EXPNO : 2
PROCNO : 1

*** Acquisition Parameters ***
DATE_t : 03:19:21
DATE_d : Oct 26 2009
DS : 0
LOCNUC : 2H
NS : 2048
O1 : 7546.77 Hz
O2 : 1500.85 Hz
RG : 14596.5000000
SFO1 : 75.4752658 MHz
SOLVENT : CDCl3
SW : 199.5388 ppm
TD : 32768
TE : 298.3 K

*** Processing Parameters ***
LB : 1.00 Hz
PC : 1.40
SF : 75.4677485 MHz
SI : 32768

*** 1D NMR Plot Parameters ***
SR : 29.46 Hz
SOLVENT : ?

Supervisor Kumarbisisatin5+val

COMPOUND 3[illegible]

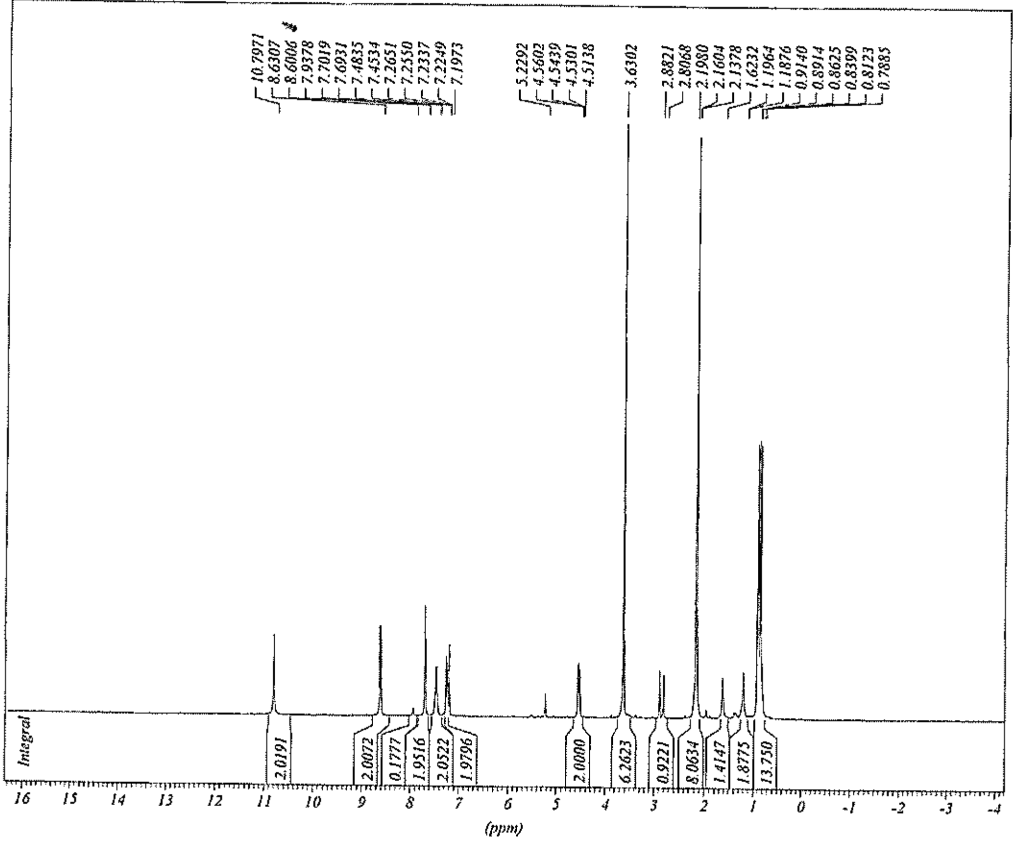

*** Current Data Parameters ***
NAME : 0MFJ4O-Y
EXPNO : 10
PROCNO : 1

*** Acquisition Parameters ***
DATE_t : 12:57:52
DATE_d : Sep 15 2009
DS : 2
LOCNUC : 2H
NS : 16
O1 : 1853.67 Hz
O2 : 1853.67 Hz
RG : 322.5000000
SFO1 : 300.1718537 MHz
SOLVENT : CDCl3
SW : 20.5644 ppm
TD : 32768
TE : 300.2 K

*** Processing Parameters ***
LB : 0.30 Hz
PC : 1.00
SF : 300.1700305 MHz
SI : 16384

*** 1D NMR Plot Parameters ***
SR : 30.46 Hz
SOLVENT : ?

Supervisor Kumarbisisatin5+val

COMPOUND 3[illegible]

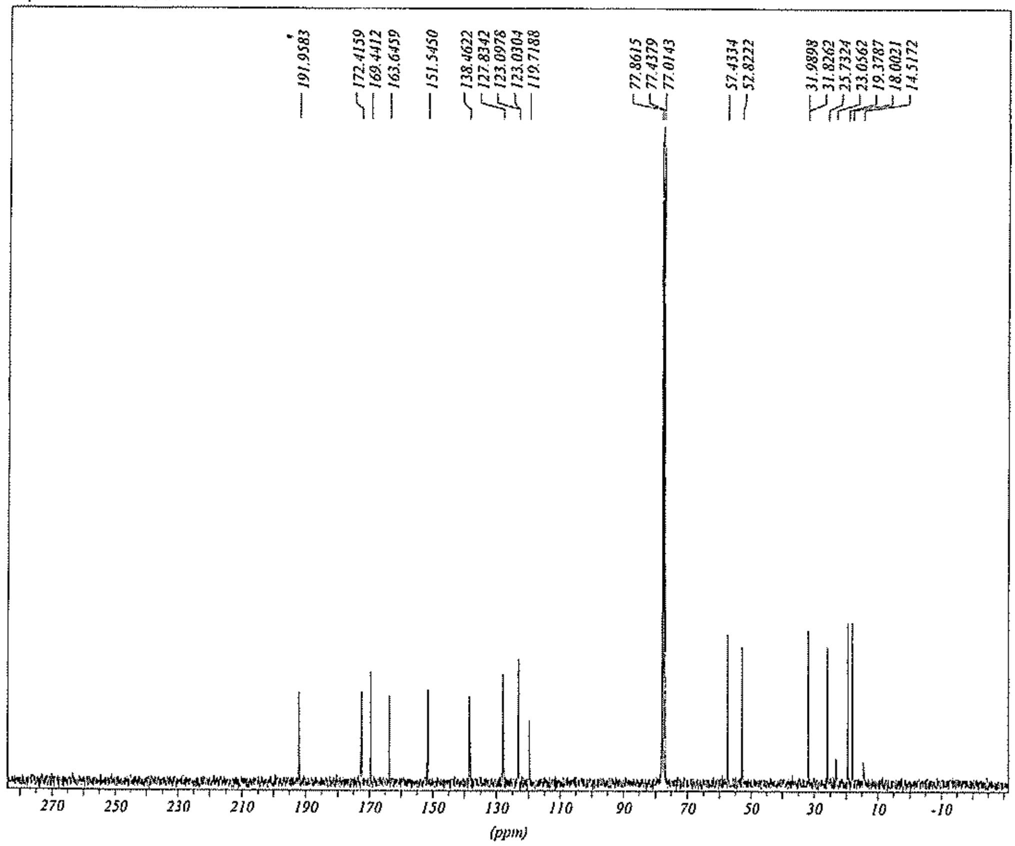

*** Current Data Parameters ***
NAME : 0MFJ4O-Y
EXPNO : 11
PROCNO : 1

*** Acquisition Parameters ***
DATE_t : 14:26:48
DATE_d : Sep 15 2009
DS : 2
LOCNUC : 2H
NS : 1536
O1 : 9532.38 Hz
O2 : 1500.85 Hz
RG : 16384.0000000
SFO1 : 75.4873094 MHz
SOLVENT : CDCl3
SW : 315.4109 ppm
TD : 65536
TE : 300.2 K

*** Processing Parameters ***
LB : 1.00 Hz
PC : 1.40
SF : 75.4777770 MHz
SI : 32768

*** 1D NMR Plot Parameters ***
SR : -0.00 Hz
SOLVENT : ?

COMPOUND 26

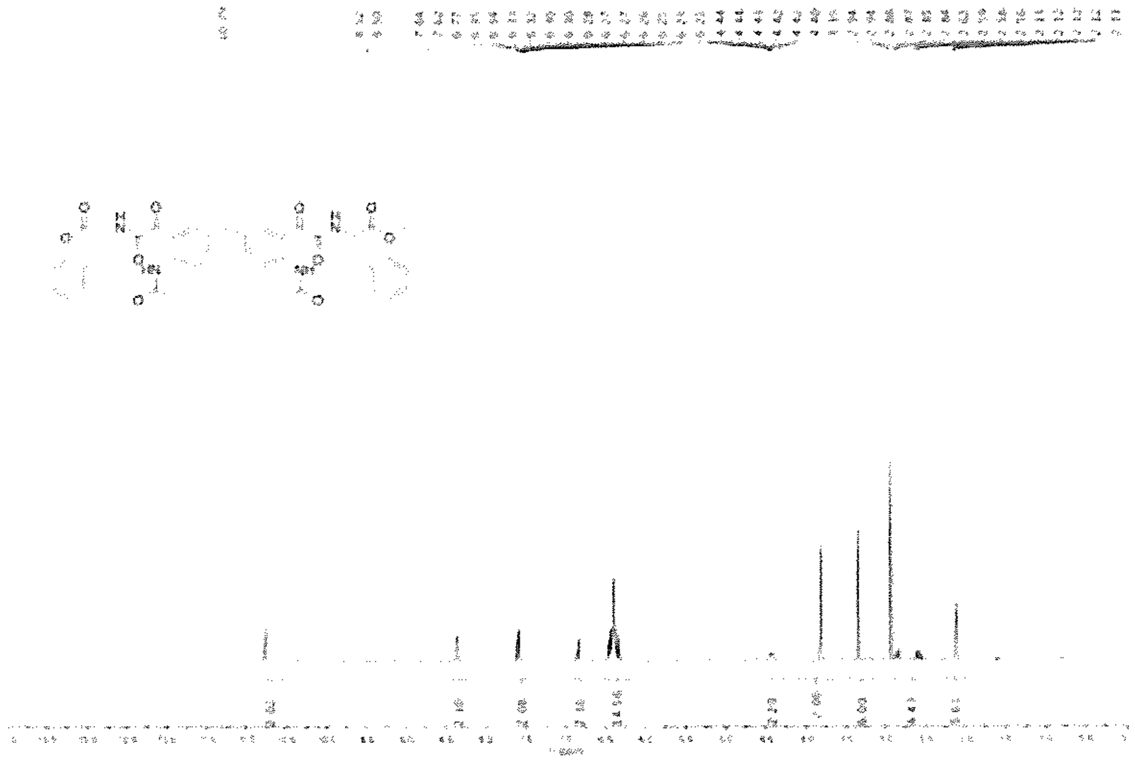

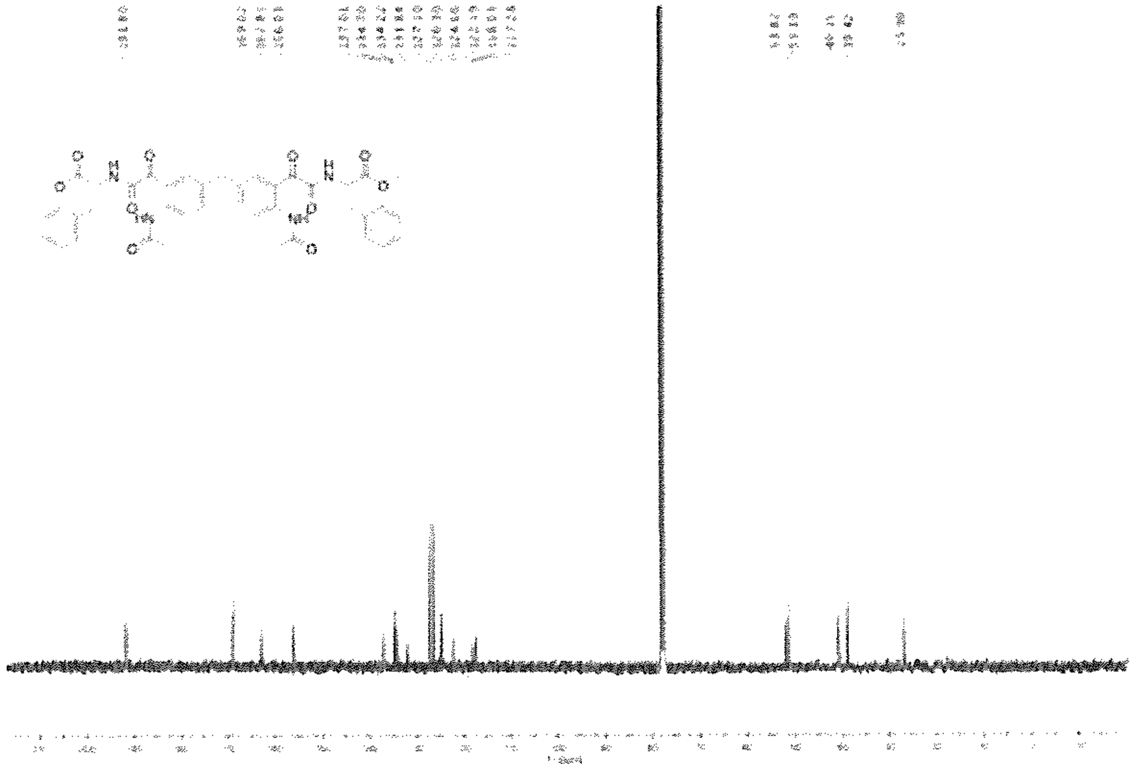

COMPOUND 32

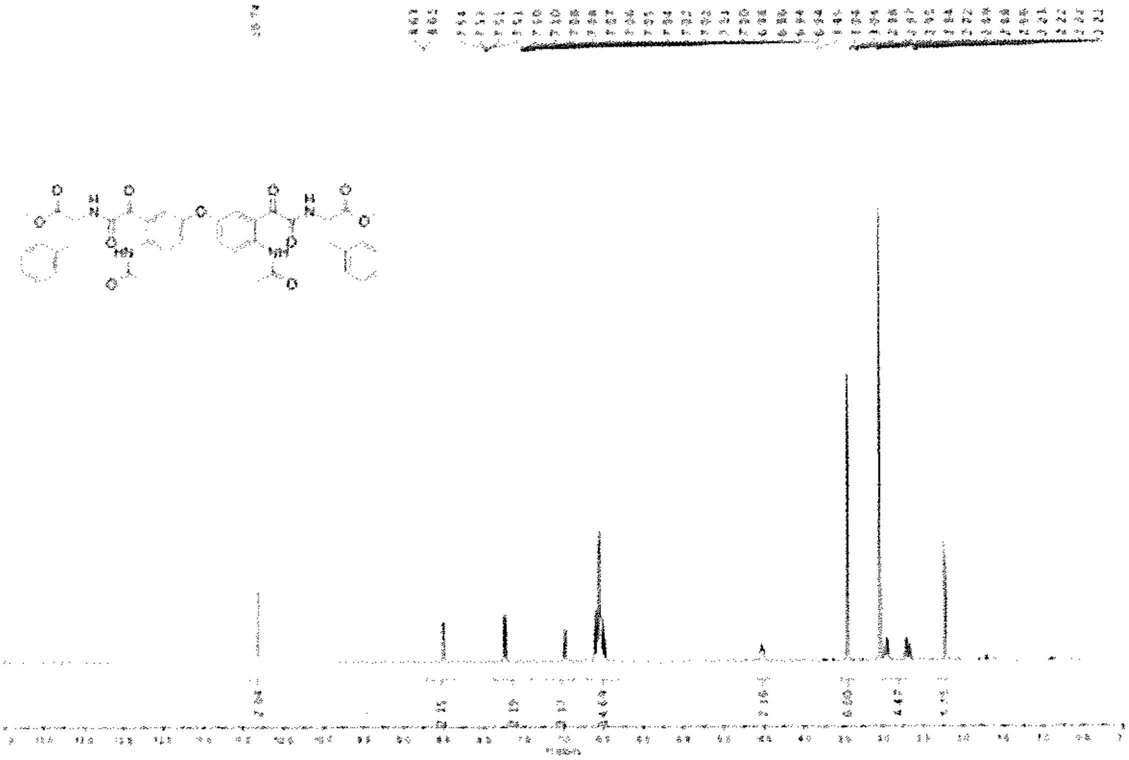

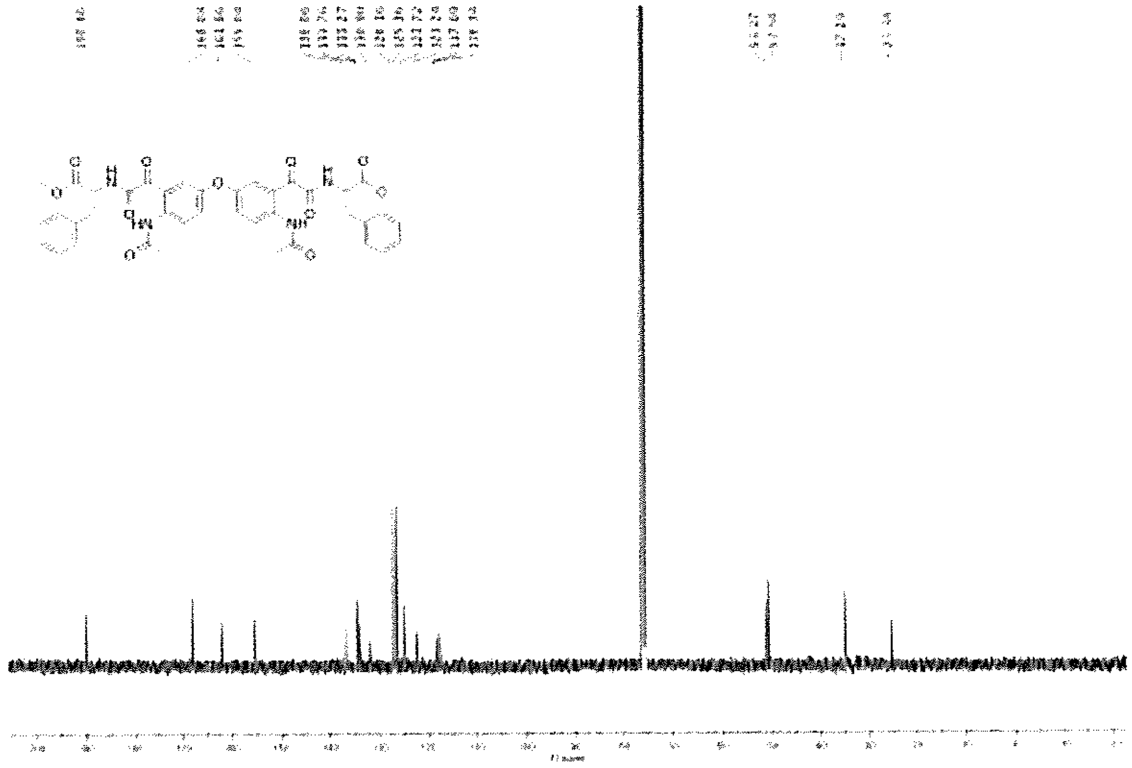

checkCIF/PLATON report

Structure factors have been supplied for datablock(s) vs3arepro

THIS REPORT IS FOR GUIDANCE ONLY. IF USED AS PART OF A REVIEW PROCEDURE FOR PUBLICATION, IT SHOULD NOT REPLACE THE EXPERTISE OF AN EXPERIENCED CRYSTALLOGRAPHIC REFEREE.

No syntax errors found. CIF dictionary Interpreting this report

Datablock: vs3arepro

```
Bond precision:  C-C = 0.0150 A          Wavelength=0.71073

Cell:               a=23.015(5)      b=31.326(6)      c=4.984(1)
                    alpha=90         beta=90          gamma=90
Temperature:        100 K

                Calculated                  Reported
Volume          3593.3(13)                  3593.3(12)
Space group     P 21 21 2                   P 21 21 2
Hall group      P 2 2ab                     P 2 2ab
Moiety formula  C35 H42 N4 O10 [+ solvent]  C35 H42 N4 O10
Sum formula     C35 H42 N4 O10 [+ solvent]  C35 H42 N4 O10
Mr              678.73                      678.72
Dx,g cm-3       1.255                       1.255
Z               4                           4
Mu (mm-1)       0.093                       0.093
F000            1440.0                      1440.0
F000'           1440.75
h,k,lmax        26,35,5                     26,35,5
Nref            5588[ 3284]                 5471
Tmin,Tmax       0.999,0.999
Tmin'           0.999

Correction method= Not given

Data completeness= 1.67/0.98      Theta(max)= 23.885

R(reflections)= 0.1380( 4913)     wR2(reflections)= 0.3602( 5471)

S = 1.571                   Npar= 446
```

The following ALERTS were generated. Each ALERT has the format
test-name_ALERT_alert-type_alert-level.
Click on the hyperlinks for more details of the test.

Alert level B

```
THETM01_ALERT_3_B  The value of sine(theta_max)/wavelength is less than 0.575
            Calculated sin(theta_max)/wavelength =    0.5697
PLAT084_ALERT_3_B High wR2 Value (i.e. > 0.25) ...................       0.36 Report
```

Author Response: Crystals were ultra thin and could be only measured for diffraction at Australian Synchrotron. Even with the synchrotron radiation they diffracted only to lower angles. So, the alerts arising at level B below are due to weak diffraction, disordered L-leu moities and possible presence of partially occupied water molecule in the crystal lattice.

```
PLAT097_ALERT_2_B Large Reported Max.  (Positive) Residual Density       0.81 eA-3
```

Author Response: as explained above

```
PLAT340_ALERT_3_B Low Bond Precision on  C-C Bonds ...............    0.01504 Ang.
```

Author Response: as explained above

```
PLAT369_ALERT_2_B Long   C(sp2)-C(sp2) Bond  C9B      - C10B     .       1.57 Ang.
```

Author Response: as explained above

Alert level C

```
DIFMX02_ALERT_1_C  The maximum difference density is > 0.1*ZMAX*0.75
            The relevant atom site should be identified.
RINTA01_ALERT_3_C  The value of Rint is greater than 0.12
            Rint given   0.120
STRVA01_ALERT_4_C           Flack test results are meaningless.
           From the CIF: _refine_ls_abs_structure_Flack    0.300
           From the CIF: _refine_ls_abs_structure_Flack_su    0.700
PLAT082_ALERT_2_C High R1 Value ..................................       0.14 Report
PLAT089_ALERT_3_C Poor Data / Parameter Ratio (Zmax < 18) ........       7.23 Note
PLAT213_ALERT_2_C Atom C13B            has ADP max/min Ratio .....        3.3 prolat
PLAT220_ALERT_2_C Non-Solvent  Resd 1  C   Ueq(max)/Ueq(min) Range        5.1 Ratio
PLAT222_ALERT_3_C Non-Solv.  Resd 1  H   Uiso(max)/Uiso(min) Range        6.4 Ratio
PLAT234_ALERT_4_C Large Hirshfeld Difference O3A      --C10A     .       0.16 Ang.
PLAT234_ALERT_4_C Large Hirshfeld Difference O4A      --C12A     .       0.17 Ang.
PLAT234_ALERT_4_C Large Hirshfeld Difference C5A      --C6A      .       0.16 Ang.
PLAT234_ALERT_4_C Large Hirshfeld Difference C11A     --C12A     .       0.19 Ang.
PLAT241_ALERT_2_C High   'MainMol' Ueq as Compared to Neighbors of        C7B Check
PLAT241_ALERT_2_C High   'MainMol' Ueq as Compared to Neighbors of       C14A Check
PLAT242_ALERT_2_C Low    'MainMol' Ueq as Compared to Neighbors of        O5B Check
PLAT242_ALERT_2_C Low    'MainMol' Ueq as Compared to Neighbors of        N1B Check
PLAT242_ALERT_2_C Low    'MainMol' Ueq as Compared to Neighbors of        C7A Check
PLAT242_ALERT_2_C Low    'MainMol' Ueq as Compared to Neighbors of       C11A Check
PLAT329_ALERT_4_C Carbon Atom Hybridisation Unclear for ..........       C14B Check
PLAT369_ALERT_2_C Long   C(sp2)-C(sp2) Bond  C9A      - C10A     .       1.53 Ang.
```

Author Response: as explained above

```
PLAT906_ALERT_3_C Large K Value in the Analysis of Variance ......      2.950 Check
PLAT911_ALERT_3_C Missing FCF Refl Between Thmin & STh/L=    0.570         55 Report
PLAT913_ALERT_3_C Missing # of Very Strong Reflections in FCF ....          7 Note
PLAT934_ALERT_3_C Number of (Iobs-Icalc)/Sigma(W) > 10 Outliers ..          1 Check
PLAT975_ALERT_2_C Check Calcd Resid. Dens.  0.90A   From O3A             0.78 eA-3
PLAT977_ALERT_2_C Check Negative Difference Density on H15B             -0.31 eA-3
PLAT978_ALERT_2_C Number C-C Bonds with Positive Residual Density.          0 Info
```

Alert level G

```
PLAT002_ALERT_2_G Number of Distance or Angle Restraints on AtSite         13 Note
PLAT003_ALERT_2_G Number of Uiso or Uij Restrained non-H Atoms ...          4 Report
PLAT007_ALERT_5_G Number of Unrefined Donor-H Atoms .............           4 Report
PLAT032_ALERT_4_G Std. Uncertainty on Flack Parameter Value High .      0.700 Report
PLAT072_ALERT_2_G SHELXL First  Parameter in WGHT  Unusually Large       0.20 Report
PLAT171_ALERT_4_G The CIF-Embedded .res File Contains EADP Records          9 Report
PLAT172_ALERT_4_G The CIF-Embedded .res File Contains DFIX Records         17 Report
PLAT176_ALERT_4_G The CIF-Embedded .res File Contains SADI Records          1 Report
PLAT187_ALERT_4_G The CIF-Embedded .res File Contains RIGU Records          3 Report
PLAT300_ALERT_4_G Atom Site Occupancy of C15'       Constrained at        0.5 Check
PLAT300_ALERT_4_G Atom Site Occupancy of C15B       Constrained at        0.5 Check
PLAT300_ALERT_4_G Atom Site Occupancy of C16'       Constrained at        0.5 Check
PLAT300_ALERT_4_G Atom Site Occupancy of C16"       Constrained at        0.5 Check
PLAT300_ALERT_4_G Atom Site Occupancy of C16A       Constrained at        0.5 Check
PLAT300_ALERT_4_G Atom Site Occupancy of C16B       Constrained at        0.5 Check
PLAT300_ALERT_4_G Atom Site Occupancy of C17'       Constrained at        0.5 Check
PLAT300_ALERT_4_G Atom Site Occupancy of C17"       Constrained at        0.5 Check
PLAT300_ALERT_4_G Atom Site Occupancy of C17A       Constrained at        0.5 Check
PLAT300_ALERT_4_G Atom Site Occupancy of C17B       Constrained at        0.5 Check
PLAT300_ALERT_4_G Atom Site Occupancy of H14C       Constrained at        0.5 Check
PLAT300_ALERT_4_G Atom Site Occupancy of H14D       Constrained at        0.5 Check
PLAT300_ALERT_4_G Atom Site Occupancy of H15'       Constrained at        0.5 Check
PLAT300_ALERT_4_G Atom Site Occupancy of H15A       Constrained at        0.5 Check
PLAT300_ALERT_4_G Atom Site Occupancy of H15B       Constrained at        0.5 Check
PLAT300_ALERT_4_G Atom Site Occupancy of H15C       Constrained at        0.5 Check
PLAT300_ALERT_4_G Atom Site Occupancy of H16A       Constrained at        0.5 Check
PLAT300_ALERT_4_G Atom Site Occupancy of H16B       Constrained at        0.5 Check
PLAT300_ALERT_4_G Atom Site Occupancy of H16C       Constrained at        0.5 Check
PLAT300_ALERT_4_G Atom Site Occupancy of H16D       Constrained at        0.5 Check
PLAT300_ALERT_4_G Atom Site Occupancy of H16E       Constrained at        0.5 Check
PLAT300_ALERT_4_G Atom Site Occupancy of H16F       Constrained at        0.5 Check
PLAT300_ALERT_4_G Atom Site Occupancy of H16G       Constrained at        0.5 Check
PLAT300_ALERT_4_G Atom Site Occupancy of H16H       Constrained at        0.5 Check
PLAT300_ALERT_4_G Atom Site Occupancy of H16I       Constrained at        0.5 Check
PLAT300_ALERT_4_G Atom Site Occupancy of H16J       Constrained at        0.5 Check
PLAT300_ALERT_4_G Atom Site Occupancy of H16K       Constrained at        0.5 Check
PLAT300_ALERT_4_G Atom Site Occupancy of H16L       Constrained at        0.5 Check
PLAT300_ALERT_4_G Atom Site Occupancy of H17A       Constrained at        0.5 Check
PLAT300_ALERT_4_G Atom Site Occupancy of H17B       Constrained at        0.5 Check
PLAT300_ALERT_4_G Atom Site Occupancy of H17C       Constrained at        0.5 Check
PLAT300_ALERT_4_G Atom Site Occupancy of H17D       Constrained at        0.5 Check
PLAT300_ALERT_4_G Atom Site Occupancy of H17E       Constrained at        0.5 Check
PLAT300_ALERT_4_G Atom Site Occupancy of H17F       Constrained at        0.5 Check
PLAT300_ALERT_4_G Atom Site Occupancy of H17G       Constrained at        0.5 Check
PLAT300_ALERT_4_G Atom Site Occupancy of H17H       Constrained at        0.5 Check
PLAT300_ALERT_4_G Atom Site Occupancy of H17I       Constrained at        0.5 Check
PLAT300_ALERT_4_G Atom Site Occupancy of H17J       Constrained at        0.5 Check
PLAT300_ALERT_4_G Atom Site Occupancy of H17K       Constrained at        0.5 Check
```

```
PLAT300_ALERT_4_G Atom Site Occupancy of H17L       Constrained at        0.5 Check
PLAT301_ALERT_3_G Main Residue  Disorder ..............(Resd  1  )        10% Note
PLAT343_ALERT_2_G Unusual       sp?Angle Range in Main Residue for       C11A Check
PLAT367_ALERT_2_G Long?  C(sp?)-C(sp?) Bond  C11A     - C12A     .       1.56 Ang.
PLAT367_ALERT_2_G Long?  C(sp?)-C(sp?) Bond  C11A     - C14A     .       1.56 Ang.
PLAT412_ALERT_2_G Short Intra XH3 .. XHn     H14B     ..H17C     .       2.09 Ang.
                                                    x,y,z  =     1_555 Check
PLAT413_ALERT_2_G Short Inter XH3 .. XHn     H14A     ..H17K     .       2.14 Ang.
                                                  x,y,1+z  =     1_556 Check
PLAT432_ALERT_2_G Short Inter X...Y Contact  O3A      ..C12A             2.88 Ang.
                                                 x,y,-1+z  =     1_554 Check
PLAT605_ALERT_4_G Largest Solvent Accessible VOID in the Structure         56 A**3
PLAT720_ALERT_4_G Number of Unusual/Non-Standard Labels ..........          8 Note
PLAT791_ALERT_4_G Model has Chirality at C11B        (Chiral SPGR)          R Verify
PLAT860_ALERT_3_G Number of Least-Squares Restraints .............         29 Note
PLAT868_ALERT_4_G ALERTS Due to the Use of _smtbx_masks Suppressed          ! Info
PLAT883_ALERT_1_G No Info/Value for _atom_sites_solution_primary .     Please Do !
PLAT909_ALERT_3_G Percentage of I>2sig(I) Data at Theta(Max) Still        62% Note
PLAT910_ALERT_3_G Missing # of FCF Reflection(s) Below Theta(Min).          4 Note
PLAT933_ALERT_2_G Number of OMIT Records in Embedded .res File ...          7 Note
```

```
   0 ALERT level A = Most likely a serious problem - resolve or explain
   5 ALERT level B = A potentially serious problem, consider carefully
  27 ALERT level C = Check. Ensure it is not caused by an omission or oversight
  65 ALERT level G = General information/check it is not something unexpected

   2 ALERT type 1 CIF construction/syntax error, inconsistent or missing data
  25 ALERT type 2 Indicator that the structure model may be wrong or deficient
  14 ALERT type 3 Indicator that the structure quality may be low
  55 ALERT type 4 Improvement, methodology, query or suggestion
   1 ALERT type 5 Informative message, check
```

Validation response form

Please find below a validation response form (VRF) that can be filled in and pasted into your CIF.

```
# start Validation Reply Form
_vrf_THETM01_vs3arepro
;
PROBLEM: The value of sine(theta_max)/wavelength is less than 0.575
RESPONSE: ...
;
# end Validation Reply Form
```

It is advisable to attempt to resolve as many as possible of the alerts in all categories. Often the minor alerts point to easily fixed oversights, errors and omissions in your CIF or refinement strategy, so attention to these fine details can be worthwhile. In order to resolve some of the more serious problems it may be necessary to carry out additional measurements or structure refinements. However, the purpose of your study may justify the reported deviations and the more serious of these should normally be commented upon in the discussion or experimental section of a paper or in the "special_details" fields of the CIF. checkCIF was carefully designed to identify outliers and unusual parameters, but every test has its limitations and alerts that are not important in a particular case may appear. Conversely, the absence of alerts does not guarantee there are no aspects of the results needing attention. It is up to the individual to critically assess their own results and, if necessary, seek expert advice.

Publication of your CIF in IUCr journals

A basic structural check has been run on your CIF. These basic checks will be run on all CIFs submitted for publication in IUCr journals (*Acta Crystallographica*, *Journal of Applied Crystallography*, *Journal of Synchrotron Radiation*); however, if you intend to submit to *Acta Crystallographica Section C* or *E* or *IUCrData*, you should make sure that full publication checks are run on the final version of your CIF prior to submission.

Publication of your CIF in other journals

Please refer to the *Notes for Authors* of the relevant journal for any special instructions relating to CIF submission.

PLATON version of 07/08/2019; check.def file version of 30/07/2019

Datablock vs3arepro - ellipsoid plot

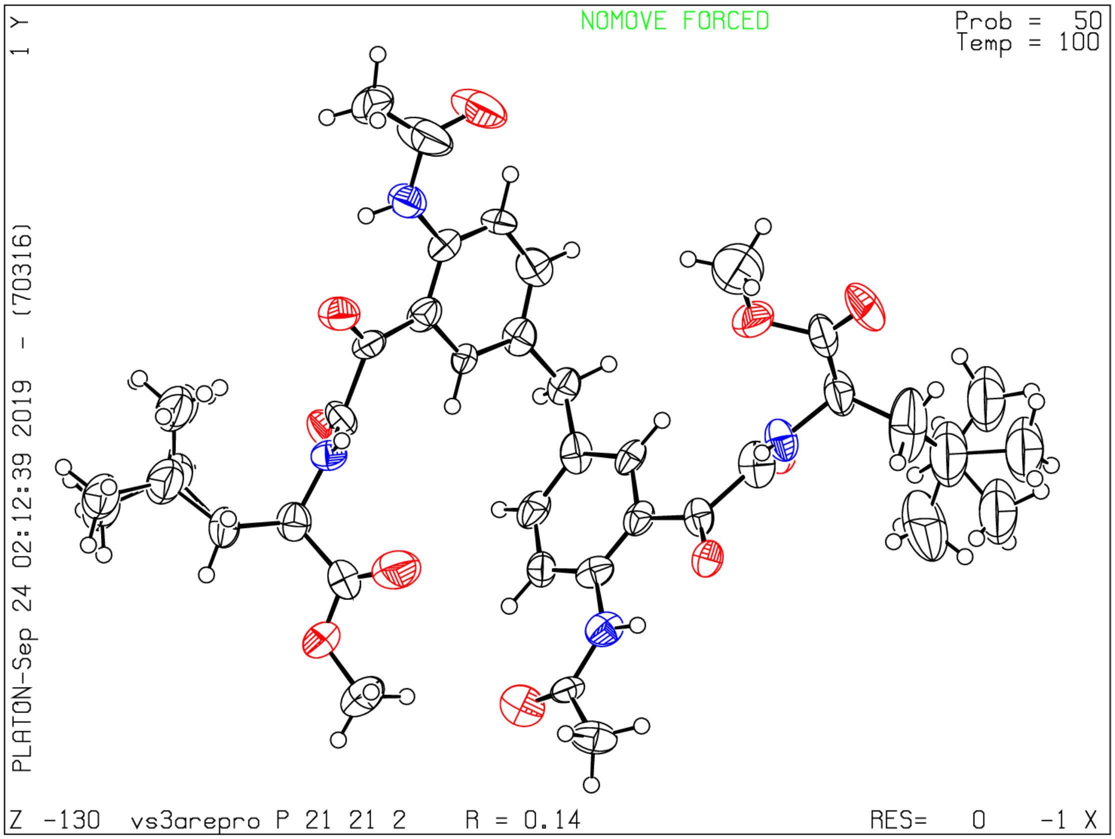

checkCIF/PLATON report

Structure factors have been supplied for datablock(s) vs4repro

THIS REPORT IS FOR GUIDANCE ONLY. IF USED AS PART OF A REVIEW PROCEDURE FOR PUBLICATION, IT SHOULD NOT REPLACE THE EXPERTISE OF AN EXPERIENCED CRYSTALLOGRAPHIC REFEREE.

No syntax errors found. CIF dictionary Interpreting this report

Datablock: vs4repro

```
Bond precision:  C-C = 0.0056 A          Wavelength=0.71073

Cell:            a=25.705(5)     b=5.020(1)          c=30.114(6)
                 alpha=90        beta=113.79(3)      gamma=90
Temperature:     100 K

                Calculated                 Reported
Volume          3555.7(15)                 3555.7(14)
Space group     C 2                        C 1 2 1
Hall group      C 2y                       C 2y
Moiety formula  C33 H40 N4 O10 S2          0.5(C33 H40 N4 O10 S2)
Sum formula     C33 H40 N4 O10 S2          C16.50 H20 N2 O5 S
Mr              716.81                     358.40
Dx,g cm-3       1.339                      1.339
Z               4                          8
Mu (mm-1)       0.211                      0.211
F000            1512.0                     1512.0
F000'           1513.73
h,k,lmax        28,5,34                    28,5,34
Nref            5495[ 3108]                5460
Tmin,Tmax       0.996,0.997
Tmin'           0.996

Correction method= Not given

Data completeness= 1.76/0.99      Theta(max)= 23.858

R(reflections)= 0.0366( 4781)     wR2(reflections)= 0.0862( 5460)

S = 1.038                  Npar= 450
```

The following ALERTS were generated. Each ALERT has the format
test-name_ALERT_alert-type_alert-level.
Click on the hyperlinks for more details of the test.

Alert level B

```
THETM01_ALERT_3_B  The value of sine(theta_max)/wavelength is less than 0.575
            Calculated sin(theta_max)/wavelength =    0.5691
```

Author Response: Crystals were ultrathin which diffracted very weakly at high angles even in the synchrotron beam at Australian MX1 beam line.

Alert level C

```
PLAT089_ALERT_3_C Poor Data / Parameter Ratio (Zmax < 18) ........       6.88 Note
PLAT340_ALERT_3_C Low Bond Precision on  C-C Bonds ...............    0.00564 Ang.
PLAT369_ALERT_2_C Long   C(sp2)-C(sp2) Bond  C9A      - C10A     .       1.55 Ang.
PLAT369_ALERT_2_C Long   C(sp2)-C(sp2) Bond  C9B      - C10B     .       1.54 Ang.
PLAT911_ALERT_3_C Missing FCF Refl Between Thmin & STh/L=    0.569         10 Report
PLAT913_ALERT_3_C Missing # of Very Strong Reflections in FCF ....          4 Note
```

Alert level G

```
PLAT007_ALERT_5_G Number of Unrefined Donor-H Atoms ..............          4 Report
PLAT042_ALERT_1_G Calc. and Reported MoietyFormula Strings  Differ     Please Check
PLAT045_ALERT_1_G Calculated and Reported Z Differ by a Factor ...       0.50 Check
PLAT300_ALERT_4_G Atom Site Occupancy of H1MA       Constrained at        0.5 Check
PLAT300_ALERT_4_G Atom Site Occupancy of H1MB       Constrained at        0.5 Check
PLAT300_ALERT_4_G Atom Site Occupancy of H1NA       Constrained at        0.5 Check
PLAT300_ALERT_4_G Atom Site Occupancy of H1NB       Constrained at        0.5 Check
PLAT367_ALERT_2_G Long?  C(sp?)-C(sp?) Bond  C1M      - C4A      .       1.52 Ang.
PLAT367_ALERT_2_G Long?  C(sp?)-C(sp?) Bond  C1M      - C4A_a    .       1.52 Ang.
PLAT367_ALERT_2_G Long?  C(sp?)-C(sp?) Bond  C1N      - C4B      .       1.51 Ang.
PLAT367_ALERT_2_G Long?  C(sp?)-C(sp?) Bond  C1N      - C4B_b    .       1.51 Ang.
PLAT720_ALERT_4_G Number of Unusual/Non-Standard Labels ..........         10 Note
PLAT791_ALERT_4_G Model has Chirality at C11A        (Chiral SPGR)          S Verify
PLAT791_ALERT_4_G Model has Chirality at C11B        (Chiral SPGR)          S Verify
PLAT883_ALERT_1_G No Info/Value for _atom_sites_solution_primary .     Please Do !
PLAT909_ALERT_3_G Percentage of I>2sig(I) Data at Theta(Max) Still        74% Note
PLAT910_ALERT_3_G Missing # of FCF Reflection(s) Below Theta(Min).          1 Note
PLAT933_ALERT_2_G Number of OMIT Records in Embedded .res File ...          7 Note
PLAT978_ALERT_2_G Number C-C Bonds with Positive Residual Density.          4 Info
```

---

0 **ALERT level A** = Most likely a serious problem - resolve or explain
1 **ALERT level B** = A potentially serious problem, consider carefully
6 **ALERT level C** = Check. Ensure it is not caused by an omission or oversight
19 **ALERT level G** = General information/check it is not something unexpected

3 ALERT type 1 CIF construction/syntax error, inconsistent or missing data
8 ALERT type 2 Indicator that the structure model may be wrong or deficient
7 ALERT type 3 Indicator that the structure quality may be low
7 ALERT type 4 Improvement, methodology, query or suggestion
1 ALERT type 5 Informative message, check

It is advisable to attempt to resolve as many as possible of the alerts in all categories. Often the minor alerts point to easily fixed oversights, errors and omissions in your CIF or refinement strategy, so attention to these fine details can be worthwhile. In order to resolve some of the more serious problems it may be necessary to carry out additional measurements or structure refinements. However, the purpose of your study may justify the reported deviations and the more serious of these should normally be commented upon in the discussion or experimental section of a paper or in the "special_details" fields of the CIF. checkCIF was carefully designed to identify outliers and unusual parameters, but every test has its limitations and alerts that are not important in a particular case may appear. Conversely, the absence of alerts does not guarantee there are no aspects of the results needing attention. It is up to the individual to critically assess their own results and, if necessary, seek expert advice.

Publication of your CIF in IUCr journals

A basic structural check has been run on your CIF. These basic checks will be run on all CIFs submitted for publication in IUCr journals (*Acta Crystallographica*, *Journal of Applied Crystallography*, *Journal of Synchrotron Radiation*); however, if you intend to submit to *Acta Crystallographica Section C* or *E* or *IUCrData*, you should make sure that full publication checks are run on the final version of your CIF prior to submission.

Publication of your CIF in other journals

Please refer to the *Notes for Authors* of the relevant journal for any special instructions relating to CIF submission.

PLATON version of 07/08/2019; check.def file version of 30/07/2019

Datablock vs4repro - ellipsoid plot

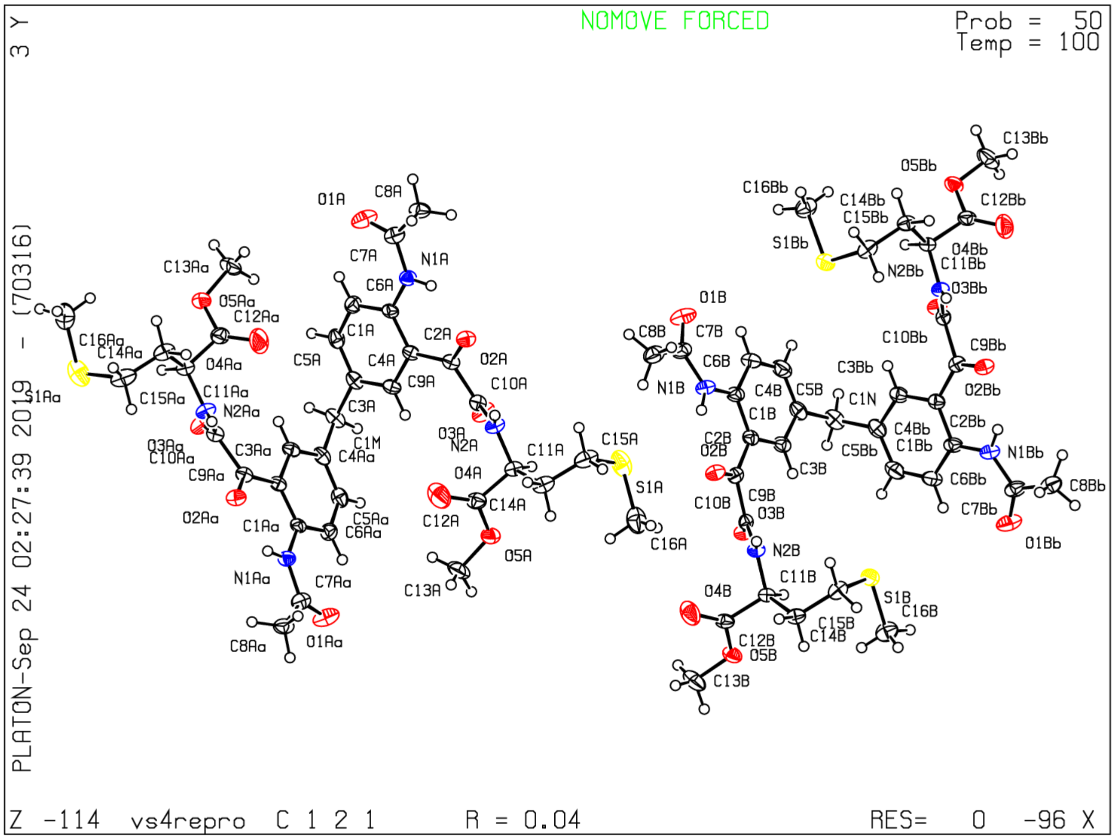

checkCIF/PLATON report

Structure factors have been supplied for datablock(s) vs5repro

THIS REPORT IS FOR GUIDANCE ONLY. IF USED AS PART OF A REVIEW PROCEDURE FOR PUBLICATION, IT SHOULD NOT REPLACE THE EXPERTISE OF AN EXPERIENCED CRYSTALLOGRAPHIC REFEREE.

No syntax errors found.   CIF dictionary   Interpreting this report

Datablock: vs5repro

```
Bond precision:  C-C = 0.0145 A          Wavelength=0.71073

Cell:            a=32.369(7)     b=4.869(1)          c=26.474(5)
                 alpha=90        beta=127.16(3)      gamma=90
Temperature:     100 K
```

	Calculated	Reported
Volume	3325.2(18)	3325.3(16)
Space group	C 2	C 1 2 1
Hall group	C 2y	C 2y
Moiety formula	C33 H34 N4 O10, C33 H38 N4 O10	C16.5 H19 N2 O5, C16.5 H17 N2 O5
Sum formula	C66 H72 N8 O20	C33 H36 N4 O10
Mr	1297.32	648.66
Dx,g cm-3	1.296	1.296
Z	2	4
Mu (mm-1)	0.097	0.097
F000	1368.0	1368.0
F000'	1368.73	
h,k,lmax	36,5,30	36,5,30
Nref	5143[2919]	4904
Tmin,Tmax	0.998,0.999	
Tmin'	0.998	

```
Correction method= Not given

Data completeness= 1.68/0.95       Theta(max)= 23.836

R(reflections)= 0.1237( 4712)      wR2(reflections)= 0.3870( 4904)

S = 2.026                  Npar= 384
```

The following ALERTS were generated. Each ALERT has the format
test-name_ALERT_alert-type_alert-level.
Click on the hyperlinks for more details of the test.

Alert level B

```
DIFMN02_ALERT_2_B  The minimum difference density is < -0.1*ZMAX*1.00
            _refine_diff_density_min given =     -1.141
            Test value =     -0.800
THETM01_ALERT_3_B  The value of sine(theta_max)/wavelength is less than 0.575
            Calculated sin(theta_max)/wavelength =    0.5686
```

Author Response: All the crystals were ultrathin and could only be measured at Australian synchrotron. Crystals diffracted only to lower values of theta.

```
PLAT029_ALERT_3_B _diffrn_measured_fraction_theta_full value Low .      0.955 Why?
```

Author Response: same as above

```
PLAT084_ALERT_3_B High wR2 Value (i.e. > 0.25) ..................       0.39 Report
```

Author Response: As a result of weak diffraction with asymmetric unit containing two molecules with disorders reflecting in higher values of wR2. One of the molecules takes two orientations which are very slightly displaced from each other. The limitation of data (not much of high angle data) and as a result inadequate modelling of disorder resulted in residual densities, low bond precisions and also inability to refine this molecule ansiotropically.

```
PLAT097_ALERT_2_B Large Reported Max.  (Positive) Residual Density       0.96 eA-3
```

Author Response: reasons as given above.

```
PLAT098_ALERT_2_B Large Reported Min.  (Negative) Residual Density      -1.14 eA-3
```

Author Response: reasons as given above

```
PLAT201_ALERT_2_B Isotropic non-H Atoms in Main Residue(s) .......          4 Report
              C1N     C11B    C13B    C14B
```

Author Response: reasons as given above

```
PLAT340_ALERT_3_B Low Bond Precision on  C-C Bonds ..............     0.0145 Ang.
```

Author Response: reasons as given above

```
PLAT430_ALERT_2_B Short Inter D...A Contact  O3A       ..N2A      .       2.73 Ang.
                                                    x,1+y,z  =      1_565 Check
PLAT934_ALERT_3_B Number of (Iobs-Icalc)/Sigma(W) > 10 Outliers ..          2 Check
```

Alert level C

```
DIFMN03_ALERT_1_C  The minimum difference density is < -0.1*ZMAX*0.75
            The relevant atom site should be identified.
DIFMX02_ALERT_1_C  The maximum difference density is > 0.1*ZMAX*0.75
            The relevant atom site should be identified.
GOODF01_ALERT_2_C  The least squares goodness of fit parameter lies
            outside the range 0.80 <> 2.00
            Goodness of fit given =      2.026
STRVA01_ALERT_2_C          Chirality of atom sites is inverted?
           From the CIF: _refine_ls_abs_structure_Flack    0.900
           From the CIF: _refine_ls_abs_structure_Flack_su    0.300
PLAT082_ALERT_2_C High R1 Value ..................................       0.12 Report
PLAT087_ALERT_2_C Unsatisfactory S value (Too High) ..............       2.03 Check
PLAT089_ALERT_3_C Poor Data / Parameter Ratio (Zmax < 18) ........       7.26 Note
PLAT220_ALERT_2_C Non-Solvent  Resd 1  C   Ueq(max)/Ueq(min) Range        4.6 Ratio
PLAT220_ALERT_2_C Non-Solvent  Resd 1  O   Ueq(max)/Ueq(min) Range        3.4 Ratio
PLAT222_ALERT_3_C Non-Solv.  Resd 1  H   Uiso(max)/Uiso(min) Range        4.4 Ratio
PLAT234_ALERT_4_C Large Hirshfeld Difference O5A'     --C12A     .       0.17 Ang.
PLAT241_ALERT_2_C High   'MainMol' Ueq as Compared to Neighbors of        C1N Check
PLAT241_ALERT_2_C High   'MainMol' Ueq as Compared to Neighbors of       C11B Check
PLAT242_ALERT_2_C Low    'MainMol' Ueq as Compared to Neighbors of       C11A Check
PLAT309_ALERT_2_C Single Bonded Oxygen (C-O > 1.3 Ang) ...........        O4" Check
PLAT309_ALERT_2_C Single Bonded Oxygen (C-O > 1.3 Ang) ...........       O2AA Check
PLAT329_ALERT_4_C Carbon Atom Hybridisation Unclear for ..........       C13B Check
PLAT369_ALERT_2_C Long   C(sp2)-C(sp2) Bond  C9A      - C10A     .       1.54 Ang.
PLAT790_ALERT_4_C Centre of Gravity not Within Unit Cell: Resd.  #          1 Note
             C33 H34 N4 O10
PLAT907_ALERT_2_C Flack x > 0.5, Structure Needs to be Inverted? .       0.90 Check
PLAT911_ALERT_3_C Missing FCF Refl Between Thmin & STh/L=    0.569        131 Report
PLAT913_ALERT_3_C Missing # of Very Strong Reflections in FCF ....         34 Note
PLAT918_ALERT_3_C Reflection(s) with I(obs) much Smaller I(calc) .          5 Check
PLAT939_ALERT_3_C Large Value of Not (SHELXL) Weight Optimized S .      13.02 Check
```

Alert level G

```
PLAT002_ALERT_2_G Number of Distance or Angle Restraints on AtSite         10 Note
PLAT007_ALERT_5_G Number of Unrefined Donor-H Atoms ..............          3 Report
PLAT032_ALERT_4_G Std. Uncertainty on Flack Parameter Value High .      0.300 Report
PLAT042_ALERT_1_G Calc. and Reported MoietyFormula Strings  Differ     Please Check
PLAT045_ALERT_1_G Calculated and Reported Z Differ by a Factor ...       0.50 Check
PLAT072_ALERT_2_G SHELXL First  Parameter in WGHT  Unusually Large       0.20 Report
PLAT152_ALERT_1_G The Supplied and Calc. Volume s.u. Differ by ...          2 Units
PLAT171_ALERT_4_G The CIF-Embedded .res File Contains EADP Records         12 Report
PLAT172_ALERT_4_G The CIF-Embedded .res File Contains DFIX Records          1 Report
PLAT176_ALERT_4_G The CIF-Embedded .res File Contains SADI Records          5 Report
PLAT300_ALERT_4_G Atom Site Occupancy of O1"       Constrained at         0.5 Check
PLAT300_ALERT_4_G Atom Site Occupancy of O2"       Constrained at         0.5 Check
PLAT300_ALERT_4_G Atom Site Occupancy of O3"       Constrained at         0.5 Check
PLAT300_ALERT_4_G Atom Site Occupancy of O0AA      Constrained at         0.5 Check
PLAT300_ALERT_4_G Atom Site Occupancy of O4"       Constrained at         0.5 Check
PLAT300_ALERT_4_G Atom Site Occupancy of O1AA      Constrained at         0.5 Check
PLAT300_ALERT_4_G Atom Site Occupancy of O4B       Constrained at         0.5 Check
PLAT300_ALERT_4_G Atom Site Occupancy of O5"       Constrained at         0.5 Check
PLAT300_ALERT_4_G Atom Site Occupancy of O2AA      Constrained at         0.5 Check
PLAT300_ALERT_4_G Atom Site Occupancy of O5B       Constrained at         0.5 Check
PLAT300_ALERT_4_G Atom Site Occupancy of N1"       Constrained at         0.5 Check
PLAT300_ALERT_4_G Atom Site Occupancy of N0AA      Constrained at         0.5 Check
PLAT300_ALERT_4_G Atom Site Occupancy of N2"       Constrained at         0.5 Check
PLAT300_ALERT_4_G Atom Site Occupancy of N2B       Constrained at         0.5 Check
PLAT300_ALERT_4_G Atom Site Occupancy of C1"       Constrained at         0.5 Check
PLAT300_ALERT_4_G Atom Site Occupancy of C1B       Constrained at         0.5 Check
```

PLAT300_ALERT_4_G Atom Site Occupancy of C2" Constrained at 0.5 Check
PLAT300_ALERT_4_G Atom Site Occupancy of C2B Constrained at 0.5 Check
PLAT300_ALERT_4_G Atom Site Occupancy of C3" Constrained at 0.5 Check
PLAT300_ALERT_4_G Atom Site Occupancy of C3B Constrained at 0.5 Check
PLAT300_ALERT_4_G Atom Site Occupancy of C4" Constrained at 0.5 Check
PLAT300_ALERT_4_G Atom Site Occupancy of C4B Constrained at 0.5 Check
PLAT300_ALERT_4_G Atom Site Occupancy of C5" Constrained at 0.5 Check
PLAT300_ALERT_4_G Atom Site Occupancy of C5B Constrained at 0.5 Check
PLAT300_ALERT_4_G Atom Site Occupancy of C6" Constrained at 0.5 Check
PLAT300_ALERT_4_G Atom Site Occupancy of C6B Constrained at 0.5 Check
PLAT300_ALERT_4_G Atom Site Occupancy of C7" Constrained at 0.5 Check
PLAT300_ALERT_4_G Atom Site Occupancy of C7B Constrained at 0.5 Check
PLAT300_ALERT_4_G Atom Site Occupancy of C8" Constrained at 0.5 Check
PLAT300_ALERT_4_G Atom Site Occupancy of C8B Constrained at 0.5 Check
PLAT300_ALERT_4_G Atom Site Occupancy of C9" Constrained at 0.5 Check
PLAT300_ALERT_4_G Atom Site Occupancy of C9B Constrained at 0.5 Check
PLAT300_ALERT_4_G Atom Site Occupancy of C10" Constrained at 0.5 Check
PLAT300_ALERT_4_G Atom Site Occupancy of C10B Constrained at 0.5 Check
PLAT300_ALERT_4_G Atom Site Occupancy of C12" Constrained at 0.5 Check
PLAT300_ALERT_4_G Atom Site Occupancy of C12B Constrained at 0.5 Check
PLAT300_ALERT_4_G Atom Site Occupancy of C15" Constrained at 0.5 Check
PLAT300_ALERT_4_G Atom Site Occupancy of C15B Constrained at 0.5 Check
PLAT300_ALERT_4_G Atom Site Occupancy of C16" Constrained at 0.5 Check
PLAT300_ALERT_4_G Atom Site Occupancy of C16B Constrained at 0.5 Check
PLAT300_ALERT_4_G Atom Site Occupancy of H1" Constrained at 0.5 Check
PLAT300_ALERT_4_G Atom Site Occupancy of H3" Constrained at 0.5 Check
PLAT300_ALERT_4_G Atom Site Occupancy of H3B Constrained at 0.5 Check
PLAT300_ALERT_4_G Atom Site Occupancy of H5" Constrained at 0.5 Check
PLAT300_ALERT_4_G Atom Site Occupancy of H5B Constrained at 0.5 Check
PLAT300_ALERT_4_G Atom Site Occupancy of H6" Constrained at 0.5 Check
PLAT300_ALERT_4_G Atom Site Occupancy of H1NB Constrained at 0.5 Check
PLAT300_ALERT_4_G Atom Site Occupancy of H6B Constrained at 0.5 Check
PLAT300_ALERT_4_G Atom Site Occupancy of H1NA Constrained at 0.5 Check
PLAT300_ALERT_4_G Atom Site Occupancy of H1NC Constrained at 0.5 Check
PLAT300_ALERT_4_G Atom Site Occupancy of H1ND Constrained at 0.5 Check
PLAT300_ALERT_4_G Atom Site Occupancy of H0AA Constrained at 0.5 Check
PLAT300_ALERT_4_G Atom Site Occupancy of H8BA Constrained at 0.5 Check
PLAT300_ALERT_4_G Atom Site Occupancy of H11B Constrained at 0.5 Check
PLAT300_ALERT_4_G Atom Site Occupancy of H8BB Constrained at 0.5 Check
PLAT300_ALERT_4_G Atom Site Occupancy of H8BC Constrained at 0.5 Check
PLAT300_ALERT_4_G Atom Site Occupancy of H13A Constrained at 0.5 Check
PLAT300_ALERT_4_G Atom Site Occupancy of H13B Constrained at 0.5 Check
PLAT300_ALERT_4_G Atom Site Occupancy of H13C Constrained at 0.5 Check
PLAT300_ALERT_4_G Atom Site Occupancy of H8"A Constrained at 0.5 Check
PLAT300_ALERT_4_G Atom Site Occupancy of H8"B Constrained at 0.5 Check
PLAT300_ALERT_4_G Atom Site Occupancy of H15A Constrained at 0.5 Check
PLAT300_ALERT_4_G Atom Site Occupancy of H15B Constrained at 0.5 Check
PLAT300_ALERT_4_G Atom Site Occupancy of H15C Constrained at 0.5 Check
PLAT300_ALERT_4_G Atom Site Occupancy of H15D Constrained at 0.5 Check
PLAT300_ALERT_4_G Atom Site Occupancy of H15E Constrained at 0.5 Check
PLAT300_ALERT_4_G Atom Site Occupancy of H15F Constrained at 0.5 Check
PLAT300_ALERT_4_G Atom Site Occupancy of H8"C Constrained at 0.5 Check
PLAT300_ALERT_4_G Atom Site Occupancy of H16A Constrained at 0.5 Check
PLAT300_ALERT_4_G Atom Site Occupancy of H16B Constrained at 0.5 Check
PLAT300_ALERT_4_G Atom Site Occupancy of H16C Constrained at 0.5 Check
PLAT300_ALERT_4_G Atom Site Occupancy of H16D Constrained at 0.5 Check
PLAT300_ALERT_4_G Atom Site Occupancy of H16E Constrained at 0.5 Check
PLAT300_ALERT_4_G Atom Site Occupancy of H16F Constrained at 0.5 Check
PLAT300_ALERT_4_G Atom Site Occupancy of O4A’ Constrained at 0.5 Check
PLAT300_ALERT_4_G Atom Site Occupancy of O5A’ Constrained at 0.5 Check
PLAT300_ALERT_4_G Atom Site Occupancy of O4A Constrained at 0.5 Check
PLAT300_ALERT_4_G Atom Site Occupancy of O5A Constrained at 0.5 Check

```
PLAT300_ALERT_4_G Atom Site Occupancy of C8        Constrained at       0.5 Check
PLAT300_ALERT_4_G Atom Site Occupancy of C13A      Constrained at       0.5 Check
PLAT300_ALERT_4_G Atom Site Occupancy of C15'      Constrained at       0.5 Check
PLAT300_ALERT_4_G Atom Site Occupancy of C15A      Constrained at       0.5 Check
PLAT300_ALERT_4_G Atom Site Occupancy of C16'      Constrained at       0.5 Check
PLAT300_ALERT_4_G Atom Site Occupancy of C16A      Constrained at       0.5 Check
PLAT300_ALERT_4_G Atom Site Occupancy of H1MA      Constrained at       0.5 Check
PLAT300_ALERT_4_G Atom Site Occupancy of H1MB      Constrained at       0.5 Check
PLAT300_ALERT_4_G Atom Site Occupancy of H8A       Constrained at       0.5 Check
PLAT300_ALERT_4_G Atom Site Occupancy of H8B       Constrained at       0.5 Check
PLAT300_ALERT_4_G Atom Site Occupancy of H8C       Constrained at       0.5 Check
PLAT300_ALERT_4_G Atom Site Occupancy of H13D      Constrained at       0.5 Check
PLAT300_ALERT_4_G Atom Site Occupancy of H13E      Constrained at       0.5 Check
PLAT300_ALERT_4_G Atom Site Occupancy of H13F      Constrained at       0.5 Check
PLAT300_ALERT_4_G Atom Site Occupancy of H14A      Constrained at       0.5 Check
PLAT300_ALERT_4_G Atom Site Occupancy of H14B      Constrained at       0.5 Check
PLAT300_ALERT_4_G Atom Site Occupancy of H15G      Constrained at       0.5 Check
PLAT300_ALERT_4_G Atom Site Occupancy of H15H      Constrained at       0.5 Check
PLAT300_ALERT_4_G Atom Site Occupancy of H15I      Constrained at       0.5 Check
PLAT300_ALERT_4_G Atom Site Occupancy of H15J      Constrained at       0.5 Check
PLAT300_ALERT_4_G Atom Site Occupancy of H15K      Constrained at       0.5 Check
PLAT300_ALERT_4_G Atom Site Occupancy of H15L      Constrained at       0.5 Check
PLAT300_ALERT_4_G Atom Site Occupancy of H16G      Constrained at       0.5 Check
PLAT300_ALERT_4_G Atom Site Occupancy of H16H      Constrained at       0.5 Check
PLAT300_ALERT_4_G Atom Site Occupancy of H16I      Constrained at       0.5 Check
PLAT300_ALERT_4_G Atom Site Occupancy of H16J      Constrained at       0.5 Check
PLAT300_ALERT_4_G Atom Site Occupancy of H16K      Constrained at       0.5 Check
PLAT300_ALERT_4_G Atom Site Occupancy of H16L      Constrained at       0.5 Check
PLAT301_ALERT_3_G Main Residue  Disorder .............(Resd  1  )       85% Note
PLAT301_ALERT_3_G Main Residue  Disorder .............(Resd  2  )       21% Note
PLAT309_ALERT_2_G Single Bonded Oxygen (C-O > 1.3 Ang) ..........       O2" Check
PLAT367_ALERT_2_G Long?  C(sp?)-C(sp?) Bond  C11B     - C14B     .      1.53 Ang.
PLAT367_ALERT_2_G Long?  C(sp?)-C(sp?) Bond  C1M      - C4A      .      1.51 Ang.
PLAT367_ALERT_2_G Long?  C(sp?)-C(sp?) Bond  C1M      - C4A_b    .      1.51 Ang.
PLAT398_ALERT_2_G Deviating  C-O-C    Angle From 120 for O5"          108.1 Degree
PLAT398_ALERT_2_G Deviating  C-O-C    Angle From 120 for O5B          101.3 Degree
PLAT720_ALERT_4_G Number of Unusual/Non-Standard Labels .........        22 Note
PLAT773_ALERT_2_G Check long C-C Bond in CIF: C11B     --C12B           1.74 Ang.
PLAT779_ALERT_4_G Suspect or Irrelevant (Bond) Angle(s) in CIF . #        43 Check
              C4B  -C1N  -C4"     2.453   1.555   2.453             27.50 Deg.
PLAT790_ALERT_4_G Centre of Gravity not Within Unit Cell: Resd.  #         2 Note
              C33 H38 N4 O10
PLAT811_ALERT_5_G No ADDSYM Analysis: Too Many Excluded Atoms ....         ! Info
PLAT860_ALERT_3_G Number of Least-Squares Restraints ............          4 Note
PLAT909_ALERT_3_G Percentage of I>2sig(I) Data at Theta(Max) Still       90% Note
PLAT978_ALERT_2_G Number C-C Bonds with Positive Residual Density.         2 Info
```

---

0 **ALERT level A** = Most likely a serious problem - resolve or explain
10 **ALERT level B** = A potentially serious problem, consider carefully
24 **ALERT level C** = Check. Ensure it is not caused by an omission or oversight
132 **ALERT level G** = General information/check it is not something unexpected

5 ALERT type 1 CIF construction/syntax error, inconsistent or missing data
28 ALERT type 2 Indicator that the structure model may be wrong or deficient
15 ALERT type 3 Indicator that the structure quality may be low
116 ALERT type 4 Improvement, methodology, query or suggestion
2 ALERT type 5 Informative message, check

Validation response form

Please find below a validation response form (VRF) that can be filled in and pasted into your CIF.

```
# start Validation Reply Form
_vrf_DIFMN02_vs5repro
;
PROBLEM: The minimum difference density is < -0.1*ZMAX*1.00
RESPONSE: ...
;
_vrf_PLAT430_vs5repro
;
PROBLEM: Short Inter D...A Contact  O3A      ..N2A      .       2.73 Ang.
RESPONSE: ...
;
_vrf_PLAT934_vs5repro
;
PROBLEM: Number of (Iobs-Icalc)/Sigma(W) > 10 Outliers ..          2 Check
RESPONSE: ...
;
# end Validation Reply Form
```

It is advisable to attempt to resolve as many as possible of the alerts in all categories. Often the minor alerts point to easily fixed oversights, errors and omissions in your CIF or refinement strategy, so attention to these fine details can be worthwhile. In order to resolve some of the more serious problems it may be necessary to carry out additional measurements or structure refinements. However, the purpose of your study may justify the reported deviations and the more serious of these should normally be commented upon in the discussion or experimental section of a paper or in the "special_details" fields of the CIF. checkCIF was carefully designed to identify outliers and unusual parameters, but every test has its limitations and alerts that are not important in a particular case may appear. Conversely, the absence of alerts does not guarantee there are no aspects of the results needing attention. It is up to the individual to critically assess their own results and, if necessary, seek expert advice.

Publication of your CIF in IUCr journals

A basic structural check has been run on your CIF. These basic checks will be run on all CIFs submitted for publication in IUCr journals (*Acta Crystallographica*, *Journal of Applied Crystallography*, *Journal of Synchrotron Radiation*); however, if you intend to submit to *Acta Crystallographica Section C* or *E* or *IUCrData*, you should make sure that full publication checks are run on the final version of your CIF prior to submission.

Publication of your CIF in other journals

Please refer to the *Notes for Authors* of the relevant journal for any special instructions relating to CIF submission.

PLATON version of 07/08/2019; check.def file version of 30/07/2019

Datablock vs5repro - ellipsoid plot

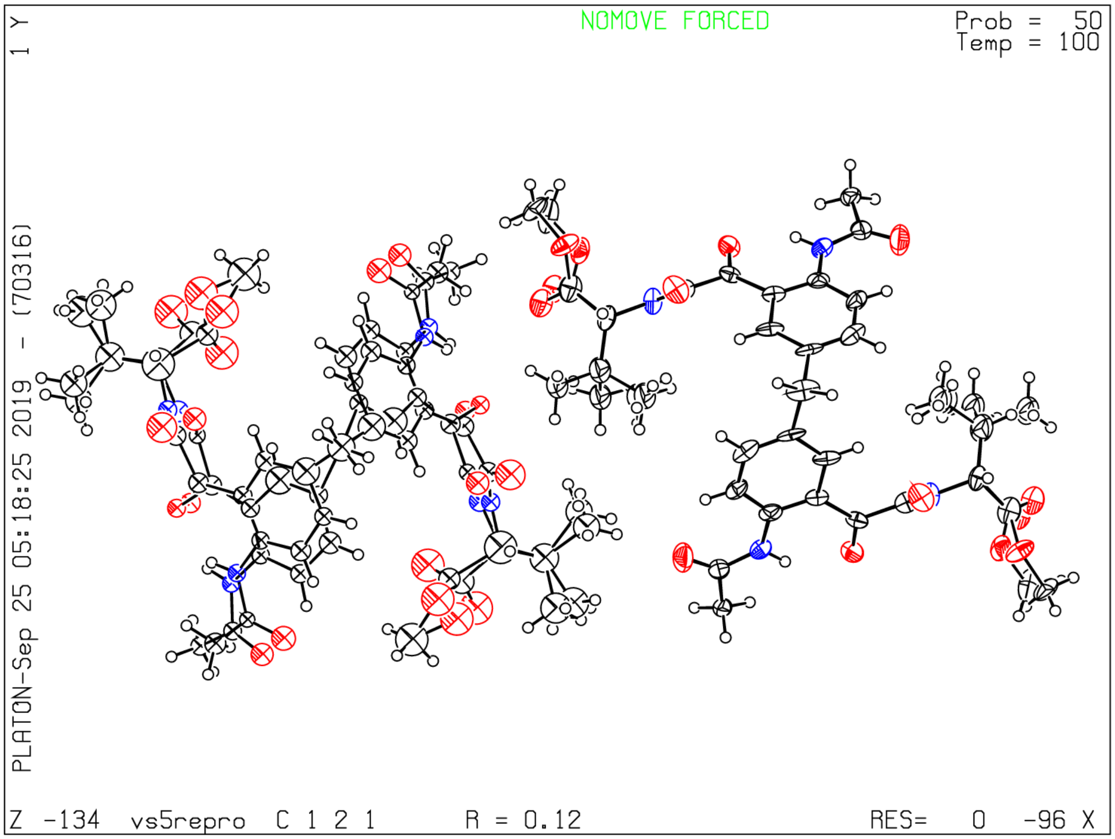

Supplement: Supplementary file 1 [file molecules-24-04343-s001.pdf]
